# Measuring population ageing: an analysis of the Global Burden of Disease Study 2017

**DOI:** 10.1016/S2468-2667(19)30019-2

**Published:** 2019-03-06

**Authors:** Angela Y Chang, Vegard F Skirbekk, Stefanos Tyrovolas, Nicholas J Kassebaum, Joseph L Dieleman

**Affiliations:** a Institute for Health Metrics and Evaluation, University of Washington, Seattle, WA, USA; b Department of Anesthesiology & Pain Medicine, Seattle Children's Hospital, University of Washington, Seattle, WA, USA; c Centre for Fertility and Health, Norwegian Institute of Public Health, Oslo, Norway; d Columbia Aging Center, Columbia University, New York, NY, USA; e Parc Sanitari Sant Joan de Déu, Universitat de Barcelona, Barcelona, Spain; f Instituto de Salud Carlos III, Centro de Investigación Biomédica en Red de Salud Mental, CIBERSAM, Madrid, Spain

## Abstract

**Background:**

Traditional metrics for population health ageing tend not to differentiate between extending life expectancy and adding healthy years. A population ageing metric that reflects both longevity and health status, incorporates a comprehensive range of diseases, and allows for comparisons across countries and time is required to understand the progression of ageing and to inform policies.

**Methods:**

Using the Global Burden of Diseases, Injuries, and Risk Factors Study 2017, we developed a metric that reflects age-related morbidity and mortality at the population level. First, we identified a set of age-related diseases, defined as diseases with incidence rates among the adult population increasing quadratically with age, and measured their age-related burden, defined as the sum of disability-adjusted life-years (DALYs) of these diseases among adults. Second, we estimated age-standardised age-related health burden across 195 countries between 1990 and 2017. Using global average 65-year-olds as the reference population, we calculated the equivalent age in terms of age-related disease burden for all countries. Third, we analysed how the changes in age-related burden during the study period relate to different factors with a decomposition analysis. Finally, we describe how countries with similar levels of overall age-related burden experience different onsets of ageing. We represent the uncertainty of our estimates by calculating uncertainty intervals (UI) from 1000 draw-level estimates for each disease, country, year, and age.

**Findings:**

92 diseases were identified as age related, accounting for 51·3% (95% UI 48·5–53·9) of all global burden among adults in 2017. Across the Socio-demographic Index (SDI), the rate of age-related burden ranged from 137·8 DALYs (128·9–148·3) per 1000 adults in high SDI countries to 265·9 DALYs (251·0–280·1) in low SDI countries. The equivalent age to average 65-year-olds globally spanned from 76·1 years (75·6–76·7) in Japan to 45·6 years (42·6–48·2) in Papua New Guinea. Age-standardised age-related disease rates have decreased over time across all SDI levels and regions between 1990 and 2017, mainly due to decreases in age-related case fatality and disease severity. Even among countries with similar age-standardised death rates, large differences in the onset and patterns of accumulating age-related burden exist.

**Interpretation:**

The new metric facilitates the shift from thinking not just about chronological age but the health status and disease severity of ageing populations. Our findings could provide inputs into policymaking by identifying key drivers of variation in the ageing burden and resources required for addressing the burden.

**Funding:**

National Institute on Aging of the National Institutes of Health.

## Introduction

Governments worldwide are moving with urgency to introduce policies that address population ageing. Whether increased longevity is an opportunity or a threat to the stability of societies largely depends not only on whether populations are living longer but whether they are experiencing the negative health effects of ageing—a progressive loss of physical, mental, and cognitive integrity, leading to impaired functions and increased vulnerability to morbidity and mortality.^1^, ^2^ It is therefore crucial that we measure the extent to which ageing and age-related disease burden occurs in a population over time to inform better policies.

In existing public health literature, there are two main approaches to measuring ageing. The first and most common set of ageing metrics involves measuring changes in the age structure but fails to capture the level of morbidity of the population.^3^ They include metrics such as the shift in the population's age distribution toward older ages,^4^ increase in the population's median age,^5^ increase in average life expectancy,^4^, ^6^ number of remaining years left to live, and changes in the ratio between what are classed as older and working age groups.^7^ The second main set of literature focuses on measuring the functional status of older populations, using objective measures such as biomarkers,^8^ frailty,^9^, ^10^, ^11^ and cognitive functioning^12^ or subjective measures such as self-reported health and instrumental limitations of activities of daily living. These studies, in our view, more accurately capture the health status of individuals and reflect the heterogeneity in how people age. However, the studies often involve complex, resource-intensive study designs, are conducted among a relatively small sample, and might not be generalisable or comparable across study settings. The measures are often not identical or similarly understood and tested across study populations or over time, making these studies and the longitudinal waves difficult to compare or to be aggregated into population-level metrics.^13^, ^14^, ^15^ Another challenge is that none of these studies and measures take most diseases and their severity into account.

**Research in context**

**Evidence before this study**

We searched PubMed, Google Scholar, and publicly available grey literature for the terms “ageing”, functioning”, “frailty”, “measurement”, and “metric” on May 1, 2018, without language or field restrictions. We further consulted with well recognised experts in this field for literature recommendations. We found that in both academic and policy communities, population ageing, including the measurement and promotion of concepts such as so-called healthy or successful ageing has been an ongoing interest for several decades. Some of the most common metrics of population ageing entail descriptions of increased longevity (such as rising life expectancy at older ages) and changes in the distribution of age groups (such as median age, proportion of older populations, and old-age dependency ratio). Many define old age by setting a universal age threshold, often originating from social policies or norms such as retirement ages, and consider all individuals above this age threshold as older populations. WHO and the UN Population Division commonly apply the age thresholds of age 60, 65, or 70 years. However, the age at which one is considered an older person should be based on objective measures and should take into account the health status of the individuals, including whether population ageing is accompanied by improvements or deterioration in health.

**Added value of this study**

The Global Burden of Diseases, Injuries, and Risk Factors Study (GBD) provides estimation of health burden for a comprehensive and comparable list of diseases across time and geographies and offers a framework through which the limitations in current measures of population ageing can be addressed. First, using disability-adjusted life-years of age-related diseases of all adults to measure population ageing, our approach captures both the longevity and health of populations and avoids setting arbitrary age thresholds to define older populations. Second, given the consistent and systematic approaches taken by GBD in measuring health outcomes, we can compare changes in age-related disease burden across countries and over time and assess different factors that have led to changes over time. Finally, using GBD's age-specific health estimates, we are able to capture at which ages the burden of age-related diseases start to accumulate and compare results among countries with similar levels of overall age-related disease burden to explore whether some have an earlier onset of ageing burden than others.

**Implications of all the available evidence**

Globally, age-standardised age-related disease burden among adults has declined since 1990. While the number of adults and the median age of adults have increased over time, we estimated large declines in case fatality and disease severity of age-related diseases. We also found wide variations in the levels, trends, and the onsets of age-related disease burden across countries. For example, 76-year-olds in Japan and 46-year-olds in Papua New Guinea have the same level of age-related disease burden as the global average 65-year-olds. Despite not living as long, we find that adults in low SDI countries still experience high burden of age-related diseases. From the perspective of improving the measurement of population ageing, our method and findings provide a nuanced and systematic measure by considering both longevity and health of adults as well as the speed at which age-related disease burden accumulates. From the perspective of providing policy recommendations for addressing challenges in population ageing, our results highlight the wide variation in the patterns of age-related disease burden across country and time and the factors associated with these changes. Identifying exemplar countries that have been more successful in reducing the levels of age-related disease burden over time or delaying the accumulation of age-related disease burden to later ages and understanding how they reached their achievements can inform policy efforts to address the challenges of population ageing worldwide.

This study aims to fill the gap between existing population-level and individual-level ageing metrics and to introduce an ageing metric that informs not only longevity but also health status and disease severity at the population level.

## Methods

### Data sources

This study draws from the Global Burden of Diseases, Injuries, and Risk Factors Study (GBD) 2017 results for 195 countries between 1990 and 2017. We analysed disease burden estimates for the 293 most detailed diseases and injuries under the GBD cause hierarchy. Data used in this study, including rates and numbers of incident and prevalent cases, disability-adjusted life-years (DALYs), and deaths, can be downloaded from GBD Compare. Detailed descriptions of GBD 2017 can be found elsewhere.^16^, ^17^

### Defining age-related diseases and burden

Following guidance from established literature on ageing,^10^, ^12^, ^18^, ^19^, ^20^ we defined age-related diseases as diseases with incidence rates increasing quadratically with age among the adult population. To identify age-related diseases, we applied a two-step regression framework to the 2017 global incidence rates, described in detail in the appendix. For a small subset of GBD causes that do not have incidence estimates, we relied on prevalence estimates and applied the same framework. The adult population is defined as people aged 25 years and older.^21^ We additionally did sensitivity analyses around diseases that have been considered age related by some studies but did not meet our criteria for age-related diseases and present the findings in the appendix.

Age-related disease burden is the sum of all DALYs of age-related diseases among adults. We estimated age-related disease burden, the proportion of age-related disease burden of total health burden, and age-standardised rates per 1000 adults (to allow comparison across time and countries by adjusting for population size and age structure, using the method described in the appendix) for all countries, quintiles of Sociodemographic Index (SDI; a measure of overall development consisting of income per capita, average years of education, and total fertility rate under 25 years^22^), and GBD super-regions. The latter two are aggregated from country-specific estimates.^12^, ^13^, ^14^

### Estimating the equivalent age of populations across countries and time compared with global 65-year-olds in 2017

We estimated the age of populations in which countries have the same health burden as global average 65-year-olds. To approximate an age of equivalence, we set the age-related disease burden rate of global average 65-year-olds in 2017 as the reference and identified the age groups in each country with the closest age-related disease burden rate to the reference. More detail can be found in the appendix.

### Analysing the changes in age-standardised age-related disease burden, 1990–2017

For all SDI levels and GBD super-regions, we first measured the change in age-standardised age-related disease burden rate (absolute and relative) from 1990 to 2017. Second, to understand the factors associated with the changes in the absolute number of age-related DALYs during this period, we constructed a decomposition analysis by expressing age-related disease burden as the product of four factors: (1) size of the adult population, (2) age structure of the adult population, (3) prevalence of age-related diseases, and (4) case fatality and disease severity of age-related diseases, which correspond to the four terms shown in the sum below:

$${\text{DALY}}_{y}=\sum_{a,d}{\text{pop size}}_{y}\cdot \frac{{\text{pop age}}_{ay}}{{\text{pop size}}_{y}}\cdot \frac{{\text{preval}}_{ady}}{{\text{pop age}}_{ay}}\cdot \frac{{\text{DALY}}_{ady}}{{\text{preval}}_{ady}}$$

where *a* represents 5-year age groups of adult population aged 25 years and older (up to the final unbounded age group of 90 years and older), *d* represents age-related disease, and *y* represents the year. The DALYs associated with each of the four components sum up to the total age-related disease burden in the year of interest. This decomposition approach measures the additive contribution of each factor to changes in the age-related disease burden between 1990 and 2017 for each age group and disease and does not capture the interactions between factors.^23^ A detailed explanation of the decomposition analysis can be found in the appendix.

### Measuring the onset of age-related burden through cumulative age-related deaths

In addition to estimating variation in age-related disease burden across populations, we also assessed when the accumulation of age-related disease burden occurs^24^ among countries with similar levels of age-related disease burden. It would theoretically be preferable for age-related disease burden to occur at later rather than earlier ages to reduce years spent living with disability. To identify countries that have slower onset of ageing—ie, later accumulation of age-related disease burden—we used deaths instead of DALYs as the measure of health burden. A DALY is, by definition, the sum of years of life lost and years lived with disability. Within the GBD framework, the years of life lost associated with the disease are assigned to the age at which the disease occurs, whereas the years lived with disability are spread across all remaining life years. Therefore, the exact onset of age-related disease (and thus the occurrence of age-related disease burden) is crucial to measure the onset of age-related disease burden; we therefore only looked at age-related deaths in this analysis because they occur at a specific timepoint. For each age group starting at age 25 years, we estimated the cumulative, age-standardised age-related death rates. Countries with the lowest cumulative age-related death rate for each age group in 2017 were empirically identified and set as frontiers. To facilitate comparison of the ageing patterns across countries and to the frontier countries, we further scaled all cumulative death rates to a range of 0–1, using the country-specific cumulative death rate of age group 80–84 years (the highest observed life expectancy at birth) as the reference point.

### Uncertainty analysis

Final estimates and uncertainty intervals (UI) were calculated from 1000 draw-level estimates for each disease, country, year, and age. 95% UIs were based on the 2·5th and 97·5th percentiles of the draws for each estimate. All analyses were done using R, version 3.4.1.

### Role of the funding source

The funder of the study had no role in study design, data collection, data analysis, data interpretation, or writing of the report. The corresponding author had full access to all the data in the study and had final responsibility for the decision to submit for publication.

## Results

Among the 293 GBD causes, 92 (31·4%) were identified as age-related diseases (panel). A detailed flowchart of the steps taken to include and exclude diseases is shown in the appendix. Among the 92 diseases, five are communicable diseases, six are injuries, and 81 are non-communicable diseases. Among the non-communicable diseases, 13 are cardiovascular diseases, 35 cancers, six chronic respiratory diseases, five digestive diseases, three diabetes and kidney diseases, three neurological disorders, seven sense organ disorders, five skin and subcutaneous diseases, and four other non-communicable diseases. Globally, the age-related diseases with the most deaths and DALYs were ischaemic heart disease, intracerebral haemorrhage, and chronic obstructive pulmonary disease. Compared with diseases that were not identified as being age related, age-related disease burden includes larger proportions of non-communicable diseases such as cardiovascular diseases (38·4%, 95% UI 37·1–39·7), neoplasms (22·6%, 21·6–23·6), chronic respiratory disorders (9·8%, 9·1–10·4), and sense organ disorders (5·9%, 4·3–7·7; appendix). None of the musculoskeletal and mental and substance disorders were included as age-related diseases.

Panel **92 age-related diseases, by broader disease categories**

**Cardiovascular diseases**

Atrial fibrillation and flutter; endocarditis; hypertensive heart disease; intracerebral haemorrhage; ischaemic heart disease; ischaemic stroke; myocarditis; non-rheumatic calcific aortic valve disease; non-rheumatic degenerative mitral valve disease; other cardiomyopathy; other cardiovascular and circulatory diseases; other non-rheumatic valve diseases; peripheral artery disease

**Chronic respiratory diseases**

Asbestosis; chronic obstructive pulmonary disease; coal worker pneumoconiosis; interstitial lung disease and pulmonary sarcoidosis; other pneumoconiosis; silicosis

**Communicable, maternal, neonatal, and nutritional diseases**

Diarrhoeal diseases; encephalitis; lower respiratory infections; pneumococcal meningitis; trachoma

**Diabetes and kidney diseases**

Chronic kidney disease due to type 2 diabetes mellitus; chronic kidney disease due to glomerulonephritis; chronic kidney disease due to other and unspecified causes

**Digestive diseases**

Cirrhosis due to NASH; pancreatitis; paralytic ileus and intestinal obstruction; peptic ulcer disease; vascular intestinal disorders

**Injuries**

Drowning; environmental heat and cold exposure; falls; foreign body in other body part; other transport injuries; other unintentional injuries

**Neoplasms**

Acute lymphoid leukaemia; acute myeloid leukaemia; benign and in-situ intestinal neoplasms; bladder cancer; brain and nervous system cancer; breast cancer; chronic lymphoid leukaemia; chronic myeloid leukaemia; colon and rectum cancer; gallbladder and biliary tract cancer; Hodgkin lymphoma; kidney cancer; larynx cancer; lip and oral cavity cancer; liver cancer due to NASH; liver cancer due to alcohol use; liver cancer due to hepatitis C; malignant skin melanoma; mesothelioma; multiple myeloma; myelodysplastic, myeloproliferative, and other hematopoietic neoplasms; non-Hodgkin lymphoma; non-melanoma skin cancer (basal-cell carcinoma); non-melanoma skin cancer (squamous-cell carcinoma); oesophageal cancer; other benign and in-situ neoplasms; other leukaemia; other malignant neoplasms; ovarian cancer; pancreatic cancer; prostate cancer; stomach cancer; thyroid cancer; tracheal, bronchus, and lung cancer; uterine cancer

**Neurological disorders**

Alzheimer's disease and other dementias; motor neuron disease; Parkinson's disease

**Other non-communicable diseases**

Congenital musculoskeletal and limb anomalies; digestive congenital anomalies; endocrine, metabolic, blood, and immune disorders; other haemoglobinopathies and haemolytic anaemias

**Sense organ diseases**

Age-related and other hearing loss; age-related macular degeneration; cataract; glaucoma; other sense organ diseases; other vision loss; refraction disorders

**Skin and subcutaneous diseases**

Cellulitis; decubitus ulcer; fungal skin diseases; other skin and subcutaneous diseases; pyoderma

NASH=non-alcoholic steatohepatitis.

**Figure 1:**
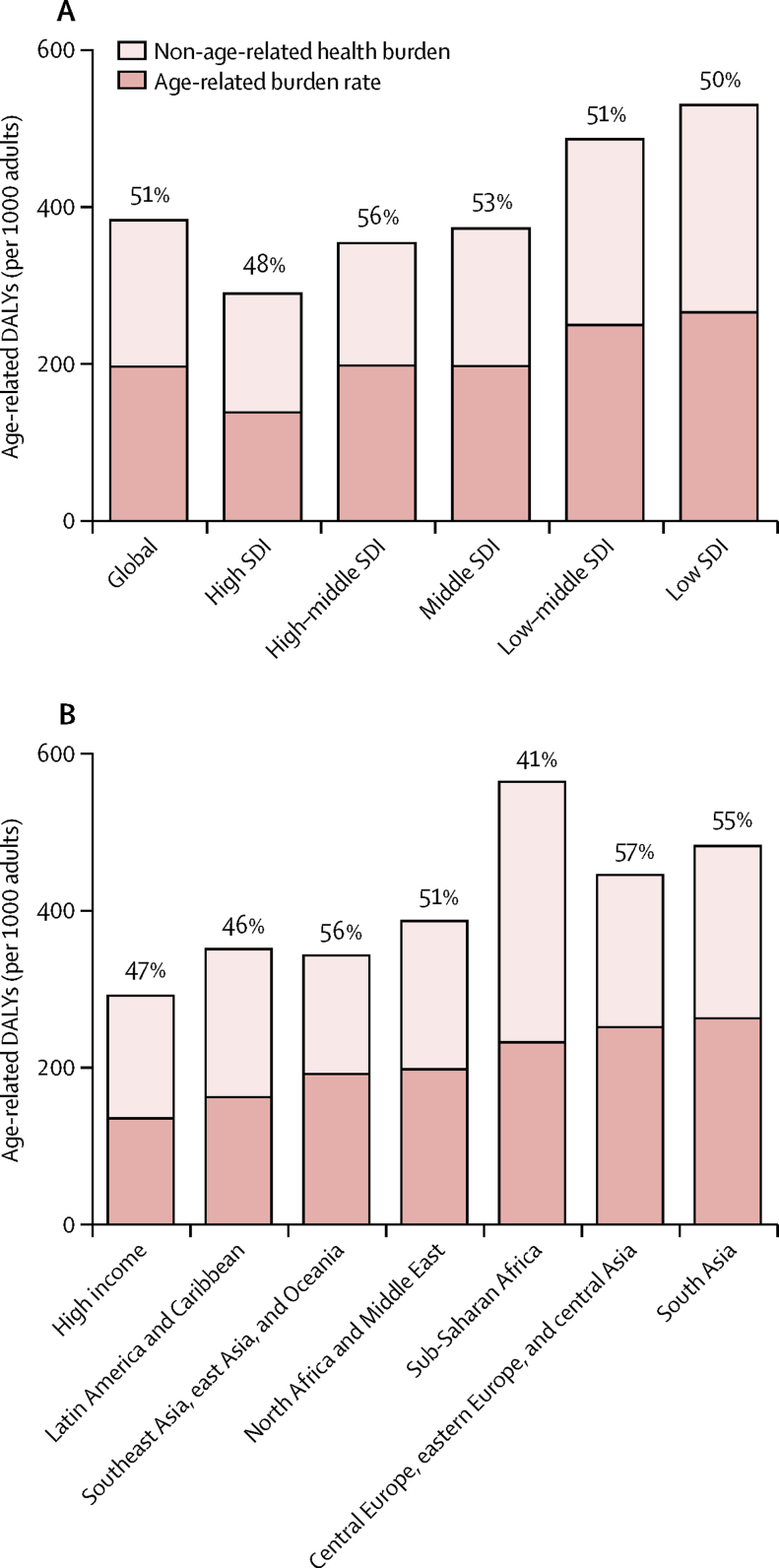
Age-standardised rate and proportion of all health burden among the adult population that is age related, by SDI quintile (A) and GBD super-region (B) in 2017 All burden is shown split into age-standardised age-related burden (per thousand adults; dark bars) and age-standardised non-age-related burden (light bars). The percentages given represent the proportion of age-related burden among all burden. DALYs=disability-adjusted life-years. GBD=Global Burden of Diseases, Injuries, and Risk Factors Study. SDI=Socio-demographic Index.

Globally, the rate of age-related burden ranged from 137·8 DALYs (128·9–148·3) per 1000 adults in high SDI countries to 265·9 DALYs (251·0–280·1) in low SDI countries. In proportion terms, age-related disease burden accounts for 51·3% (95% UI 48·5–53·9) of all burden. Across the SDI, 50·0% (47·6–52·4) of all burden was age related in low SDI countries, 51·2% (48·9–53·5) in low-middle SDI countries, 52·8% (50·1–55·4) in middle SDI countries, 55·9% (52·8–59·1) in high-middle SDI countries, and 47·6% (44·2–51·1) in high SDI countries (figure 1A). Across super-regions, age-related disease burden accounts for more than half of all burden in central Europe, eastern Europe, and central Asia (56·6%, 95% UI 54·0–59·2); southeast Asia, east Asia, and Oceania (56·2%, 53·2–59·2); south Asia (54·6%, 52·0–57·2); and North Africa and the Middle East (51·3%, 48·0–54·8); and slightly less than half of all burden in the high-income super-region (46·7%, 43·3–50·3), Latin America and the Caribbean (46·4%, 44·0–48·8), and sub-Saharan Africa (41·2%, 39·4–43·1; figure 1B).

In 2017, the five countries with the lowest age-standardised age-related disease burden rate were Switzerland (104·9 DALYs [95% UI 95·7–115·5] per 1000 adults aged 25 years and older), followed by Singapore (108·3 DALYs [98·6–119·9]), South Korea (110·1 DALYs [100·7–120·4]), Japan (110·6 DALYs [101·3–121·7]), and Italy (115·2 DALYs [105·5–126·8]); whereas the five countries with the highest burden rate were Papua New Guinea (506·6 DALYs [452·3–576·1]), the Marshall Islands (396·9 DALYs [358·3–442·7]), Vanuatu (392·1 DALYs [332·0–469·3]), Afghanistan (380·2 DALYs [340·4–423·3]), and the Solomon Islands (368·0 DALYs [330·3–405·5]). Although most countries have similar rankings between age-standardised age-related and all-burden rates, countries such as Ethiopia, Nigeria, and South Africa perform better in age-related disease burden relative to all burden (eg, Nigeria ranked 78th for age-related disease burden compared with 144th in all burden), whereas countries such as China and India are performing better in all-burden rankings (eg, China ranked 75th in age-related burden and 46th in all burden; appendix).

**Figure 2:**
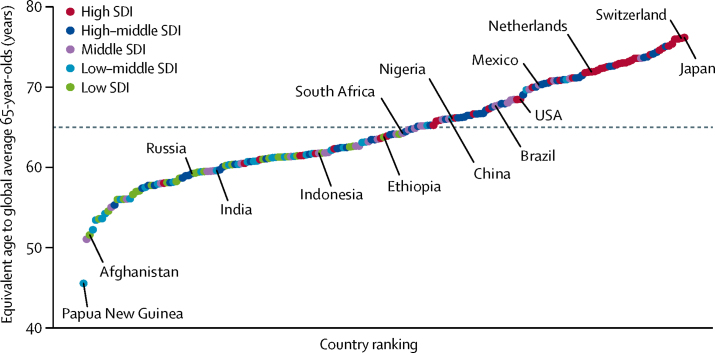
Comparing the equivalent ages to global average 65-year-olds across countries in 2017 The dashed line indicates global average 65-year-olds. Countries and territories are colour coded by their e. SDI=Socio-demographic Index.

The age-related disease burden rate for global average 65-year-olds in 2017 is 392·9 DALYs (95% UI 375·6–412·1) per 1000 adults. Comparing across countries, the equivalent ages to this reference population ranged widely, with the oldest equivalent age in Japan (76·1 years, 95% UI 75·6–76·7) and the youngest in Papua New Guinea (45·6 years, 42·6–48·2; figure 2). A simple explanation of this result is that 76-year-olds in Japan have the same age-related disease burden (or, more colloquially, are as old) as average 65-year-olds globally. The estimated equivalent ages for all countries in 2017 are listed in the appendix.

**Figure 3:**
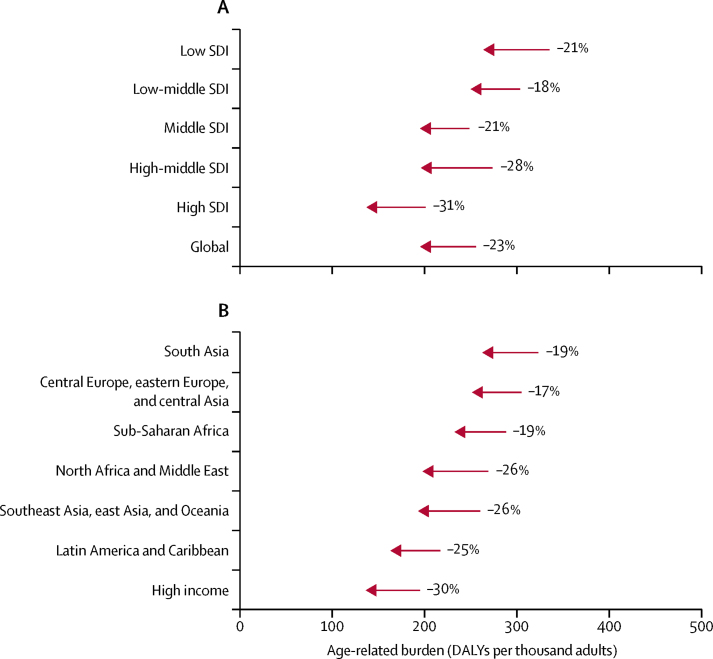
Changes in age-standardised age-related burden rate between 1990 and 2017, by SDI (A) and GBD super-region (B) Percentage change between 1990 and 2017 is presented next to the arrows indicating direction of change. DALYs=disability-adjusted life-years. GBD=Global Burden of Diseases, Injuries, and Risk Factors Study. SDI=Socio-demographic Index.

**Figure 4:**
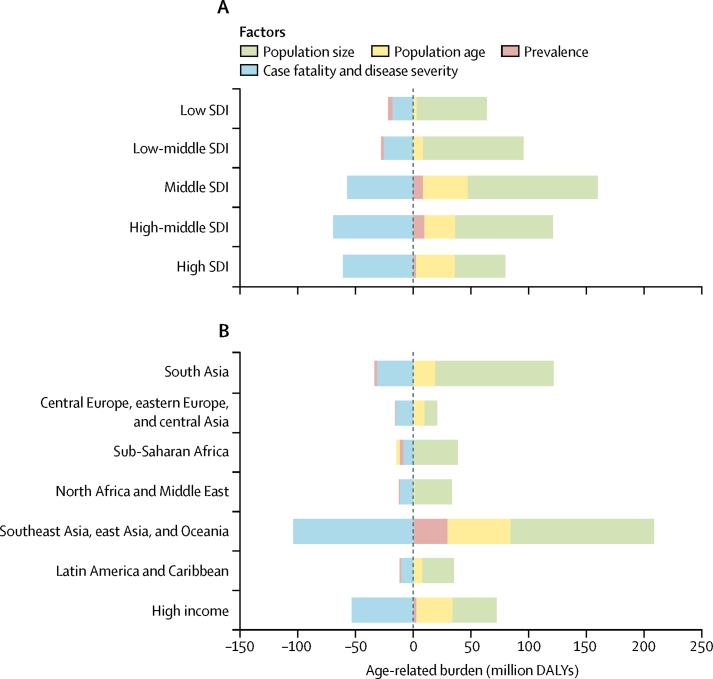
Decomposition of age-related burden between 1990 and 2017, by SDI (A) and GBD super-region (B) The dashed line marks net effect (zero change in DALYs from 1990). DALYs=disability-adjusted life-years. GBD=Global Burden of Diseases, Injuries, and Risk Factors Study. SDI=Socio-demographic Index.

Age-standardised age-related disease burden rates decreased over time across all SDI levels and regions between 1990 and 2017, with a global average of a 23·1% (95% UI 21·6 to 24·4) decline relative to the 1990 burden. We estimated the largest decline in high SDI countries (31·5%, 30·0 to 33·0) and the smallest decline in low-middle SDI countries (17·8%, 14·8 to 20·5; figure 3A); in terms of GBD super-regions, the largest decline was in the high-income super-region (30·1%, 28·6 to 31·6) and the smallest was in central Europe, eastern Europe, and central Asia (17·2%, 16·2 to 18·2; figure 3B). The decomposition analysis showed that the two largest contributors to changing age-related disease burden during this period were the increasing size of adult populations, leading to increased age-related DALYs, and the decreasing case fatality and disease severity of age-related diseases, defined as the ratio of DALYs to prevalence, leading to decreased DALYs across all SDI levels and regions (figure 4). In middle SDI, for example, change in the burden associated with increasing size of the adult population was 83·1% (82·1 to 84·2) of the absolute level of age-related disease burden in 1990, and change associated with decreasing case fatality and disease severity of age-related diseases was −42·3% (−44·9 to −39·7), compared with 28·9% (28·4 to 29·4) due to changing age distribution of the adult population and 6·4% (4·5 to 8·2) due to increasing prevalence of age-related diseases (appendix). In central Europe, eastern Europe, and central Asia and in the high-income super-region, changes in burden associated with decreasing case fatality and disease severity were greater in magnitude than were changes associated with increasing adult population size (−19·8% [95% UI −21·3 to −18·3] *vs* 13·6% [13·6 to 13·7] in central Europe, eastern Europe, and central Asia and −41·7% [−44·1 to −39·4] *vs* 29·9% [29·6 to 30·1] in the high-income super-region). Linking the results from the decomposition analysis to figure 3, large declines in age-standardised age-related disease burden rates are the product of large reductions in case fatality and disease severity of age-related diseases for most regions. Changes in population age structure, while not the main contributors to change, still account for more than 20% of the 1990 burden for some SDI levels (middle and high SDI) and some super-regions (high income; Latin America and the Caribbean; and southeast Asia, east Asia, and Oceania). Changes in prevalence of age-related diseases account for increases in age-related disease burden in high (1·9%, 0·6–3·4), high-middle (7·0%, 4·8–9·2), and middle (6·4%, 4·5–8·2) SDI countries; southeast Asia, east Asia, and Oceania (17·6%, 14·7–20·2); and the high-income super-region (2·1%, 0·8–3·8), but decreases in the rest of the SDI groups and super-regions (appendix).

**Figure 5:**
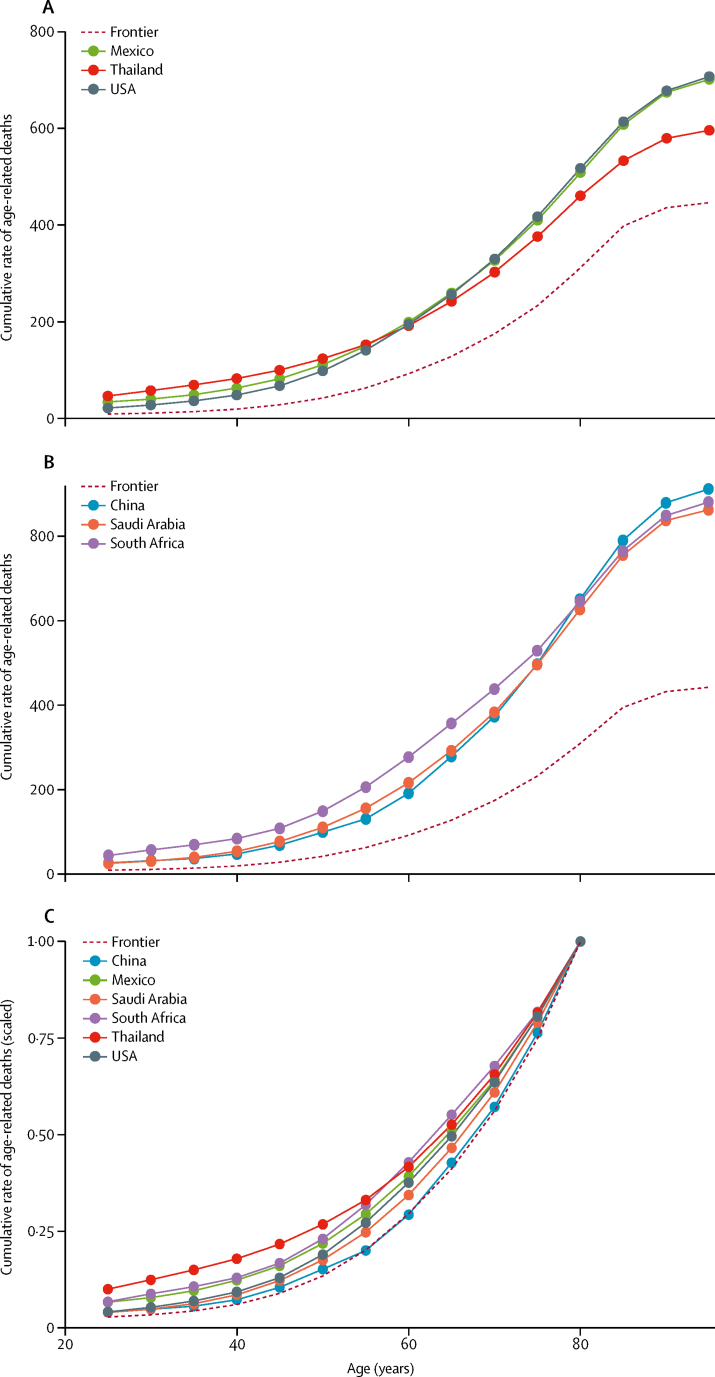
Comparison of age-standardised cumulative rates of age-related deaths in Mexico, Thailand, and the USA (A) and China, South Africa, and Saudi Arabia (B) and the frontier countries, with comparison of the scaled age-standardised cumulative rate of age-related deaths (C) Cumulative death rates in panel C are scaled to a range of 0 to 1, using country-specific cumulative death rate at age group 80–84 years (the highest observed life expectancy) as the reference point. The frontier countries are Switzerland (ages 25–64 years and 70–84 years), South Korea (65–69 years), Singapore (85–89 years), and Kuwait (90 years and older).

In 2017, Switzerland had the lowest cumulative age-standardised age-related death rates in all age groups from 25–29 years to 60–64 years. South Korea was the frontier country for 65–69 years, Switzerland again for 70–84 years, Singapore for 85–89 years, and Kuwait for 90 years and older. We grouped countries into ten categories based on similar levels of age-standardised death rates and graphed the different patterns of cumulative age-related deaths (appendix). For illustration, we selected two sets of three countries of general interest and with similar rates and compared them with one another as well as with the frontier. The first set of country comparisons comprises the USA, Mexico, and Thailand, all of which had similar levels of age-standardised death rates (figure 5A). The second set comprises China, South Africa, and Saudi Arabia (figure 5B). In both sets, despite having similar age-standardised death rates among adults, cumulative death rates accumulate earlier among younger people in some countries than others (Thailand and South Africa, respectively). Furthermore, by scaling all cumulative death rates, we can more easily compare the onset of age-related deaths across countries with different levels of age-standardised death rates among adults as well as with the frontier (figure 5C). For example, in this comparison of countries from both sets of countries, we see that, relative to each country's older age group (80–84 years), China's cumulative age-related death rate follows the patterns observed from the frontier countries the most closely, compared with its peers with similar absolute death rates (Saudi Arabia and South Africa).

## Discussion

To our knowledge, this is the first comprehensive study of age-related disease burden that captures a wide range of morbidities and mortalities associated with ageing that can be compared across countries and over time. Our findings point to the wide variation in the patterns of age-related disease burden in 2017: the highest estimated rate of age-standardised age-related disease burden is more than four times higher than the lowest rate. Despite not living as long, we find that adults in low SDI countries still experience high burden of age-related diseases. We observed decreasing trends in age-standardised age-related disease burden across all SDI levels and regions between 1990 and 2017 and identified increases in the size of the adult population and decreases in case fatality and disease severity of age-related diseases as the two main drivers of change. Findings from our equivalent age analysis show a staggering difference of 30 years between countries with the highest and lowest equivalent ages compared with global average 65-year-olds. Even among countries with similar levels of overall age-standardised age-related death burden, the patterns of accumulation of burden across ages vary widely, with some populations accruing age-related disease burden at earlier ages than others.

WHO's Global Strategy and Action Plan on Ageing and Health 2016–2020 urges countries to take action to ensure that all individuals have the opportunity to live a long and healthy life.^25^ Ongoing demographic changes, referred by some as the fourth stage of the epidemiologic transition, ^26^ suggest that the size of the older population will only increase with time. Following some of the prominent literature on ageing that proposes using age-related diseases as biomarkers for ageing,^18^, ^27^, ^28^ we identified a set of age-related diseases from a comprehensive list of causes to construct this measure, instead of relying on a small set of physical, mental, and cognitive functions. This approach allows for measurement of both the longevity and health of populations and avoids setting arbitrary age thresholds to define older populations, assessing different factors that have led to changes in longevity and health over time and capturing the onset of age-related disease burden in populations. An improved ageing metric such as ours could inform policies around, for example, how older but healthier people might experience behavioural changes towards increasing saving rates or delayed retirement, how health systems should adapt to emphasise disease prevention and early detection instead of treatment, and how families organise themselves around care of older generations.^29^ Having an ageing metric that allows countries to track age-related burden over time could help to drive policy discussions on where to allocate resources, and comparing across countries could help us to understand why certain countries, despite similar income and development level, are better at preventing age-related diseases than others.^19^

Our main finding that age-standardised age-related disease burden is decreasing over time across the globe is, in some ways, not surprising given our broader knowledge that population health is improving in most places over time. However, globally the general focus and interest have often been placed on improvements in child and maternal health instead of the ageing population. Our study finds that, in fact, the health of adult populations, even among the diseases that are associated with increasing age, is also improving. By contrast with the conventional understanding of ageing that observes changing age structure, we find that countries that are experiencing increasing median population age are also enjoying larger decreases in age-standardised age-related disease burden rates over time.^30^

Typical discussions around population ageing involve conversations about the changing size and age structures of older populations.^2^ This is partially supported by our analysis, which found that the increasing size of the adult population is one of the largest factors associated with increasing age-related burden between 1990 and 2017. However, far less attention has been placed on variation in prevalence and case fatality and disease severity of age-related diseases. While the change in the prevalence of age-related diseases accounts for a smaller proportion of change over time, our findings suggest that decreasing case fatality and disease severity of age-related diseases is associated with a large proportion of the decrease in the age-related disease burden among adults, even larger than the proportion due to changing age structure. The change in case fatality and disease severity can be explained by the change in the distribution of the sequelae of the diseases over time. For example, GBD captures the distribution of time spent in four phases of cancers: diagnosis or treatment, remission, metastatic or disseminated, and terminal.^31^ With improved diagnoses and treatment options, patients spend more time in the remission phase facing lower disease case fatality and disease severity. Furthermore, the decomposition analysis highlights that the reduction of case fatality and disease severity is smaller for some regions (such as sub-Sarahan Africa) and SDI levels (such as low and low-middle SDI) than for others, suggesting that the benefits of improved treatment might have been reaped in some parts of the world but not others.

A strength of our work is the ability to compare across countries and time. Even among high-income countries, the differences are wide: 76-year-olds in Japan have the same level of age-related disease burden as 69-year-olds in the USA. Countries experience different patterns of ageing, and our results highlight the fact that the age thresholds for identifying older populations should not be a fixed number of 60 or 65 years of age. Finally, comparing the onset in which age-related burden accumulates through age groups, we identified frontier countries as well as countries that have been relatively successful in delaying the accumulation of age-related disease burden to older ages compared with their peers with similar levels of age-related disease burden. Understanding which factors contributed to the successful delay, such as high physical activity, lower smoking rate, and better access to care, could shed light on how to reduce the projected burden of population ageing.^32^

Our study has several limitations. First, this study is based on GBD 2017 and therefore shares the limitations of the overall study, such as greater uncertainty among older age groups, locations with scarce data, and under-reported diseases.^17^, ^33^ We attempted to address this uncertainty by providing the uncertainty ranges of all estimates. Second, although the list of diseases in GBD 2017 is fairly comprehensive, we might be missing out on some that are important for ageing populations. There is not a commonly agreed-upon definition of age-related diseases. Most literature categorises broader disease groups as age related, such as neurological disorders and cancers.^20^, ^34^ However, as shown in our analysis, there are wide variations of age patterns even within these broad disease categories. Sensitivity analyses including additional diseases, such as diabetes type 2 and some musculoskeletal disorders, are presented in the appendix. Third, population ageing is a multifactorial concept,^35^ and this study captures only a subset of the phenomena. For example, although disease burden captures some of the changes in physical and mental functioning (through disability weights), the burden metric cannot replace measures such as grip strength or social participation that are commonly found in household surveys. Fourth, this study also does not consider the large burden of multimorbidity found in other studies.^35^ We probably underestimate the burden of age-related diseases because we do not take into account the interactions between co-existing diseases. Finally, although this novel approach brings new insights into the patterns and trends of population ageing, we acknowledge that this is one of many ways one can define and measure population ageing.

In conclusion, having appropriate measures for population ageing is a crucial step towards addressing the challenges and harnessing the opportunities of population ageing. From the perspective of improving the measurement of population ageing, this novel measure supports the comparison of the progression of population ageing across countries and over time without the need for additional and extensive data collection. It also facilitates the shift from thinking not just about chronological age but the health status and disease severity of ageing populations. From the perspective of providing policy recommendations for addressing challenges in population ageing, the findings from this research serve as a starting point for identifying key drivers of variation in the age-related burden and resources required for addressing the burden, and to learn from exemplar countries that have been more successful in suppressing the levels of age-related disease burden over time or delaying the accumulation of age-related disease burden to later ages.
