# Supplementary material for: Measuring population ageing: an analysis of the Global Burden of Disease Study 2017
Source: Lancet Public Health. 2019 Mar 6;4(3):e159–67. doi: 10.1016/S2468-2667(19)30019-2 (PMC6472541; doi:10.1016/S2468-2667(19)30019-2)
Supplement: Supplementary appendix [file mmc1.pdf]

THE LANCET
Public Health

Supplementary appendix

This appendix formed part of the original submission and has been peer reviewed. We post it as supplied by the authors.

Supplement to: Chang AY, Skirbekk VF, Tyrovolas S, Kassebaum NJ, Dieleman JL. Measuring population ageing: an analysis of the Global Burden of Disease Study 2017. *Lancet Public Health* 2019; **4:** e159–67.

Measuring population ageing, an analysis of the Global Burden of Disease

Supplemental materials

Angela Y. Chang, Vegard F. Skirbekk, Stefanos Tyrovoras, Nicholas J. Kassebaum, Joseph L. Dieleman

Table of Contents

1. Selection of age-related diseases 2
2. Details of methodology 8
3. Full results 12
4. Measuring the onset of ageing through cumulative age-related deaths, 2017 18
5. Sensitivity analysis 24
6. References 36

List of supplementary figures and tables

Figures

Appendix Figure 1. Flow chart of age-related disease selection 4
Appendix Figure 2. Distribution of non-age-related burden (A) and age-related burden (B) at the global level in 2017, measured in DALYs 6
Appendix Figure 3. Comparison of the timing of ageing by deciles (ten countries with similar levels of cumulative age-related death rate among adults in each decile). 18
Appendix Figure 4. 2017 global incidence rates by age for diabetes mellitus type 2, low back pain, neck pain, osteoarthritis, major depressive disorders, and anxiety disorders.. 25
Appendix Figure 5. Sensitivity analysis: Distribution of non-age-related (A) and age-related burden (B), global 2017 26
Appendix Figure 6. Sensitivity analysis: proportion of health burden that is age-related burden (age-standardised), by socio-demographic Index and region, 2017 27
Appendix Figure 7. Sensitivity analysis: change in age-related burden rate between 1990 and 2017, by socio-demographic Index and region 28
Appendix Figure 8. Sensitivity analysis: Decomposition of changes in age-related burden between 1996 and 2017, by socio-demographic Index and region.................................. 29

Tables

Appendix Table 1. GBD causes that were not classified as age-related diseases.............. 5
Appendix Table 2. Distribution of non-age-related burden and age-related burden at the global level in 2017, measured in DALYs... 7
Appendix Table 3. GBD world population age standard, proportion among adults ages 25 and above 8
Appendix Table 4. Age-standardised age-related burden per thousand adults in 1990 and 2017, share of age-related burden as proportion of all disease burden in 2017, and the equivalent age of populations compared to global 65-year-olds in 2017 12
Appendix Table 5. Data for Figure 1: age-standardised rate and proportion of all health burden among the adult population that is age-related, by SDI quintile and GBD super-region in 2017 16
Appendix Table 6. Data for Figure 3: Changes in age-standardised age-related burden rate between 1990 and 2017, by SDI and GBD super-region... 16
Appendix Table 7. Appendix Table 7. Data for Figure 4: Decomposition of age-related burden between 1990 and 2017, by SDI and GBD super-region 17
Appendix Table 8. Sensitivity analysis: distribution of non-age-related burden and age-related burden at the global level in 2017.. 26
Appendix Table 9. Sensitivity analysis: results from the decomposition analysis, comparing results from the sensitivity analysis to the original results, by SDI............... 29
Appendix Table 10. Sensitivity analysis: results from the decomposition analysis, comparing results from the sensitivity analysis to the original results, by region........... 30
Appendix Table 11. Sensitivity analysis: country-level results, 2017 age-related burden rate (per 1,000 adults), 2017 age-related burden as share of all burden, and 2017 equivalent age to global 65-year-olds 31

1. Selection of age-related diseases

Defining age-related disease

Following guidance from established ageing literature,[1–3] we define "age-related diseases" as diseases with incidence rates among the adult population increasing exponentially with age. The adult population is defined as people ages 25 and above.[4,5] To identify age-related diseases, we applied a two-step linear regression framework to the 2017 global incidence rates:

Step 1: Identify Global Burden of Disease Study 2017 (GBD) causes with incidence rate increasing with age:

$$incidence_i = \beta_0 + \beta_1\, age + \varepsilon_i \qquad (1)$$

where i represents GBD causes, age is the median age of 5-year age groups starting from 25-29 (median age 27) and ε is the error term. GBD causes with positive $\widehat{\beta_1}$ across 95% of 1,000 draw-level estimates are considered for step 2.

Step 2: Identify diseases with convex relationships between incidence and age:

$$incidence_i = \beta_0 + \beta_1\, age + \beta_2\, age^2 + \varepsilon_i \qquad (2)$$

where age^2 is the quadratic term for the age group and ε is the error term, capturing the quadratic growth in incidence rates with age. Among causes that passed step 1, we further selected diseases with positive $\widehat{\beta_2}$ across 95% of 1,000 draw-level estimates.

We analyzed incidence data for ages 25 to 80 to remove potential bias due to survival effects,[1] and then for all adults ages 25 and above (including those above age 80) and identify diseases that pass the steps described above. Finally, for a small subset of GBD causes that do not have incidence data, we relied on prevalence data and applied the same method described above.

We provide the flow chart of the number of causes from the GBD 2017 study that were include and excluded in each of the selection step. Starting with 293 most-detailed GBD cause list, we first separated 12 causes with neither incidence or prevalence data, and 60 causes that do not have incidence data (Appendix Figure 1). Among the remaining 221, we excluded 105 causes that did not have a positive relationship between incidence rates and age, and further 40 that did not have a positive term for the quadratic term, i.e., the relationship between incidence rate and age were not convex, thus excluded from the analysis. Out of the 60 causes with no incidence data, we applied the same methodology using prevalence rates, and included 16 causes that met the criteria. Overall, we identified 92 causes as age-related diseases. The causes excluded at each step are listed in Appendix Table 1.

Appendix Figure 1. Flow chart of age-related disease selection

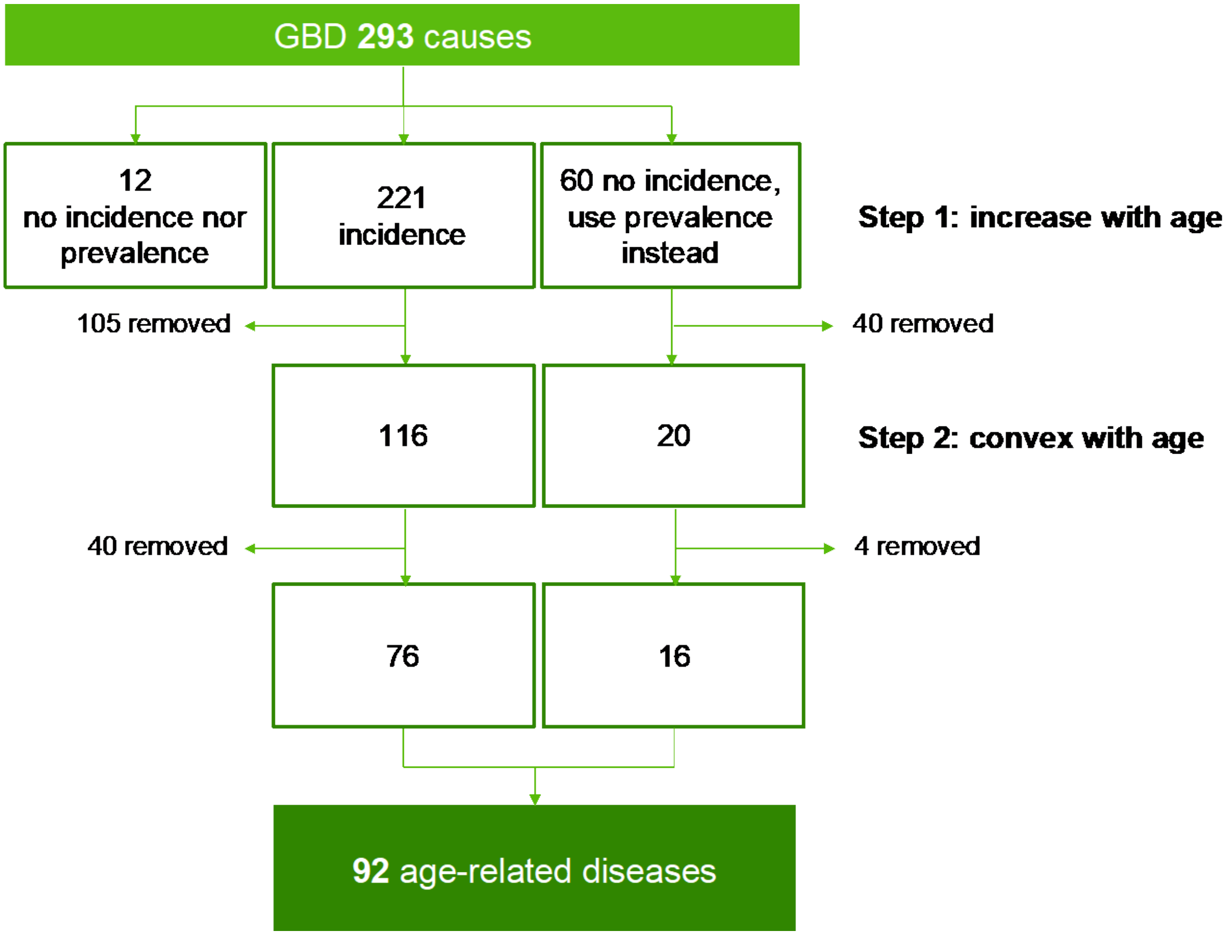

Appendix Table 1. GBD causes that were not classified as age-related diseases

Data availability and exclusion criteria	GBD causes
Causes with incidence data, excluded in Step 1	Acne vulgaris, Acute hepatitis A, Acute hepatitis B, Acute hepatitis C, Acute hepatitis E, African trypanosomiasis, Alcohol use disorders, Alopecia areata, Amphetamine use disorders, Anorexia nervosa, Anxiety disorders, Appendicitis, Benign and in situ cervical and uterine neoplasms, Bipolar disorder, Bulimia nervosa, Cannabis use disorders, Caries of deciduous teeth, Caries of permanent teeth, Chagas disease, Chlamydial infection, Chronic kidney disease due to hypertension, Cirrhosis and other chronic liver diseases due to alcohol use, Cirrhosis and other chronic liver diseases due to hepatitis B, Cirrhosis and other chronic liver diseases due to hepatitis C, Cirrhosis and other chronic liver diseases due to other causes, Cocaine use disorders, Conflict and terrorism, Cutaneous and mucocutaneous leishmaniasis, Cyclist road injuries, Cystic echinococcosis, Dengue, Diabetes mellitus type 1, Diphtheria, Dysthymia, Ebola, Ectopic pregnancy, Endometriosis, Executions and police conflict, Exposure to forces of nature, Extensively drug-resistant tuberculosis, Fire, heat, and hot substances, Foreign body in eyes, Genital herpes, Gonococcal infection, Guinea worm disease, HIV/AIDS - Drug-susceptible Tuberculosis, HIV/AIDS - Extensively drug-resistant Tuberculosis, HIV/AIDS - Multidrug-resistant Tuberculosis without extensive drug resistance, HIV/AIDS resulting in other diseases, Inflammatory bowel disease, Invasive Non-typhoidal Salmonella (iNTS), Iodine deficiency, Malaria, Maternal abortion and miscarriage, Maternal hemorrhage, Maternal hypertensive disorders, Maternal obstructed labor and uterine rupture, Maternal sepsis and other maternal infections, Measles, Meningococcal meningitis, Migraine, Motor vehicle road injuries, Motorcyclist road injuries, Multidrug-resistant tuberculosis without extensive drug resistance, Multiple sclerosis, Non-venomous animal contact, Opioid use disorders, Other exposure to mechanical forces, Other gynecological diseases, Other meningitis, Other road injuries, Otitis media, Paratyphoid fever, Pedestrian road injuries, Physical violence by firearm, Physical violence by other means, Physical violence by sharp object, Poisoning by carbon monoxide, Poisoning by other means, Polycystic ovarian syndrome, Premenstrual syndrome, Psoriasis, Pulmonary aspiration and foreign body in airway, Rheumatic heart disease, Scabies, Schizophrenia, Self-harm by other specified means, Syphilis, Tension-type headache, Testicular cancer, Trichomoniasis, Typhoid fever, Unintentional firearm injuries, Upper respiratory infections, Urinary tract infections, Urticaria, Uterine fibroids, Venomous animal contact, Viral skin diseases, Visceral leishmaniasis, Vitamin A deficiency, Whooping cough, Yellow fever, Zika virus
Causes with incidence data, excluded in Step 2	Acute glomerulonephritis, Adverse effects of medical treatment, Asthma, Atopic dermatitis, Benign prostatic hyperplasia, Cervical cancer, Chronic kidney disease due to diabetes mellitus type 1, Contact dermatitis, Diabetes mellitus type 2, Drug-susceptible tuberculosis, Edentulism and severe tooth loss, Epilepsy, Food-borne trematodiases, Gallbladder and biliary diseases, Gastritis and duodenitis, Gastroesophageal reflux disease, Genital prolapse, Gout, H influenzae type B meningitis, Inguinal, femoral, and abdominal hernia, Leprosy, Liver cancer due to hepatitis B, Liver cancer due to other causes, Low back pain, Major depressive disorder, Nasopharynx cancer, Neck pain, Osteoarthritis, Other pharynx cancer, Periodontal diseases, Protein-energy malnutrition, Pruritus, Rabies, Rheumatoid arthritis, Seborrhoeic dermatitis, Self-harm by firearm, Subarachnoid hemorrhage, Tetanus, Urolithiasis, Varicella and herpes zoster
Causes with prevalence data, excluded in Step 1	Ascariasis, Attention-deficit/hyperactivity disorder, Autism spectrum disorders, Congenital heart anomalies, Dietary iron deficiency, Down syndrome, Female infertility, G6PD deficiency, G6PD trait, Hemolytic disease and other neonatal jaundice, Hookworm disease, Idiopathic developmental intellectual disability, Klinefelter syndrome, Latent tuberculosis infection, Lymphatic filariasis, Male infertility, Neonatal encephalopathy due to birth asphyxia and trauma, Neonatal preterm birth, Neonatal sepsis and other neonatal infections, Neural tube defects, Onchocerciasis, Orofacial clefts, Other chromosomal

	abnormalities, Other congenital birth defects, Other drug use disorders, Other mental disorders, Other musculoskeletal disorders, Other neglected tropical diseases, Other oral disorders, Other sexually transmitted infections, Other unspecified infectious diseases, Schistosomiasis, Sexual violence, Sickle cell disorders, Sickle cell trait, Thalassemias, Thalassemias trait, Trichuriasis, Turner syndrome, Urogenital congenital anomalies
Causes with prevalence data, excluded in Step 2	Alcoholic cardiomyopathy, Cysticercosis, Near vision loss, Other neurological disorders
Causes with neither incidence nor prevalence data	Indirect maternal deaths, Late maternal deaths, Maternal deaths aggravated by HIV/AIDS, Aortic aneurysm, Sudden infant death syndrome, Other intestinal infectious diseases, Other maternal disorders, Other neonatal disorders, Other nutritional deficiencies, Other chronic respiratory diseases, Other digestive diseases, Other urinary diseases

Appendix Figure 2. Distribution of non-age-related burden (A) and age-related burden (B) at the global level in 2017, measured in DALYs

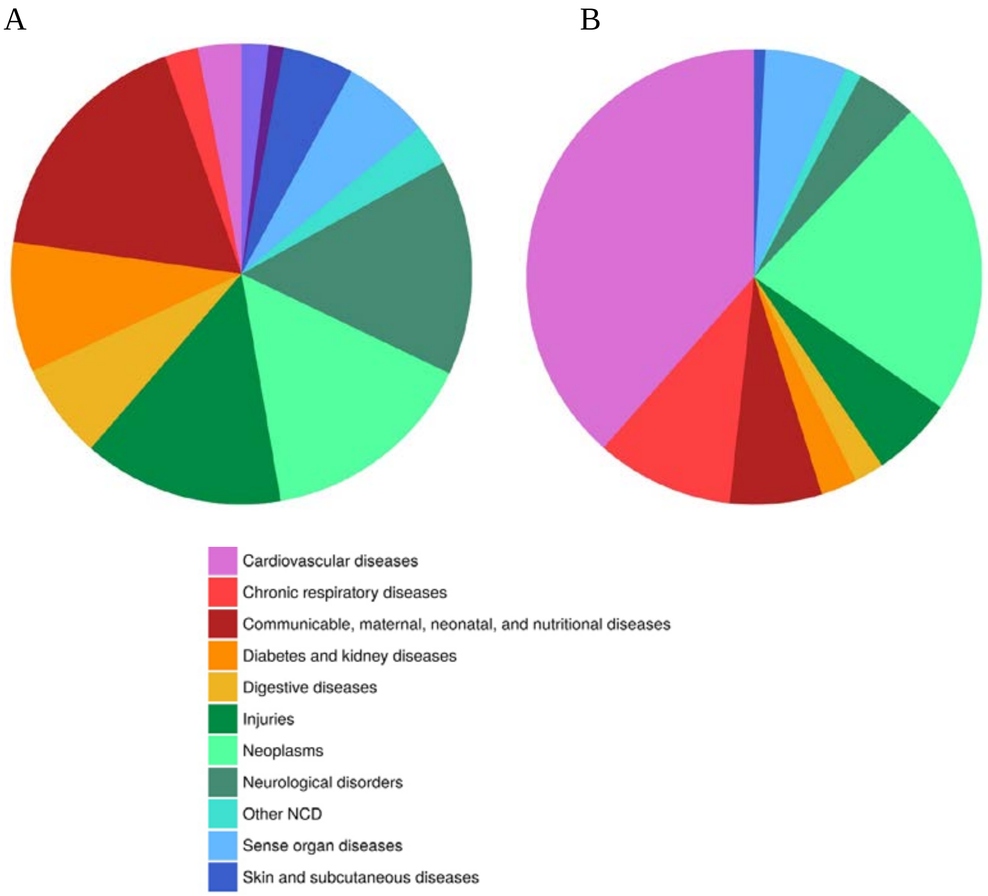

Appendix Table 2. Distribution of non-age-related burden and age-related burden at the global level in 2017, measured in DALYs

GBD category	Non-age-related burden (%)	Age-related burden (%)
Communicable, maternal, neonatal, and nutritional diseases	17.3 (15.9, 18.8)	6.5 (5.8, 7.9)
Neoplasms	2.9 (2.5, 3.4)	22.6 (21.6, 23.6)
Cardiovascular diseases	3.0 (2.7, 3.5)	38.4 (37.1, 39.7)
Chronic respiratory diseases	2.3 (2, 2.8)	9.8 (9.1, 10.4)
Digestive diseases	6.7 (6.1, 7.5)	2.1 (2, 2.2)
Neurological disorders	6.1 (4.8, 7.7)	4.3 (4.1, 4.5)
Mental and substance use disorders	15.1 (13, 16.8)	0.0 (0.0, 0.0)
Diabetes and kidney diseases	9.1 (8.6, 9.7)	2.6 (2.4, 2.7)
Other NCD	5 (4.1, 6.3)	1.1 (0.9, 1.3)
Musculoskeletal disorders	15.1 (13, 17.6)	0.0 (0.0, 0.0)
Skin and subcutaneous diseases	1.9 (1.5, 2.4)	0.8 (0.5, 1.3)
Sense organ diseases	1.0 (0.6, 1.9)	5.9 (4.3, 7.7)
Injuries	14.0 (12.6, 15.6)	5.8 (5.2, 6.5)

2. Details of methodology

I. Age standardization

Age-standardisation is an important method for comparing epidemiologic profiles of diseases across populations (geography or time) with different age structures. It was performed by multiplying the proportion of people in each age group among all adults from the GBD 2017 reference population and summing across all adults. Age-standardized populations in GBD 2017 was developed using the non-weighted means of 2017 age-specific proportional distribution from the GBD 2017 population estimates for all national locations with a population greater than five million people in 2017 to generate an updated standard population age structure.[6] The values used for the age standard are found in Appendix Table 3.

Appendix Table 3. GBD world population age standard, proportion among adults ages 25 and above

Age group	Proportion among adults ages 25 and above
25-29	14.2%
30-34	13.4%
35-39	12.4%
40-44	11.1%
45-49	10.0%
50-54	8.9%
55-59	7.8%
60-64	6.6%
65-69	5.3%
70-74	3.9%
75-79	2.9%
80-84	2.0%
85-89	1.1%
90-94	0.4%
95+	0.1%

II. Decomposition

First, we express the age-related disability-adjusted life year (DALY), $\text{DALY}_{\text{a,d,y}}$, as the product of four factors:

$$DALY_y = \sum_{a,d} pop\ size_y \cdot \frac{pop\ age\ _{a,y}}{pop\ size_y} \cdot \frac{prevalence_{a,d,y}}{pop\ age_{a,y}} \cdot \frac{DALY\ _{a,d,y}}{prevalence_{a,d,y}} \quad (3)$$

Where a is the age group, d is disease, and y is year.

For simplicity, let
R be the age-related disease DALYs by age and disease,
A be the size of the adult population A (the first term on the right-hand side)
B be the age structure of the adult population (or the mean age of the population) (second term)
C be the disease-specific prevalence rate of age-related diseases for each age group (third term), and
D be the severity of age-related diseases (fourth term).

Then, we can simplify the identity for the two time periods of interest into:

$$\text{R}_{1990} = \text{A}_{1990}\ \text{B}_{1990}\ \text{C}_{1990}\ \text{D}_{1990} \quad (4)$$

$$\text{R}_{2017} = \text{A}_{2017}\ \text{B}_{2017}\ \text{C}_{2017}\ \text{D}_{2017} \quad (5)$$

To estimate the additive contribution of A, for example, Das Gupta (1993)'s approach assumes that B, C, and D remain the same over time by applying a standardised rate of B, C, and D. The additive contribution of population size, A, on the change in age-related disease burden between 1990 and 2017 is then calculated as

$$(\text{A}_{2017} - \text{A}_{1990}) \times \left[\frac{\text{B}_{2017}\text{C}_{2017}\text{D}_{2017} + \text{B}_{1990}\text{C}_{1990}\text{D}_{1990}}{4} + \frac{\text{B}_{2017}\text{C}_{2017}\text{D}_{1990} + \text{B}_{2017}\text{C}_{1990}\text{D}_{2017} + \text{B}_{1990}\text{C}_{2017}\text{D}_{2017} + \text{B}_{1990}\text{C}_{1990}\text{D}_{2017} + \text{B}_{1990}\text{C}_{2017}\text{D}_{1990} + \text{B}_{2017}\text{C}_{1990}\text{D}_{1990}}{12}\right] \quad (6)$$

where the latter term in square brackets are the BCD-standardized rates for the two time periods. We follow the same step to estimate the contributions of B, C, and D.

III. Estimating the equivalent age of populations across countries and time, compared to average global 65-year-old in 2017

We estimated the age of populations when other countries have the same health burden as average global 65-year-olds. This approximates an age of equivalence, using global 65-year-olds as the reference group. First, since age-related burden rate is estimated for five-year age groups, we estimated the rate of age-related burden in global 65-year-olds by taking the average of the age-related burden rate of the two adjacent age groups, 60 to 64 and 65 to 69-year-olds. Second, we identified the age groups in other countries with the closest age-related burden rate to global 65-year-olds. To estimate the exact equivalent age (instead of the five-year age group), we assume a linear relationship between the age-related burden rate in two adjacent age groups.

For example, if we find that the age-related burden rate of the global 65-year-olds is the closest to the burden rate of age group 55 to 59 (median age 57) in country x, we estimate the equivalent age by the following equation:

$$\frac{Equ\ age-52}{62-52} = \frac{rate_{global\ 65}-rate_{x\ 52}}{rate_{x\ 62}-rate_{x\ 52}} \quad (7)$$

where $rate_{US\ 65}$ is the age-related burden rate for global65-year-olds, $rate_{x\ 52}$ and $rate_{x\ 62}$ are the age-related burden rates for age groups 50 to 54 and 60 to 64 (the age groups below and above age group 55 to 59) in country x. We solve for $Equ\ age$, which is the equivalent age in country x with the same level of age-related burden as global 65-year-olds.

IV. Estimating DALYs and some limitations in GBD 2017

We briefly summarize some of the basic methodologies and assumptions behind the Global Burden of Disease Study (GBD), and details can be found elsewhere.[7] The Global Burden of Disease Study 2017 spans the period from 1990 to 2017, and estimates deaths, DALYs, prevalence, incidence, and other health metrics for 195 countries and territories, for 23 age groups and both sexes. To allow meaningful comparisons between deaths and non-fatal disease outcomes as well as between diseases, the data on deaths and prevalence are summarized into a single indicator, the DALY, which is the sum of years of life lost (YLLs) and years lived with disability (YLDs). YLLs are estimated as the multiplication of counts of deaths and an 'ideal' remaining life expectancy, which is derived from the lowest empirically observed mortality rates in any population in the world greater than 5 million. YLDs are estimated as the product of prevalence of individual consequences of disease (or sequelae) times a disability weight that quantifies the relative severity of a sequela as a number between zero (representing 'full health') and 1 (representing death). Disability weights were estimated from population surveys and internet survey, described in detail elsewhere.[8]

Our study is based on GBD 2017 and therefore shares the limitations of the overall study. The limitations that are more relevant to this study include:

1. DALY estimates are influenced by the availability of data for years of life lost (YLL) and years lived with disability (YLD). Availability in the reporting of health data for older age groups and/or locations with scarce or lagged data reporting lead to wider uncertainty

intervals of the DALY estimates. Our study reflects the uncertainty by providing the uncertainty intervals (UI) for our findings.

2. As described in more detail in the most recent GBD capstone papers,[9] there is considerable heterogeneity in terms of data availability for some locations and diseases, and the study found that data availability does not consistently correlate with burden.

3. The burden of diseases with lower availability of diagnostic and/or poorer reporting systems are associated with greater uncertainty (as reflected in the uncertainty intervals).[9]

4. GBD results are a combination of data and estimation. Lags in data reporting imply that the estimates for more recent years rely more on modelling process.

3. Full results

Appendix Table 4. Age-standardised age-related burden per thousand adults in 1990 and 2017, share of age-related burden as proportion of all disease burden in 2017, and the equivalent age of populations compared to global 65-year-olds in 2017

Country	Socio-demographic Index (SDI)	1990 age-related burden rate	2017 age-related burden rate	2017 percent of all burden that is age-related burden	2017 equivalent age to global 65-year-olds	Country ranking of 2017 age-related burden rate	Country ranking of 2017 all-related burden rate
Afghanistan	Low	462.1 (292.4, 528.2)	380.2 (340.4, 423.3)	55.1 (52.2, 58.0)	51.6 (49.2, 53.5)	192	185
Albania	High-middle	215.9 (203.4, 230.3)	185.4 (161.1, 215.3)	59.6 (55.4, 63.7)	66.7 (64.9, 68.7)	76	45
Algeria	Middle	222.5 (206.3, 237.8)	161.0 (149.3, 173.6)	50.2 (46.3, 54.1)	69.7 (69.2, 70.2)	52	48
American Samoa	High-middle	273.0 (257.6, 287.7)	263.9 (242.1, 286.0)	55.1 (51.8, 58.0)	60.4 (58.6, 62.4)	158	133
Andorra	High	164.4 (148.5, 183.8)	126.1 (113.8, 140.6)	47.7 (43.4, 52.1)	73.6 (72.1, 74.9)	17	14
Angola	Low-middle	359.1 (305.7, 406.9)	253.3 (225.3, 283.9)	42.4 (39.8, 45.1)	60.4 (57.8, 62.2)	148	169
Antigua and Barbuda	High	199.5 (188.4, 212.0)	160.4 (150.8, 172.8)	48.8 (45.6, 51.8)	68.4 (67.6, 69.5)	51	54
Argentina	High-middle	233.2 (224.7, 243.0)	177.3 (161.5, 195.7)	51.5 (48.0, 55.1)	66.4 (64.8, 68.1)	67	58
Armenia	High-middle	282.4 (268.1, 296.9)	212.2 (200.2, 225.4)	57.5 (54.3, 60.9)	64.1 (63.6, 64.7)	100	81
Australia	High	188.7 (179.5, 199.7)	125.7 (112.5, 139.9)	46.0 (42.3, 50.1)	73.6 (72.3, 74.9)	16	17
Austria	High	203.1 (193.7, 214.6)	130.3 (120.0, 142.0)	47.6 (43.9, 51.4)	73.0 (71.9, 74.1)	21	22
Azerbaijan	High-middle	277.5 (263.7, 292.8)	283.0 (265.7, 303.6)	64.4 (61.0, 67.7)	58.7 (57.7, 59.8)	169	117
Bahrain	High-middle	298.5 (285.2, 313.3)	131.1 (118.5, 143.5)	42.8 (39.8, 45.9)	71.2 (69.7, 72.4)	25	41
Bangladesh	Low-middle	310.2 (287.5, 333.6)	216.4 (197.8, 236.1)	55.2 (51.9, 58.5)	62.5 (61.1, 63.8)	105	88
Barbados	High-middle	195.6 (186.9, 205.5)	159.6 (147.2, 172.9)	48.9 (45.8, 52.1)	70.5 (69.4, 71.7)	50	50
Belarus	High	286.6 (274.9, 300.3)	249.9 (234.8, 266.7)	57.5 (54.3, 60.3)	60.5 (59.6, 61.1)	143	116
Belgium	High	201.7 (192.7, 213.0)	137.1 (126.2, 150.3)	47.3 (43.7, 51.0)	71.9 (71.0, 72.8)	31	34
Belize	Middle	189.5 (177.8, 201.2)	175.0 (165.7, 185.5)	44.0 (41.9, 46.2)	68.4 (67.5, 69.3)	63	96
Benin	Low	279.3 (254.7, 303.4)	238.2 (209.1, 275.7)	45.4 (42.7, 48.3)	61.8 (58.3, 64.3)	125	151
Bermuda	High	223.3 (213.1, 234.4)	130.5 (120.4, 141.8)	50.2 (46.5, 53.8)	72.7 (71.8, 73.7)	22	12
Bhutan	Low-middle	298.2 (268.3, 337.4)	190.5 (162.7, 216.5)	51.6 (47.4, 55.3)	65.2 (62.8, 67.6)	82	80
Bolivia	Middle	282.6 (264.6, 303.7)	194.8 (165.4, 224.9)	50.0 (45.8, 53.2)	65.2 (62.5, 67.5)	85	90
Bosnia and Herzegovina	High-middle	254.0 (241.7, 268.6)	212.3 (197.0, 229.7)	58.4 (55.0, 61.8)	64.4 (63.5, 65.0)	101	73
Botswana	Middle	282.4 (254.9, 316.4)	204.1 (188.1, 225.4)	36.3 (33.1, 39.4)	64.8 (63.1, 65.8)	91	158
Brazil	Middle	247.4 (238.6, 257.9)	174.4 (165.9, 184.6)	47.6 (44.9, 50.3)	67.6 (67.3, 68.0)	61	77
Brunei	High	274.5 (260.9, 287.7)	194.6 (180.8, 210.0)	53.1 (49.9, 55.9)	65.2 (63.9, 66.4)	84	78
Bulgaria	High-middle	304.8 (290.8, 321.4)	258.6 (241.4, 276.7)	64.7 (61.5, 67.9)	59.0 (58.2, 59.8)	152	97
Burkina Faso	Low	303.9 (280.8, 329.8)	261.2 (235.8, 290.2)	46.1 (43.4, 49.0)	60.2 (57.7, 62.0)	156	159
Burundi	Low	424.8 (377.7, 472.6)	263.7 (226.9, 299.5)	41.5 (38.7, 44.5)	59.3 (56.9, 61.9)	157	180
Cambodia	Low-middle	344.6 (317.6, 370.4)	236.0 (216.7, 260.1)	49.7 (47.3, 52.1)	61.7 (60.2, 63.0)	123	130
Cameroon	Low-middle	293.8 (271.6, 315.8)	249.3 (212.5, 287.4)	40.6 (37.7, 43.4)	60.8 (57.6, 63.5)	142	174
Canada	High	180.2 (171.9, 189.8)	125.5 (116.2, 136.3)	46.0 (42.4, 50.0)	73.2 (72.3, 74.0)	15	18
Cape Verde	Low-middle	198.1 (186.5, 210.8)	168.8 (154.5, 182.6)	46.1 (43.6, 48.7)	70.9 (68.7, 71.9)	56	76
Central African Republic	Low	408.7 (354.3, 447.9)	364.6 (313.6, 418.4)	37.8 (35.1, 40.4)	53.6 (50.8, 56.0)	190	194
Chad	Low	281.7 (257.2, 309.6)	275.4 (245.8, 310.9)	45.0 (42.5, 47.7)	58.6 (56.2, 61.2)	165	173
Chile	High-middle	210.4 (201.8, 220.5)	138.1 (125.9, 153.2)	45.1 (41.5, 48.6)	71.1 (69.8, 72.6)	35	42
China	Middle	261.7 (251.2, 272.2)	183.9 (173.9, 194.9)	57.9 (54.5, 61.3)	66.0 (65.7, 66.4)	75	46
Colombia	High-middle	202.3 (193.1, 212.7)	124.4 (111.3, 137.3)	45.4 (42.8, 48.2)	73.7 (72.2, 75.3)	12	19
Comoros	Low	360.7 (324.8, 403.4)	240.3 (212.5, 272.2)	51.5 (48.4, 54.8)	61.3 (58.8, 63.4)	130	128
Congo	Low-middle	395.8 (359.0, 430.1)	287.9 (249.5, 323.8)	42.5 (40.0, 45.0)	57.9 (56.0, 60.4)	174	183
Costa Rica	High-middle	174.6 (165.9, 184.8)	145.2 (135.2, 156.9)	50.1 (47.2, 53.0)	70.8 (70.1, 71.6)	39	32
Cote d'Ivoire	Low	288.7 (262.3, 316.5)	260.7 (231.2, 294.2)	43.4 (40.4, 45.9)	59.5 (57.0, 62.2)	154	170
Croatia	High-middle	278.3 (265.3, 293.8)	183.7 (170.8, 199.6)	56.1 (52.6, 59.7)	66.1 (65.2, 67.0)	74	52
Cuba	High-middle	201.6 (193.3, 211.4)	169.4 (155.2, 185.0)	54.9 (51.2, 58.5)	66.7 (65.1, 68.3)	57	44
Cyprus	High	188.2 (178.9, 199.0)	129.9 (118.4, 142.4)	48.2 (44.4, 52.5)	72.1 (71.0, 73.1)	20	16
Czech Republic	High	327.1 (312.4, 345.2)	185.5 (169.4, 202.8)	56.7 (53.5, 59.9)	65.7 (64.6, 66.7)	77	51
Democratic Republic of the Congo	Low	291.2 (258.2, 328.2)	260.8 (227.9, 294.5)	41.8 (38.7, 44.8)	59.4 (57.1, 61.9)	155	177
Denmark	High	218.4 (209.8, 229.1)	137.6 (127.4, 149.0)	47.3 (43.6, 51.2)	71.1 (70.3, 72.0)	33	30
Djibouti	Low-middle	303.8 (249.4, 373.0)	233.5 (183.6, 297.2)	45.6 (42.2, 48.9)	61.3 (56.6, 65.2)	121	145
Dominica	High-middle	207.1 (198.1, 216.9)	198.7 (186.3, 211.7)	51.9 (49.0, 54.8)	65.1 (64.4, 66.0)	89	86
Dominican Republic	High-middle	170.8 (159.1, 184.4)	190.6 (171.0, 211.8)	48.4 (45.4, 51.4)	65.2 (64.2, 67.2)	83	91
Ecuador	High-middle	185.8 (176.7, 196.2)	147.1 (135.0, 162.7)	44.8 (42.3, 47.3)	72.6 (71.2, 73.9)	42	53

Egypt	Middle	302.1 (288.2, 320.1)	286.0 (262.7, 313.8)	57.5 (53.9, 61.4)	58.0 (56.5, 59.5)	172	142
El Salvador	Middle	191.9 (181.4, 203.2)	177.7 (155.3, 203.1)	44.0 (41.6, 46.5)	68.4 (65.9, 71.1)	68	99
Equatorial Guinea	Middle	410.4 (349.0, 484.1)	195.6 (147.4, 255.9)	33.9 (29.8, 37.9)	64.7 (60.1, 69.6)	86	163
Eritrea	Low	475.8 (393.3, 526.5)	325.7 (277.4, 374.9)	45.8 (42.7, 48.7)	56.6 (54.1, 59.1)	186	187
Estonia	High	312.7 (300.0, 326.8)	181.9 (163.2, 202.2)	51.5 (48.2, 54.6)	66.1 (64.5, 68.4)	70	65
Ethiopia	Low	373.1 (329.7, 411.1)	208.9 (192.5, 226.9)	45.9 (43.8, 48.2)	63.8 (62.7, 64.8)	98	125
Federated States of Micronesia	Middle	377.9 (340.8, 422.2)	325.1 (275.5, 371.1)	53.7 (45.7, 57.4)	55.0 (52.4, 57.7)	185	171
Fiji	High-middle	292.1 (269.6, 317.1)	267.3 (241.3, 299.2)	48.2 (45.8, 50.4)	58.1 (56.1, 61.6)	162	157
Finland	High	213.8 (204.3, 224.7)	131.5 (121.1, 144.3)	45.5 (42.1, 49.2)	72.3 (71.4, 73.2)	26	29
France	High	174.4 (165.6, 185.3)	119.3 (109.3, 130.6)	45.8 (42.1, 49.5)	76.0 (75.1, 76.9)	8	11
Gabon	Middle	305.5 (276.3, 335.3)	227.0 (203.8, 253.6)	43.5 (40.7, 45.9)	61.8 (59.7, 63.4)	114	150
Georgia	High-middle	287.9 (273.6, 303.6)	268.5 (255.7, 282.2)	61.0 (57.9, 64.0)	60.1 (59.7, 60.6)	163	118
Germany	High	211.9 (202.6, 223.0)	144.7 (131.5, 160.1)	49.7 (45.8, 53.8)	70.7 (69.1, 72.1)	38	33
Ghana	Low-middle	271.9 (242.7, 305.5)	256.4 (231.4, 282.7)	46.1 (43.1, 48.8)	60.3 (57.9, 62.1)	150	156
Greece	High-middle	180.8 (171.9, 190.8)	147.6 (137.4, 159.1)	51.8 (47.5, 56.1)	71.4 (70.6, 72.3)	44	28
Greenland	High-middle	347.4 (333.0, 362.9)	224.5 (212.0, 236.8)	53.5 (50.3, 56.6)	60.7 (60.2, 61.4)	113	105
Grenada	High-middle	279.1 (268.6, 291.6)	208.0 (195.9, 221.0)	53.1 (50.2, 55.9)	63.4 (62.5, 64.4)	96	89
Guam	High	226.8 (215.2, 238.4)	247.6 (231.0, 266.1)	57.4 (54.3, 60.2)	61.4 (58.9, 62.9)	140	113
Guatemala	Low-middle	265.5 (255.2, 276.7)	175.2 (160.1, 190.6)	43.1 (41.1, 45.0)	69.6 (68.0, 71.2)	64	101
Guinea	Low	288.5 (266.2, 309.0)	292.7 (262.5, 323.3)	46.2 (43.9, 48.5)	57.0 (55.3, 59.0)	176	179
Guinea-Bissau	Low	421.1 (375.6, 471.4)	343.4 (306.4, 386.7)	46.9 (43.9, 49.8)	54.5 (52.4, 56.2)	187	190
Guyana	Middle	303.2 (293.6, 313.5)	247.7 (225.7, 271.1)	48.3 (45.7, 50.7)	59.6 (57.8, 61.5)	141	146
Haiti	Low-middle	372.2 (344.4, 405.3)	285.5 (249.0, 325.0)	49.9 (46.8, 52.6)	57.5 (55.3, 60.7)	171	162
Honduras	Middle	209.2 (190.8, 230.5)	189.5 (164.2, 218.4)	48.3 (45.5, 51.2)	65.9 (64.0, 68.2)	80	92
Hungary	High	340.0 (325.0, 357.9)	219.6 (204.7, 237.5)	59.1 (56.0, 62.3)	61.0 (60.1, 61.8)	106	82
Iceland	High	184.5 (174.9, 195.3)	124.8 (115.2, 136.2)	47.9 (44.2, 52.0)	73.0 (72.4, 73.6)	13	10
India	Low-middle	331.2 (311.8, 351.5)	265.6 (252.9, 279.7)	54.3 (51.7, 56.8)	59.6 (59.1, 60.0)	159	138
Indonesia	Middle	262.9 (248.1, 278.0)	235.8 (220.9, 250.1)	54.3 (51.8, 56.8)	61.7 (61.0, 62.5)	122	115
Iran	High-middle	217.2 (205.3, 228.1)	164.8 (156.3, 174.7)	47.5 (44.1, 51.2)	69.0 (68.7, 69.3)	54	59
Iraq	Middle	322.7 (291.0, 357.3)	147.5 (135.7, 160.6)	41.4 (38.5, 44.3)	70.8 (69.2, 72.0)	43	69
Ireland	High	233.4 (224.2, 244.5)	131.1 (120.4, 142.9)	47.9 (43.8, 52.2)	71.9 (71.1, 72.8)	24	20
Israel	High	184.8 (174.4, 197.1)	120.2 (109.6, 133.0)	47.2 (43.6, 51.2)	74.0 (73.2, 75.6)	9	7
Italy	High	181.6 (173.0, 192.1)	115.2 (105.5, 126.8)	46.2 (42.4, 50.8)	74.8 (74.3, 75.4)	5	5
Jamaica	High-middle	173.2 (163.8, 184.9)	175.3 (157.2, 197.2)	46.3 (43.4, 49.1)	66.2 (64.6, 68.1)	66	85
Japan	High	154.9 (146.0, 165.5)	110.6 (101.3, 121.7)	47.0 (43.5, 50.4)	76.1 (75.6, 76.7)	4	2
Jordan	High-middle	237.1 (218.2, 258.7)	149.3 (136.2, 164.8)	48.7 (44.9, 53.0)	70.0 (69.0, 71.1)	45	39
Kazakhstan	High-middle	311.5 (299.7, 325.5)	266.9 (253.9, 282.9)	57.4 (54.7, 60.1)	59.7 (58.9, 60.1)	161	127
Kenya	Low-middle	252.4 (236.0, 269.3)	228.4 (214.6, 242.8)	40.0 (38.0, 42.0)	62.1 (61.4, 62.9)	117	161
Kiribati	Low-middle	386.6 (363.9, 407.5)	347.5 (311.8, 386.3)	47.8 (45.8, 49.7)	54.2 (51.3, 56.1)	188	189
Kuwait	High	171.6 (161.7, 183.7)	118.2 (107.7, 129.7)	45.9 (42.4, 49.7)	75.3 (74.6, 76.4)	6	9
Kyrgyzstan	Middle	312.8 (296.8, 329.5)	232.7 (222.1, 245.2)	55.3 (52.4, 58.1)	61.3 (60.9, 61.7)	120	106
Laos	Low-middle	405.2 (361.0, 450.1)	260.3 (227.4, 292.4)	55.1 (50.6, 58.0)	59.4 (57.3, 61.8)	153	131
Latvia	High	317.3 (304.8, 332.5)	228.2 (206.2, 248.7)	54.6 (51.5, 57.7)	61.7 (59.9, 63.4)	116	104
Lebanon	High-middle	260 (235.0, 280.4)	187.2 (174.6, 201.2)	52.6 (48.9, 56.9)	66.5 (65.4, 67.5)	79	67
Lesotho	Low-middle	324.1 (295.1, 354.3)	360.5 (308.7, 410.9)	35.6 (32.7, 38.3)	53.6 (51.0, 56.1)	189	195
Liberia	Low	277.5 (251.3, 309.1)	244.6 (216.0, 274.3)	42.3 (39.3, 45.4)	61.3 (58.1, 63.7)	138	165
Libya	Middle	225.8 (202.3, 251.0)	236.2 (210.1, 259.9)	52.8 (49.2, 56.4)	62.6 (61.2, 64.3)	124	122
Lithuania	High	284.5 (271.9, 298.7)	231.9 (217.2, 248.9)	54.7 (51.8, 57.4)	61.5 (60.6, 62.4)	119	111
Luxembourg	High	215.4 (204.4, 228.0)	136.1 (124.5, 150.6)	47.8 (43.9, 51.9)	71.8 (70.6, 72.9)	30	27
Macedonia	High-middle	271.2 (258.7, 287.1)	212.4 (197.5, 229.2)	60.9 (57.3, 64.5)	63.4 (62.5, 64.3)	102	60
Madagascar	Low	367.4 (344.3, 392.1)	314.0 (273.7, 359.8)	54.5 (51.4, 57.5)	56.0 (53.9, 58.7)	184	164
Malawi	Low	304.6 (211.6, 344.5)	240.3 (214.9, 268.2)	40.5 (37.9, 43.4)	61.3 (59.2, 63.0)	131	168
Malaysia	High-middle	260.0 (250.3, 271.0)	223.9 (208.2, 242.2)	59.4 (56.1, 63.0)	62.5 (61.4, 63.5)	111	84
Maldives	Middle	321.9 (308.8, 335.3)	133.8 (123.6, 145.2)	52.1 (48.2, 55.9)	71.0 (69.8, 72.1)	28	8
Mali	Low	339.7 (313.8, 368.7)	250.8 (222.2, 279.4)	46.8 (43.9, 50.0)	61.1 (58.7, 63.0)	145	155
Malta	High-middle	201.0 (191.2, 212.5)	140.7 (130.9, 152.6)	50.4 (46.3, 54.8)	70.9 (70.4, 71.4)	36	24
Marshall Islands	Middle	419.0 (400.6, 439.2)	396.9 (358.3, 442.7)	58.1 (55.2, 60.6)	51.0 (49.3, 53.3)	194	184
Mauritania	Low	330.9 (302.8, 359.6)	220.5 (193.9, 248.4)	51.3 (48.6, 54.2)	62.6 (60.4, 64.9)	107	112
Mauritius	High-middle	286.2 (275.6, 297.8)	182.6 (170.8, 195.9)	46.4 (43.9, 49.0)	67.1 (66.2, 68.1)	71	93
Mexico	Middle	178.3 (169.6, 188.4)	156.2 (147.7, 166.1)	43.4 (41.2, 45.5)	70.3 (69.9, 70.7)	48	71
Moldova	High-middle	317.0 (305.2, 331.0)	250.8 (239.2, 264.5)	56.2 (53.4, 59.1)	58.9 (58.4, 59.4)	146	120
Mongolia	High-middle	384.2 (368.5, 402.9)	307.4 (284.5, 330.9)	58.7 (56.1, 61.2)	55.3 (54.2, 56.6)	181	149
Montenegro	High-middle	242.1 (229.5, 257.1)	223.0 (206.0, 243.1)	60.6 (57.0, 64.2)	62.3 (61.1, 63.4)	110	79
Morocco	Low-middle	272.4 (255.6, 290.6)	222.9 (195.4, 252.9)	54.2 (49.8, 58.2)	63.1 (61.0, 65.2)	109	103
Mozambique	Low	336.6 (308.8, 363.0)	291.4 (255.8, 328.6)	37.3 (34.1, 39.8)	57.0 (55.2, 59.3)	175	191
Myanmar	Low-middle	351.7 (307.5, 392.2)	239.6 (216.0, 262.5)	49.7 (47.6, 51.9)	61.6 (60.3, 63.0)	129	134
Namibia	Middle	327.8 (303.2, 351.9)	224.1 (201.9, 251.1)	38.2 (34.6, 41.3)	62.6 (60.7, 64.4)	112	167
Nepal	Low-middle	324.4 (288.4, 362.8)	241.9 (214.7, 264.5)	53.1 (48.6, 56.3)	60.7 (59.1, 62.6)	135	124
Netherlands	High	189.6 (181.5, 198.9)	133.8 (123.6, 144.4)	48.1 (44.1, 52.7)	71.9 (71.1, 72.7)	27	23
New Zealand	High	211.6 (201.7, 224.1)	138.0 (126.8, 152.0)	47.5 (43.9, 51.6)	72.5 (71.6, 73.3)	34	31
Nicaragua	Middle	167.3 (154.9, 180.4)	134.1 (120.8, 148.2)	44.5 (41.9, 47.1)	73.6 (72.0, 75.1)	29	38

Niger	Low	303.0 (276.3, 331.5)	241.8 (214.4, 273.0)	45.6 (42.0, 48.7)	61.4 (58.7, 63.7)	134	153
Nigeria	Low-middle	230.5 (189.4, 285.1)	186.1 (150.5, 242.0)	36.8 (33.4, 40.6)	66.1 (61.7, 69.5)	78	144
North Korea	Middle	248.6 (218.6, 276.9)	256.2 (229.2, 282.1)	60.8 (57.1, 64.1)	59.5 (58.1, 61.2)	149	108
Northern Mariana Islands	High	218.0 (197.9, 248.4)	190.0 (173.6, 208.7)	52.2 (49.0, 55.6)	66.6 (63.7, 68.0)	81	75
Norway	High	194.7 (185.9, 204.8)	125.2 (116.2, 135.9)	45.7 (42.0, 49.7)	72.9 (72.4, 73.4)	14	21
Oman	High-middle	264.8 (230.1, 304.6)	174.5 (151.8, 197.9)	49.8 (46.6, 53.2)	66.0 (63.5, 68.3)	62	62
Pakistan	Low-middle	294.0 (274.9, 311.4)	298.3 (260.9, 341.1)	57.4 (54.1, 60.3)	56.1 (54.3, 58.1)	177	148
Palestine	Middle	251.0 (220.9, 282.7)	183.5 (170.8, 197.4)	51.0 (47.1, 54.8)	65.1 (64.7, 65.9)	73	72
Panama	High-middle	162.4 (153.0, 173.1)	128.5 (118.1, 139.9)	45.2 (42.7, 47.6)	74.1 (73.2, 75.0)	18	26
Papua New Guinea	Low-middle	569.1 (511.2, 632.8)	506.6 (452.3, 576.1)	58.5 (55.9, 61.3)	45.6 (42.6, 48.2)	195	193
Paraguay	Middle	184.0 (171.5, 197.5)	167.4 (147.0, 191.1)	46.9 (43.8, 50.2)	67.8 (65.3, 70.2)	55	68
Peru	High-middle	171.0 (158.3, 184.2)	123.8 (108.2, 139.5)	45.9 (42.7, 49.0)	74.3 (72.4, 76.5)	11	15
Philippines	Middle	243.3 (228.8, 257.5)	276.2 (247.7, 306.9)	56.3 (53.5, 59.0)	59.5 (57.5, 61.5)	166	137
Poland	High	309.0 (295.5, 325.1)	198.0 (183.7, 214.7)	56.7 (53.7, 59.7)	63.9 (63.1, 64.8)	88	61
Portugal	High-middle	216.7 (207.4, 228.1)	130.7 (121.0, 141.5)	46.3 (42.3, 50.6)	73.7 (72.8, 74.6)	23	25
Puerto Rico	High	183.1 (175.3, 192.7)	128.9 (119.8, 138.8)	40.3 (36.9, 43.1)	74.6 (73.6, 75.1)	19	47
Qatar	High-middle	274.2 (246.3, 301.2)	137.5 (119.5, 156.9)	44.8 (41.6, 48.5)	70.3 (68.3, 73.0)	32	40
Romania	High-middle	313.8 (299.2, 330.8)	238.4 (222.9, 255.5)	60.1 (57.2, 63.3)	61.0 (60.1, 62.0)	128	95
Russian Federation	High-middle	308.9 (296.4, 322.8)	266.4 (253.7, 281.2)	54.1 (51.9, 56.3)	59.2 (58.9, 59.5)	160	140
Rwanda	Low	382.0 (349.9, 415.7)	203.5 (181.3, 227.3)	42.2 (39.8, 44.9)	64.1 (62.3, 65.8)	90	135
Saint Lucia	High-middle	243.5 (234.5, 253.3)	177.7 (166.4, 190.3)	48.7 (45.9, 51.4)	67.5 (66.5, 68.5)	69	74
Saint Vincent and the Grenadines	High-middle	224.0 (214.0, 234.9)	206.8 (194.5, 219.2)	48.8 (46.1, 51.3)	64.8 (63.9, 65.3)	95	110
Samoa	Middle	259.1 (230.9, 289.2)	238.2 (213.0, 260.3)	55.1 (51.2, 58.2)	61.8 (60.5, 63.4)	126	114
Sao Tome and Principe	Low-middle	248.4 (228.2, 270.1)	257.7 (231.1, 284.9)	54.2 (51.4, 56.9)	60.7 (58.2, 62.5)	151	132
Saudi Arabia	High-middle	203.0 (177.0, 233.9)	183.2 (168.1, 203.3)	51.0 (47.6, 54.7)	66.3 (64.7, 67.3)	72	70
Senegal	Low	282.7 (260.6, 307.3)	242.1 (219.1, 269.0)	48.7 (46.3, 51.6)	61.0 (58.8, 62.7)	136	141
Serbia	High-middle	296.1 (283.2, 311.2)	245.0 (229.0, 261.9)	62.1 (58.8, 65.4)	61.6 (60.9, 62.4)	139	94
Seychelles	High-middle	288.2 (276.9, 301.1)	231.3 (217.9, 244.2)	57.3 (54.3, 60.1)	62.4 (61.7, 63.2)	118	100
Sierra Leone	Low	308.6 (275.3, 342.6)	284.9 (251.4, 322.7)	46.4 (43.2, 49.5)	58.1 (55.7, 61.2)	170	172
Singapore	High	205.5 (196.4, 216.2)	108.3 (98.6, 119.9)	49.7 (45.9, 53.7)	76.0 (75.1, 76.9)	2	1
Slovakia	High	326.7 (311.9, 344.4)	208.8 (193.7, 227.9)	59.1 (55.9, 62.4)	63.6 (62.8, 64.5)	97	66
Slovenia	High	245.7 (231.1, 263.0)	154.4 (139.7, 172.4)	52.0 (48.9, 55.2)	70.0 (68.9, 71.0)	47	37
Solomon Islands	Low-middle	439.4 (392.9, 493.1)	368.0 (330.3, 405.5)	58.4 (55.6, 60.9)	53.4 (51.1, 55.1)	191	178
Somalia	Low	375.2 (266.9, 476.2)	311.4 (255.2, 382.6)	44.8 (42.1, 47.5)	56.0 (52.7, 59.6)	182	186
South Africa	Middle	240.5 (226.7, 254.1)	204.2 (194.1, 215.8)	32.7 (30.9, 34.3)	64.2 (63.7, 64.8)	92	176
South Korea	High	240.5 (230.3, 252.4)	110.1 (100.7, 120.4)	43.6 (40.3, 46.7)	75.1 (74.4, 75.8)	3	6
South Sudan	Low	327.3 (242.8, 424.7)	278.3 (227.9, 349.4)	41.8 (37.1, 45.6)	57.7 (54.3, 62.6)	167	182
Spain	High-middle	178.7 (170.3, 189.6)	119.2 (110.1, 130.0)	47.9 (44.0, 52.1)	75.1 (74.4, 75.8)	7	4
Sri Lanka	High-middle	229.7 (219.1, 241.6)	151.0 (132.3, 173.0)	46.5 (43.6, 49.3)	70.4 (67.8, 72.6)	46	49
Sudan	Low-middle	329.9 (291.2, 368.7)	240.9 (205.2, 278.9)	53.9 (49.7, 57.9)	61.2 (58.1, 64.2)	133	123
Suriname	High-middle	231.8 (219.0, 245.6)	206.1 (187.2, 224.3)	48.8 (46.2, 51.2)	64.1 (62.3, 66.0)	94	107
Swaziland	Middle	334.4 (304.1, 365.2)	306.7 (263.8, 355.7)	36.9 (34.2, 39.4)	56.0 (53.3, 59.0)	180	192
Sweden	High	178.6 (170.0, 188.7)	122.1 (112.4, 133.3)	46.3 (42.8, 50.2)	73.3 (72.6, 74.2)	10	13
Switzerland	High	169.7 (159.9, 181.7)	104.9 (95.7, 115.5)	43.0 (39.3, 47.2)	76.1 (75.4, 76.7)	1	3
Syria	Middle	284.3 (264.4, 306.1)	213.1 (186.0, 240.1)	44.0 (39.9, 48.4)	63.5 (61.2, 65.6)	103	136
Taiwan	High	195.5 (187.0, 204.8)	146.3 (136.0, 156.8)	49.7 (46.7, 52.6)	72.4 (71.7, 73.1)	40	35
Tajikistan	Middle	261.0 (248.3, 274.8)	251.9 (231.7, 272.5)	59.5 (56.1, 62.6)	60.5 (58.9, 61.6)	147	109
Tanzania	Low-middle	271.6 (224.7, 300.0)	212.1 (193.1, 233.5)	43.0 (40.6, 45.5)	63.1 (61.5, 64.6)	99	139
Thailand	High-middle	211.1 (199.7, 222.7)	146.4 (132.6, 160.5)	44.3 (41.6, 46.8)	72.6 (71.4, 74.2)	41	55
The Bahamas	High	237.1 (228.0, 247.3)	205.8 (190.4, 223.6)	50.3 (47.7, 52.8)	65.9 (63.5, 67.0)	93	102
The Gambia	Low	296.9 (260.6, 338.3)	272.6 (245.2, 304.8)	46.9 (44.3, 49.7)	58.2 (56.4, 60.5)	164	166
Timor-Leste	Low-middle	289.8 (257.8, 319.2)	238.3 (203.0, 264.2)	53.9 (46.9, 57.1)	61.3 (59.0, 63.2)	127	119
Togo	Low	286.8 (258.2, 311.5)	250.7 (219.8, 286.8)	44.2 (41.3, 47.0)	60.3 (57.2, 62.5)	144	160
Tonga	Middle	245.4 (231.2, 258.8)	227.9 (205.7, 252.8)	49.3 (46.7, 51.9)	63.2 (61.4, 64.8)	115	126
Trinidad and Tobago	High	250.1 (241.2, 260.3)	175.2 (150.0, 205.1)	44.9 (41.9, 47.7)	67.3 (64.6, 70.6)	65	87
Tunisia	Middle	208.9 (196.5, 221.8)	170.4 (144.5, 197.5)	51.4 (47.0, 55.9)	68.0 (65.6, 70.5)	59	56
Turkey	High-middle	257.2 (243.6, 270.7)	157.7 (144.6, 169.7)	51.1 (47.3, 55.3)	67.9 (66.7, 69.2)	49	43
Turkmenistan	High-middle	318.9 (307.7, 331.2)	287.6 (269.2, 307.7)	61.1 (58.2, 63.9)	57.7 (56.8, 58.6)	173	129
Uganda	Low	264.4 (231.7, 296.7)	222.3 (197.1, 247.0)	41.8 (39.1, 44.6)	62.5 (60.9, 64.5)	108	154
Ukraine	High-middle	304.4 (292.2, 318.4)	301.4 (284.0, 318.7)	57.0 (54.3, 59.6)	57.4 (56.6, 58.1)	179	152
United Arab Emirates	High	257.1 (226.5, 289.5)	240.4 (206.9, 277.5)	53.9 (49.5, 57.5)	58.0 (56.1, 61.6)	132	121
United Kingdom	High	217.9 (208.9, 228.5)	143.3 (134.1, 154.0)	48.7 (44.9, 52.7)	70.8 (70.5, 71.1)	37	36
United States	High	207.5 (197.7, 218.2)	161.5 (152.0, 171.4)	46.0 (43.0, 49.2)	68.5 (68.0, 68.9)	53	63
Uruguay	High-middle	231 (222.5, 241.2.0)	173.8 (157.8, 192.2)	51.0 (47.4, 54.4)	66.6 (65.0, 68.3)	60	57
Uzbekistan	High-middle	263.1 (252.8, 275.3)	312.6 (285.5, 340.8)	62.8 (59.4, 65.7)	57.8 (56.6, 59.0)	183	143

Vanuatu	Low-middle	431.5 (364.8, 514.0)	392.1 (332.0, 469.3)	59.0 (54.9, 62.1)	52.2 (47.2, 55.5)	193	181
Venezuela	High-middle	210.5 (201.7, 220.6)	169.6 (152.1, 192.0)	48.1 (45.5, 50.5)	67.9 (65.7, 70.2)	58	64
Vietnam	Middle	239.8 (216.7, 259.1)	197.7 (180.7, 219.9)	52.8 (50.0, 55.7)	64.1 (62.1, 65.5)	87	83
Virgin Islands, U.S.	High	221.2 (209.0, 235.5)	213.2 (185.7, 233.9)	53.0 (49.8, 55.9)	62.3 (61.0, 65.4)	104	98
Yemen	Low-middle	358.8 (269.0, 448.9)	280.6 (229.5, 342.2)	53.8 (49.3, 57.6)	58.1 (54.5, 61.5)	168	147
Zambia	Low-middle	337.9 (297.6, 367.8)	243.7 (218.5, 268.7)	39.6 (37.2, 42.1)	61.2 (59.1, 63.0)	137	175
Zimbabwe	Low-middle	249.4 (227.9, 269.2)	300.6 (268.4, 338.6)	42.0 (39.5, 44.3)	56.0 (54.5, 58.4)	178	188

Appendix Table 5. Data for Figure 1: age-standardised rate and proportion of all health burden among the adult population that is age-related, by SDI quintile and GBD super-region in 2017

	Location	Age-related burden rate (per 1,000 adults)	Non-age-related burden rate (per 1,000 adults)	Proportion of age-related burden among all burden (%)
	Global	196.5 (186.8, 207.5)	187.1 (160.6, 218.6)	51.3 (48.5, 53.9)
SDI	Low SDI	265.9 (251.0, 280.1)	265.3 (234.8, 300.7)	50.0 (47.6, 52.4)
	Low-middle SDI	249.8 (237.5, 262.5)	237.6 (209.0, 271.9)	51.2 (48.9, 53.5)
	Middle SDI	196.9 (186.9, 207.7)	176.2 (151.1, 205.9)	52.8 (50.1, 55.4)
	High-middle SDI	197.7 (188.1, 209.6)	156.6 (130.6, 185.8)	55.9 (52.8, 59.1)
	High SDI	137.8 (128.9, 148.3)	152.2 (124.2, 184.4)	47.6 (44.2, 51.1)
GBD super-region	High-income	136.4 (127.6, 146.3)	155.9 (127.5, 188.6)	46.7 (43.4, 50.3)
	Latin America and Caribbean	163.1 (154.6, 172.9)	188.6 (162.7, 219.0)	46.4 (44.0, 48.8)
	Southeast Asia, East Asia, and Oceania	192.8 (183.3, 203.7)	150.7 (127.0, 178.6)	56.2 (53.2, 59.2)
	North Africa and Middle East	198.6 (187.5, 209.7)	188.9 (158.2, 225.3)	51.3 (48.0, 54.8)
	Sub-Saharan Africa	232.9 (217.2, 248.9)	331.8 (300.2, 369.7)	41.2 (39.4, 43.1)
	Central Europe, Eastern Europe, and Central Asia	252.4 (239.7, 266.6)	193.7 (166.1, 226.2)	56.6 (54.0, 59.2)
	South Asia	263.6 (251.2, 277.4)	219.5 (191.2, 251.5)	54.6 (52.0, 57.2)

Appendix Table 6. Data for Figure 3: Changes in age-standardised age-related burden rate between 1990 and 2017, by SDI and GBD super-region

	Location	1990 Age-related burden rate (per 1,000 adults)	2017 Age-related burden rate (per 1,000 adults)	Percentage change 1990-2017 (%)
	Global	255.3 (244.3, 267.2)	196.5 (186.8, 207.5)	-23.1 (-24.4, -21.6)
SDI	Low SDI	335.2 (310.1, 359.1)	265.9 (251.0, 280.1)	-20.9 (-24.7, -15.6)
	Low-middle SDI	303.4 (288.6, 319.8)	249.8 (237.5, 262.5)	-17.8 (-20.5, -14.8)
	Middle SDI	250.1 (239.6, 260.9)	196.9 (186.9, 207.7)	-21.3 (-23.3, -19.2)
	High-middle SDI	274.3 (263.6, 285.3)	197.7 (188.1, 209.6)	-27.8 (-29.7, -26.0)
	High SDI	201.1 (191.7, 212.1)	137.8 (128.9, 148.3)	-31.5 (-33.0, -30.0)
GBD super-region	High-income	195.1 (185.9, 205.9)	136.4 (127.6, 146.3)	-30.1 (-31.6, -28.6)
	Latin America and Caribbean	217.0 (208.2, 227.1)	163.1 (154.6, 172.9)	-24.8 (-26.0, -23.5)
	Southeast Asia, East Asia, and Oceania	260.8 (250.5, 271.0)	192.8 (183.3, 203.7)	-26.0 (-28.3, -23.9)
	North Africa and Middle East	269.9 (255.1, 282.5)	198.6 (187.5, 209.7)	-26.4 (-29.1, -22.7)
	Sub-Saharan Africa	288.1 (270.8, 308.4)	232.9 (217.2, 248.9)	-19.3 (-23.5, -14.4)

	Central Europe, Eastern Europe, and Central Asia	305.0 (292.7, 319.7)	252.4 (239.7, 266.6)	-17.2 (-18.2, -16.2)
	South Asia	324.3 (306.1, 344.4)	263.6 (251.2, 277.4)	-18.7 (-21.8, -15.3)

Appendix Table 7. Appendix Table 7. Data for Figure 4: Decomposition of age-related burden between 1990 and 2017, by SDI and GBD super-region

	Location	Population size (%)	Population age (%)	Prevalence (%)	Severity (%)
SDI	High SDI	31.2 (30.9, 31.4)	24.0 (23.7, 24.2)	1.9 (0.6, 3.4)	-43.3 (-45.6, -40.8)
	High-middle SDI	59.6 (58.8, 60.3)	18.8 (18.5, 19.1)	7.0 (4.8, 9.2)	-48.7 (-51.7, -46.2)
	Middle SDI	83.1 (82.1, 84.2)	28.9 (28.4, 29.4)	6.4 (4.5, 8.2)	-42.3 (-44.9, -39.7)
	Low-middle SDI	88.5 (87.0, 90.0)	9.0 (8.8, 9.3)	-2.3 (-3.8, -0.6)	-25.9 (-29.9, -22.0)
	Low SDI	95.7 (93.4, 99.3)	5.2 (4.9, 5.5)	-5.7 (-7.7, -3.5)	-28.2 (-33.9, -19.9)
GBD super-region	High-income	29.9 (29.6, 30.1)	24.3 (24, 24.5)	2.1 (0.8, 3.8)	-41.7 (-44.1, -39.4)
	Latin America and Caribbean	95.7 (95.0, 96.4)	28.1 (27.7, 28.4)	-3.8 (-5.0, -2.6)	-38.1 (-40.2, -35.8)
	Southeast Asia, East Asia, and Oceania	73.9 (72.8, 74.9)	32.3 (31.8, 32.9)	17.6 (14.7, 20.2)	-61.8 (-65.3, -58.6)
	North Africa and Middle East	120.6 (118.6, 123.7)	4.2 (4.1, 4.3)	-3.2 (-5.4, -0.6)	-43.8 (-48.5, -38.0)
	Sub-Saharan Africa	105.6 (102.8, 108.9)	-7.1 (-7.5, -6.8)	-8.9 (-11.4, -6.3)	-24.0 (-30.0, -17.1)
	Central Europe, Eastern Europe, and Central Asia	13.6 (13.6, 13.7)	12.3 (12.2, 12.5)	-0.2 (-1.2, 1.0)	-19.8 (-21.3, -18.3)
	South Asia	94.2 (92.4, 96.2)	17.5 (17.0, 18.1)	-1.9 (-3.7, 0.0)	-29.2 (-34.1, -23.7)

The percentages are the change in DALYs associated with each factor compared to 1990 age-related DALYs.

4. Measuring the onset of ageing through cumulative age-related deaths, 2017

For each five-year age group starting at age 25, we estimated the cumulative, age-standardised age-related death rates. Countries with the lowest cumulative age-related death rate for each age group in 2017 were identified and set as frontiers: Switzerland (ages 25 to 65 and 70 to 85), South Korea (65 to 70), Singapore (85 to 90), and Kuwait (90 and older). We grouped countries into 10 categories based on similar levels of age-standardised death rates and graphed the different patterns of cumulative age-related deaths. To facilitate comparison of the ageing patterns across countries and to the frontier countries, we further scaled all cumulative death rates to a range of 0 to 1, using country-specific cumulative death rate at age group 80 to 84 (the highest observed life expectancy) as the reference point.

Appendix Figure 3. Comparison of the timing of ageing by deciles (ten countries with similar levels of cumulative age-related death rate among adults in each decile).

The first decile includes countries with the lowest age-standardised age-related death rates among adults, and the tenth decile includes countries with the highest rates. The graphs on the left are in the original unit, and the graphs on the right are in the scaled form (ranging 0 to 1).

1st decile (ten countries with the lowest age-standardised age-related death rate)

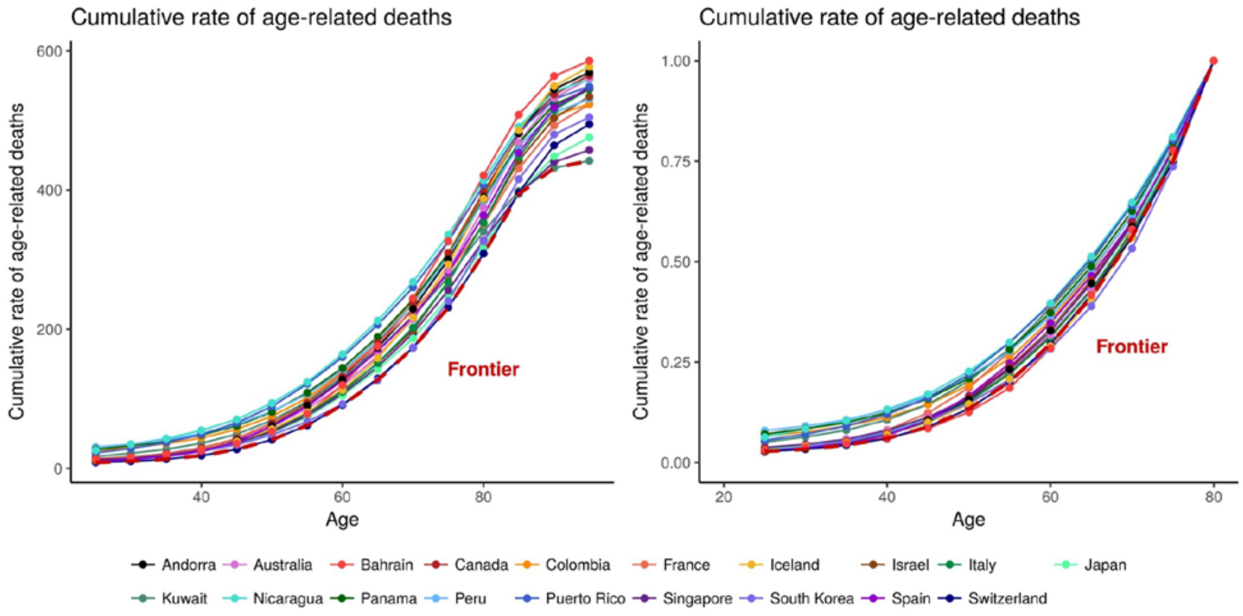

2nd decile

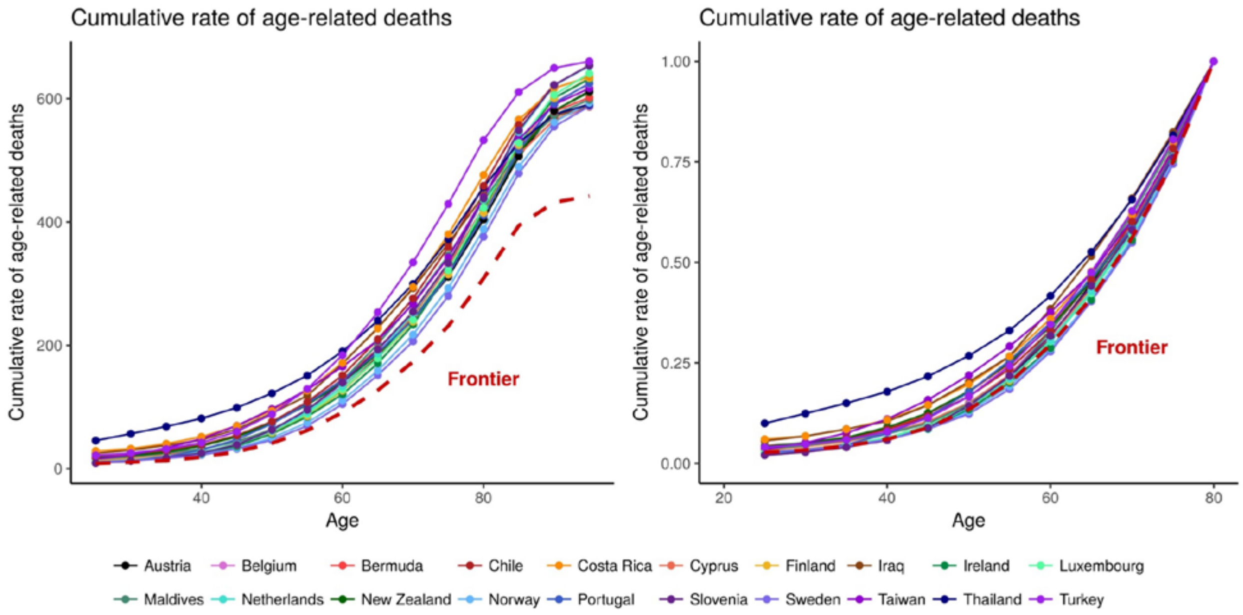

3rd decile

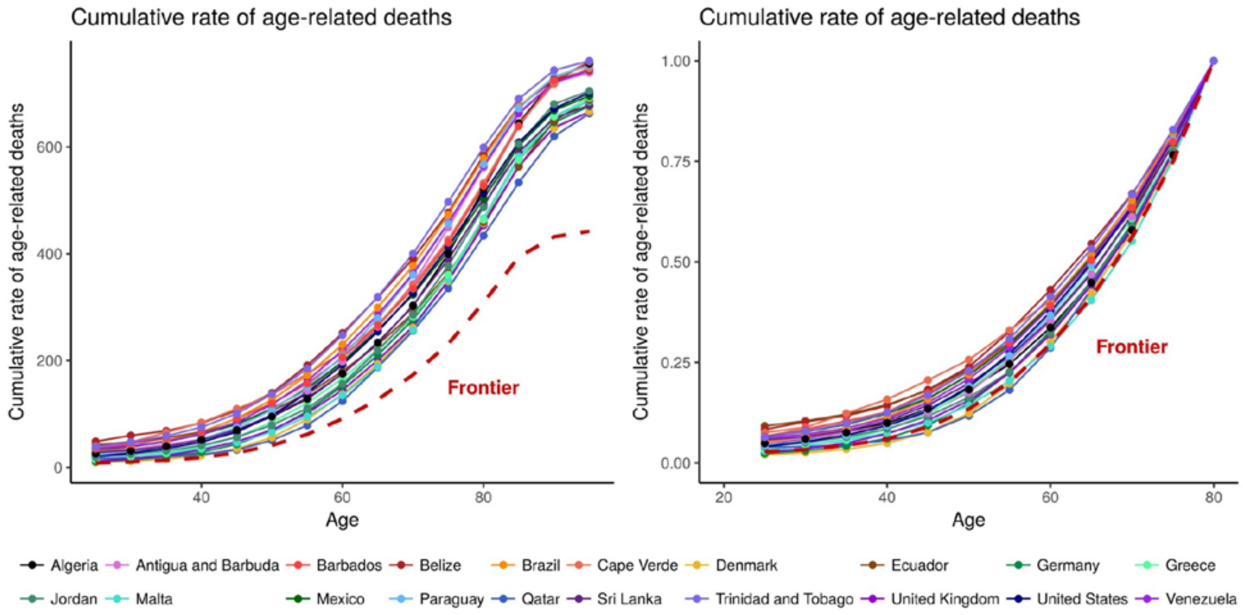

4th decile

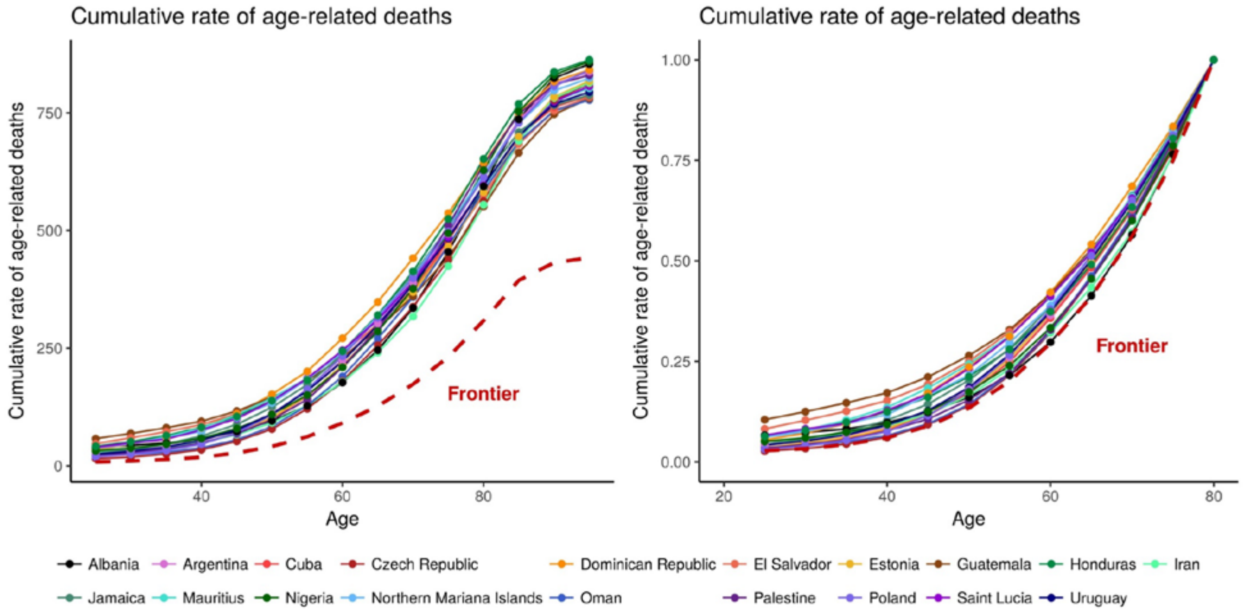

5th decile

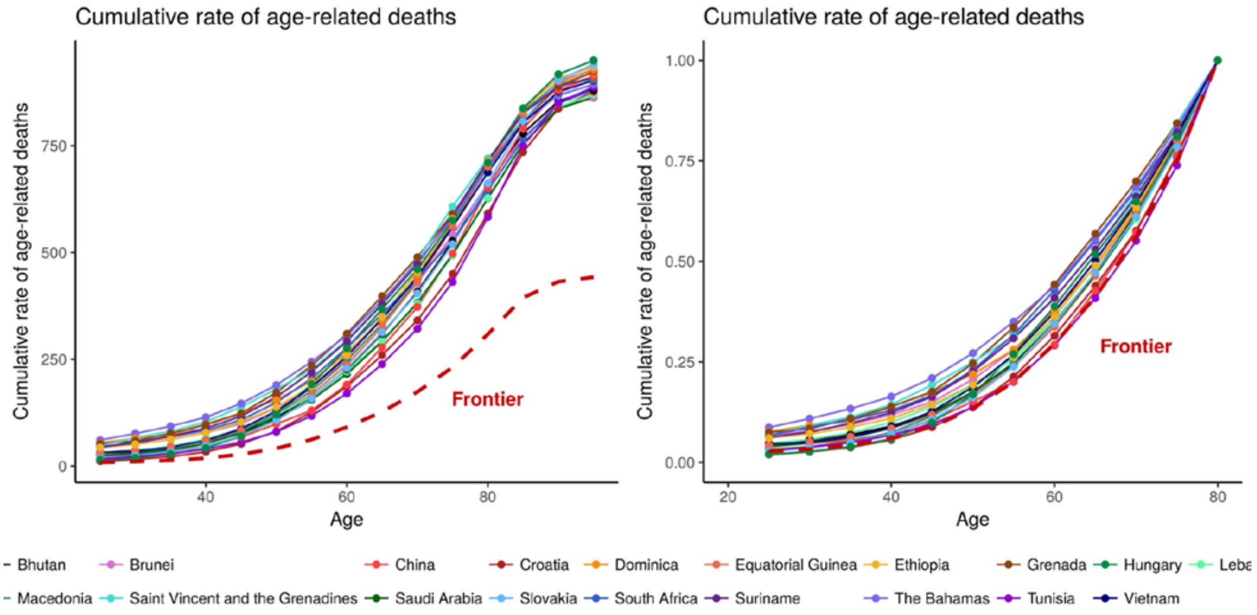

6th decile

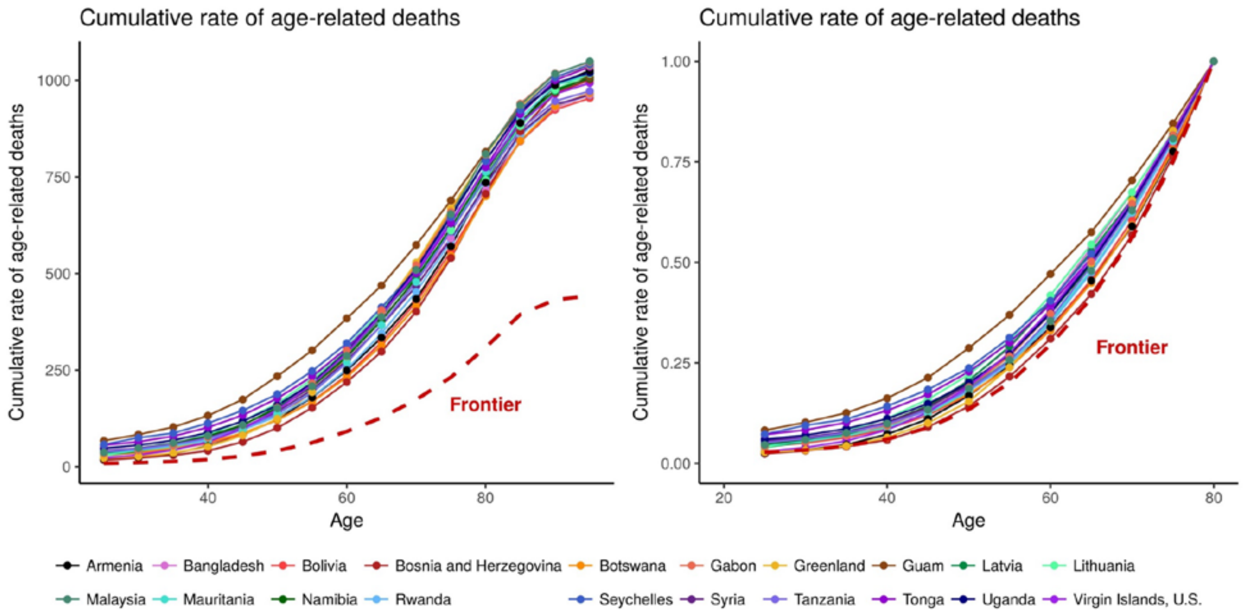

7th decile

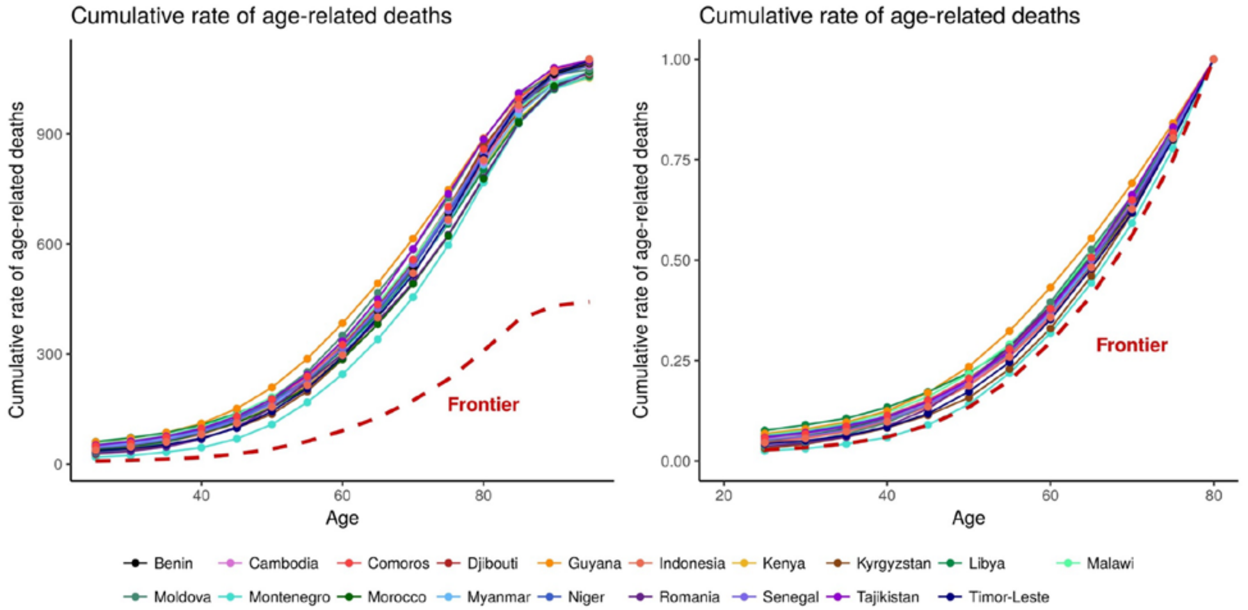

8th decile

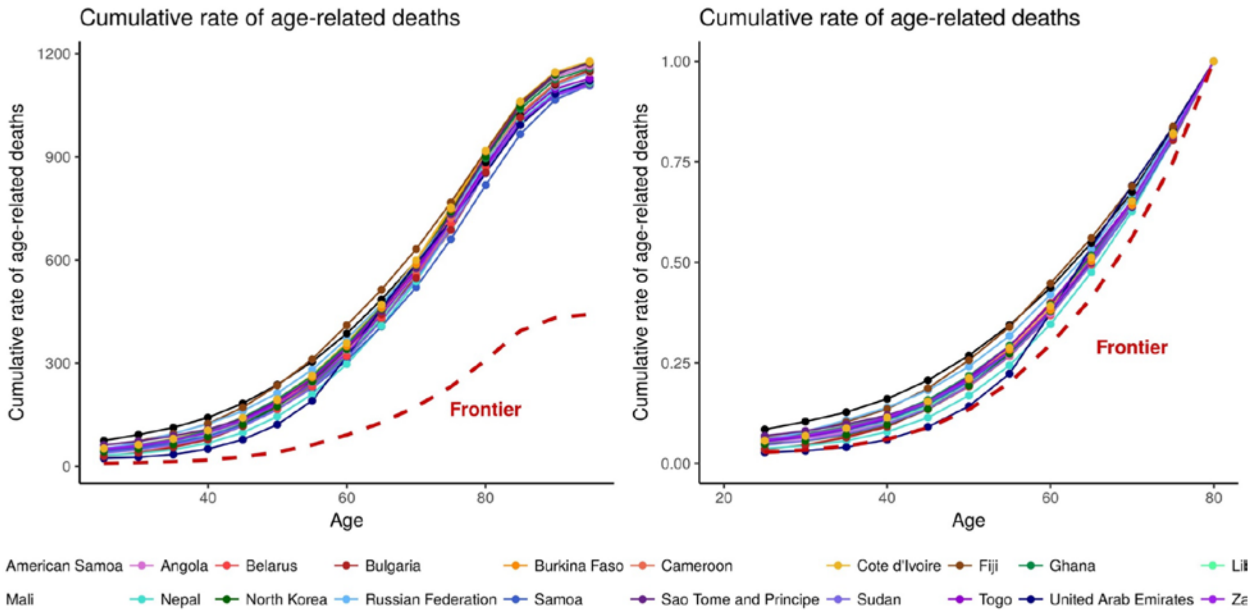

9th decile

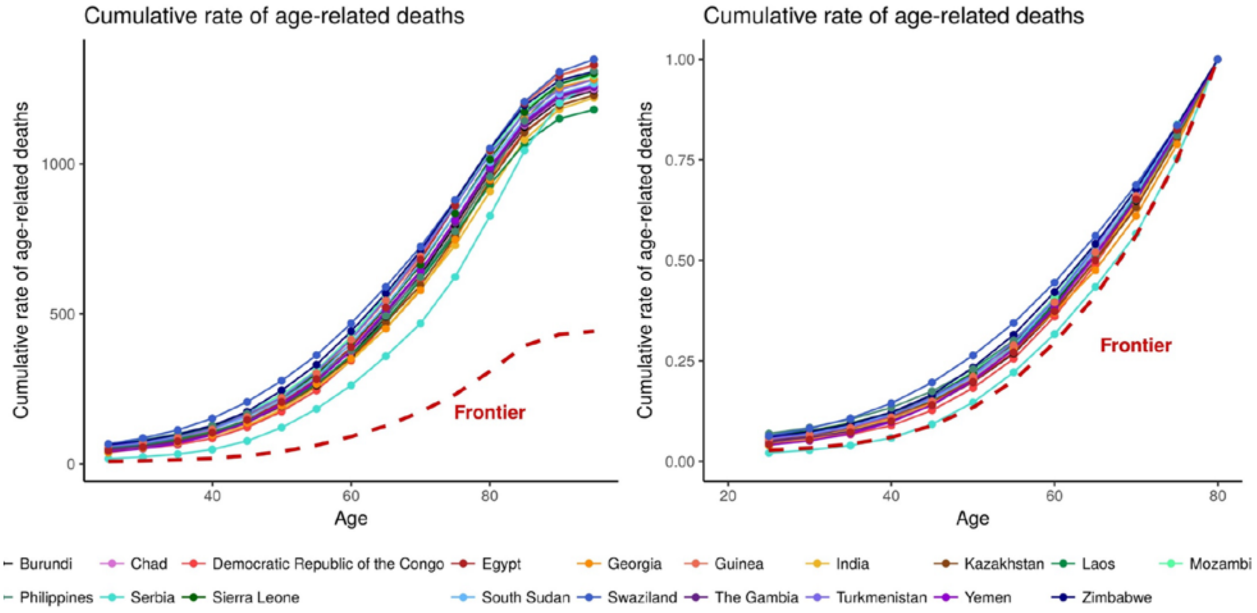

10th decile (ten countries with the highest age-standardised age-related death rate)

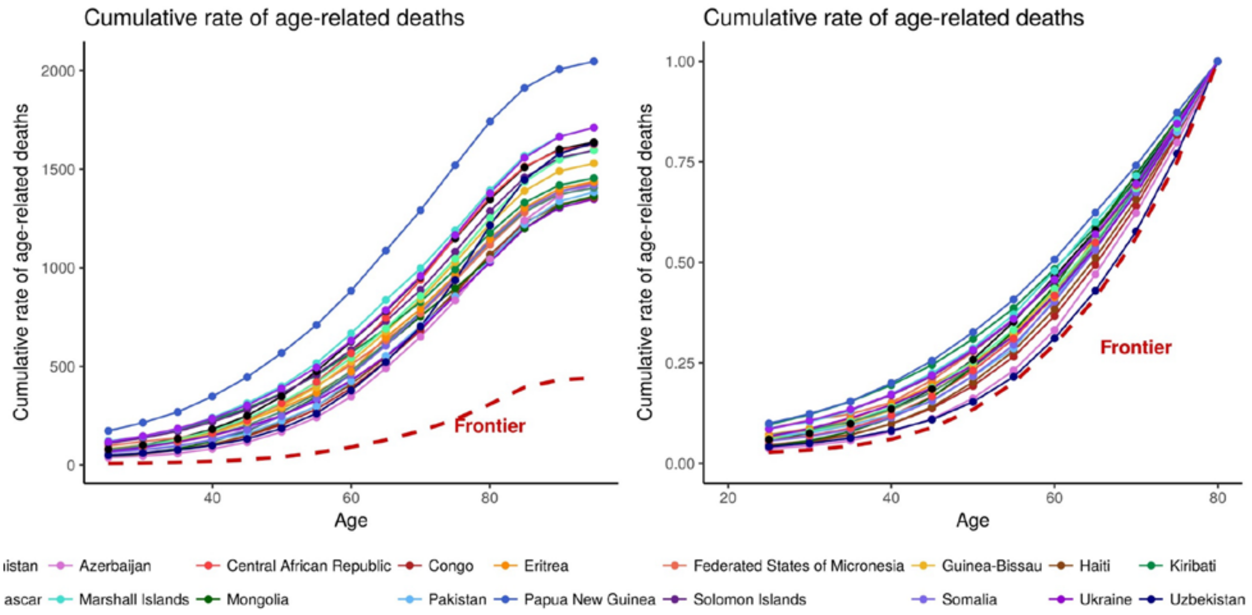

5. Sensitivity analysis

We included the following conditions that have been proposed by others as age-related, but did not match our criteria for defining age-related diseases: diabetes mellitus type 2, low back pain, neck pain, osteoarthritis, major depressive disorders, and anxiety disorders.[10–12]

The relationship between incidence rates and age groups for diabetes mellitus, low back and neck pain, osteoarthritis, major depressive disorders, and anxiety disorders

To identify age-related diseases, we followed a systematic approach assessing the relationship between age and incidence rates. There were several diseases that, prior to the assessment, one may consider as age-related, such as the diseases listed above. However, data suggested that these conditions did not meet our criteria (Appendix Figure 4, which can also be easily viewed in GBD Compare: https://vizhub.healthdata.org/gbd-compare/). For example, we saw that global age-specific incidence rates of most musculoskeletal disorders (except gout) do not follow the quadratic, convex pattern we were looking for. For example, incidence rates for low back pain and neck pain increase linearly but not quadratically with age; incidence rate for osteoarthritis increases from ages 25-50 but drops after age 50.

Appendix Figure 4. 2017 global incidence rates by age for diabetes mellitus type 2, low back pain, neck pain, osteoarthritis, major depressive disorders, and anxiety disorders

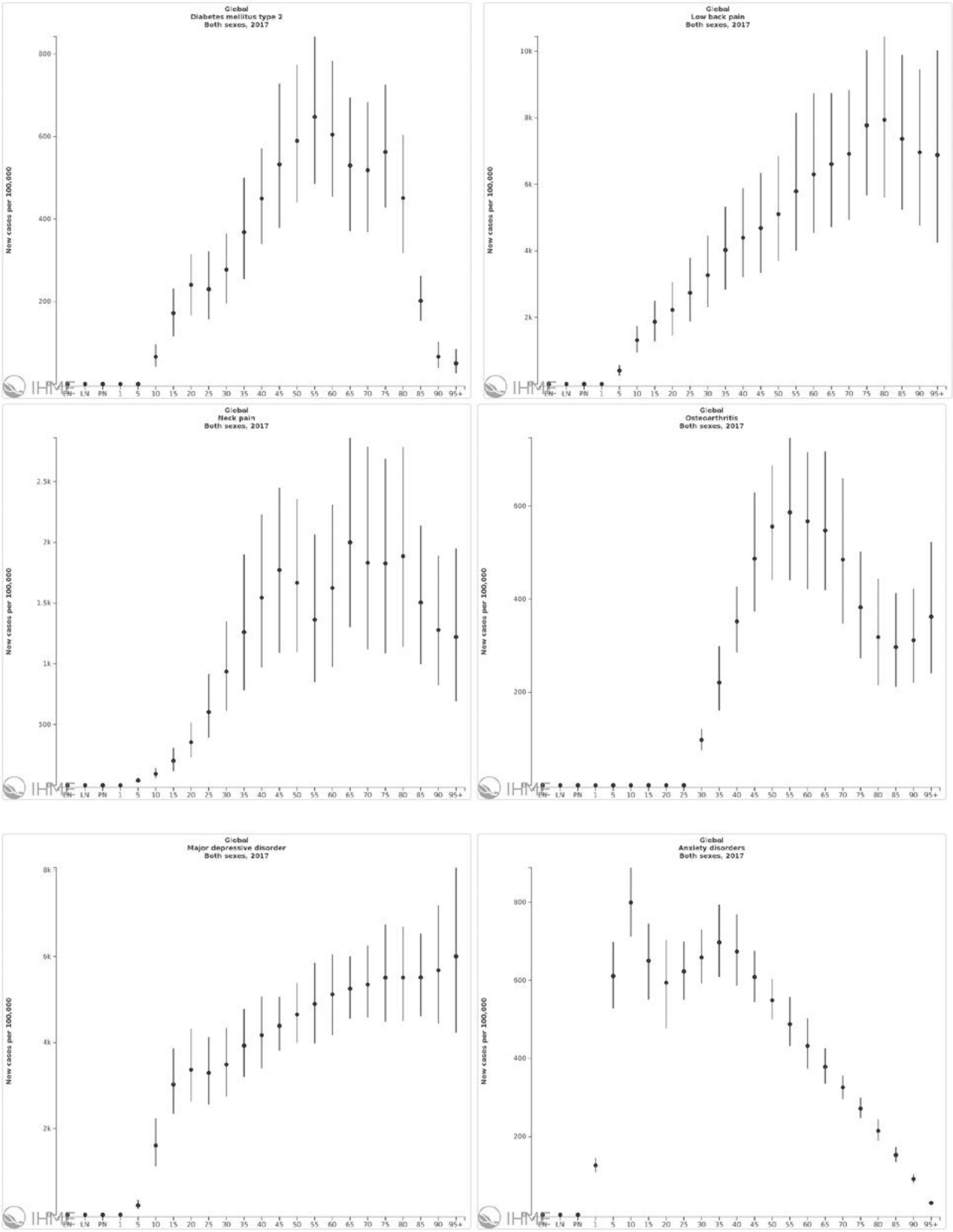

Global distribution of age-related and non-age-related burden

With the addition of the diseases listed above, we would expect 7.4% (6.7-8.2) of age-related burden to be from all diabetes and chronic kidney diseases, 4.2 (3.3, 5.2) from mental disorders, and 8.8 (6.9, 11.0) from musculoskeletal disorders.

Appendix Figure 5. Sensitivity analysis: Distribution of non-age-related (A) and age-related burden (B), global 2017

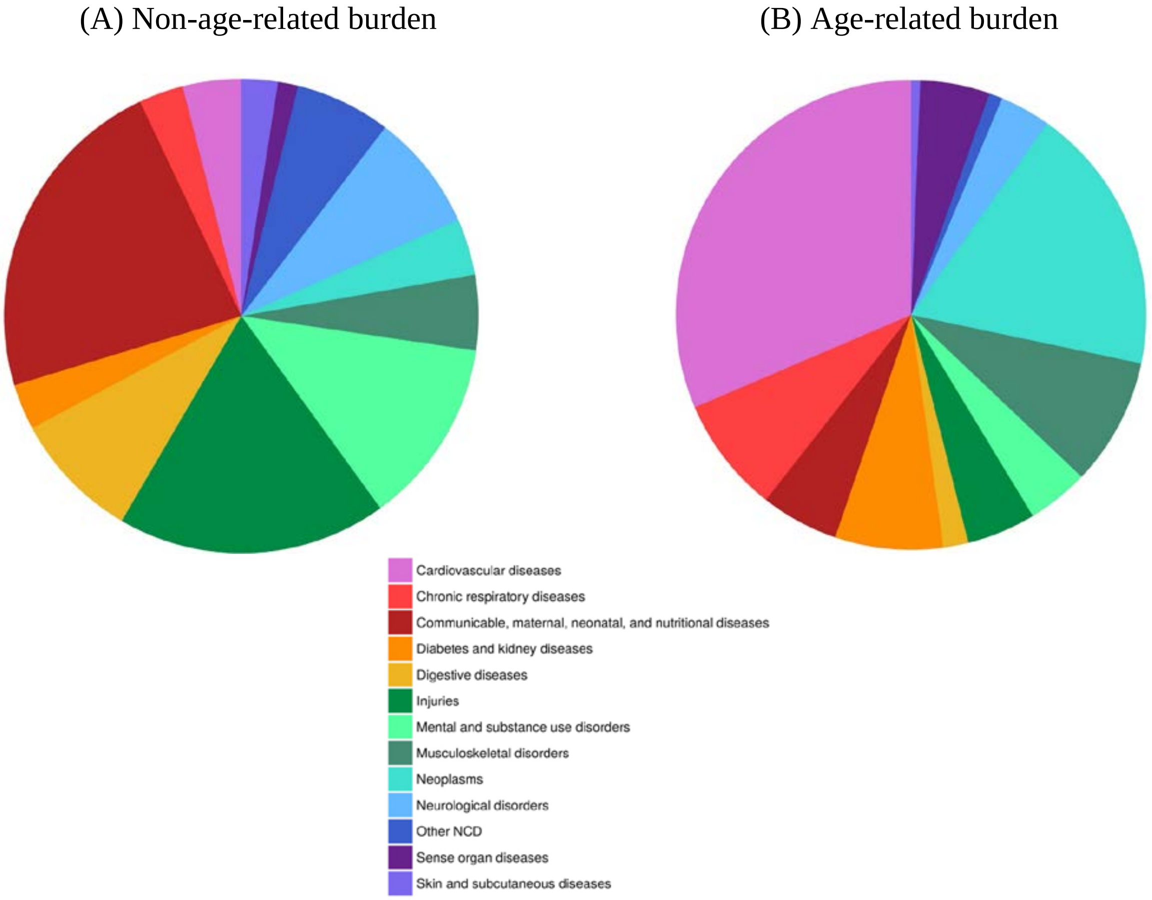

Appendix Table 8. Sensitivity analysis: distribution of non-age-related burden and age-related burden at the global level in 2017

GBD category	Non-age-related burden (%)	Age-related burden (%)
Communicable, maternal, neonatal, and nutritional diseases	22.6 (21.4, 23.9)	5.3 (4.8, 6.5)
Neoplasms	3.8 (3.4, 4.4)	18.4 (17, 19.9)
Cardiovascular diseases	4.0 (3.6, 4.4)	31.3 (29.3, 33.3)
Chronic respiratory diseases	3.0 (2.6, 3.6)	8 .0 (7.2, 8.6)
Digestive diseases	8.7 (8.2, 9.5)	1.7 (1.6, 1.9)
Neurological disorders	8.0 (6.1, 10.2)	3.5 (3.3, 3.7)
Mental and substance use disorders	12.7 (10.8, 14.4)	4.2 (3.3, 5.2)
Diabetes and kidney diseases	3.1 (2.8, 3.5)	7.4 (6.7, 8.2)
Other NCD	6.5 (5.2, 8.2)	0.9 (0.8, 1.0)

Musculoskeletal disorders	5.1 (3.9, 6.5)	8.8 (6.9, 11.0)
Skin and subcutaneous diseases	2.5 (1.9, 3.1)	0.6 (0.4, 1.1)
Sense organ diseases	1.4 (0.7, 2.5)	4.8 (3.6, 6.2)
Injuries	18.3 (16.9, 19.8)	4.7 (4.4, 5.2)

Estimating levels of age-related burden in 2017 across SDI and GBD regions

The addition of the six GBD causes would inevitably increase the proportion of age-related burden as a share of all disease burden among adults. Overall, the largest rates of age-standardised age-related burden are still observed in low-SDI and low-middle-SDI, and in South Asia and Central Europe, Eastern Europe, and Central Asia, the same as the original findings in the main manuscript. The gap between high-SDI and others would narrow down, as we estimate proportionally higher age-related burden (17% increase) than other SDIs. Similarly, across GBD regions, we estimate regions with lower age-related burden to have greater proportion of additional age-related burden compared to regions with higher age-related burden in the main findings (Appendix Figure 6). Pearson correlation coefficients between the sensitivity analysis and the original results for the ranks of age-standardised age-related burden rate and proportion of age-related burden in all burden are 99.4 and 88.6%, respectively.

Appendix Figure 6. Sensitivity analysis: proportion of health burden that is age-related burden (age-standardised), by socio-demographic Index and region, 2017

Panel A: Socio-demographic Index (SDI)

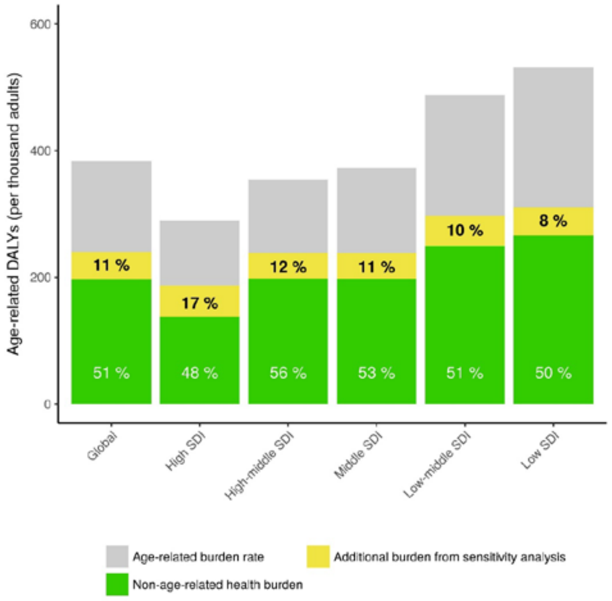

Panel B: GBD super-regions

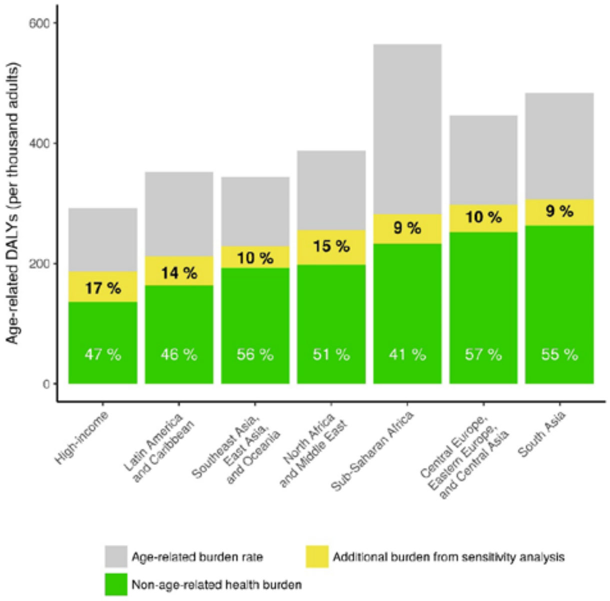

Analyzing the changes in age-related burden, 1990-2017

Estimated changes in age-related burden between 1990 and 2017 are estimated to be smaller with the addition of the six causes, with the differences between the sensitivity analysis and original results to be in the range of 1 to 6%. There is no change in the rankings across SDI and GBD regions.

Appendix Figure 7. Sensitivity analysis: change in age-related burden rate between 1990 and 2017, by socio-demographic Index and region

Panel A: Socio-demographic Index (SDI)

Panel B: GBD super-regions

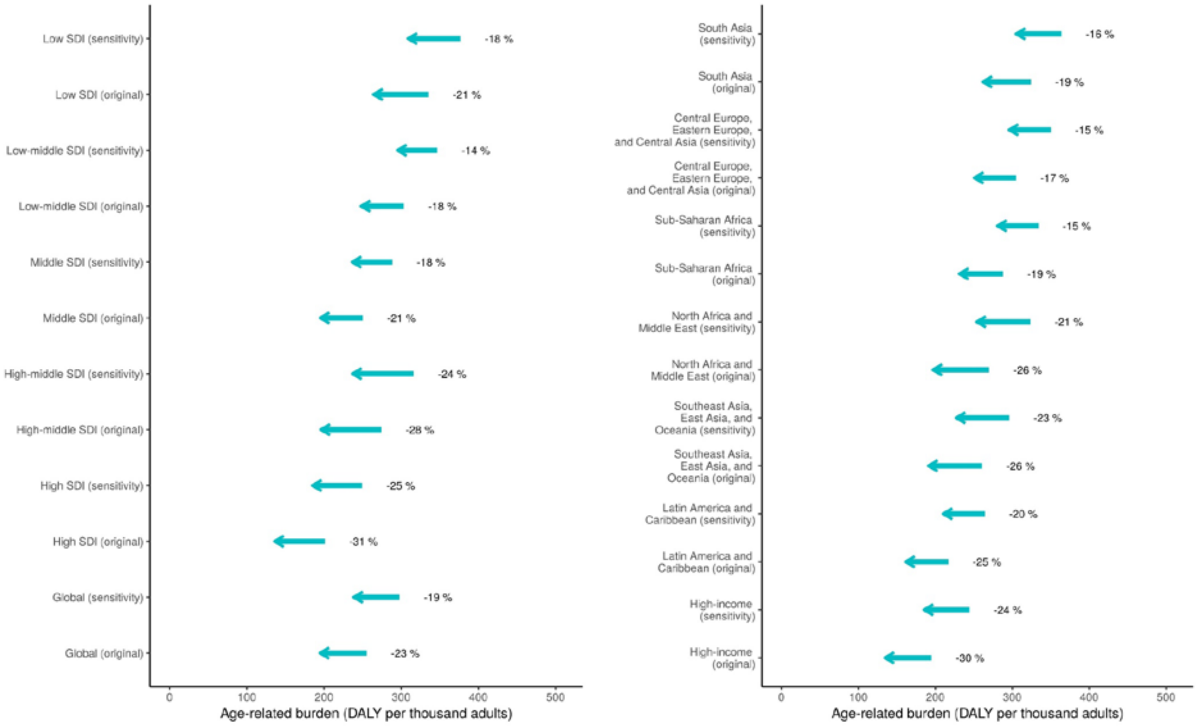

Appendix Figure 8. Sensitivity analysis: Decomposition of changes in age-related burden between 1996 and 2017, by socio-demographic Index and region

The percentages listed in the graphs are the change in DALYs associated with the factor compared to 1990 age-related DALYs

Panel A: Socio-demographic Index (SDI)

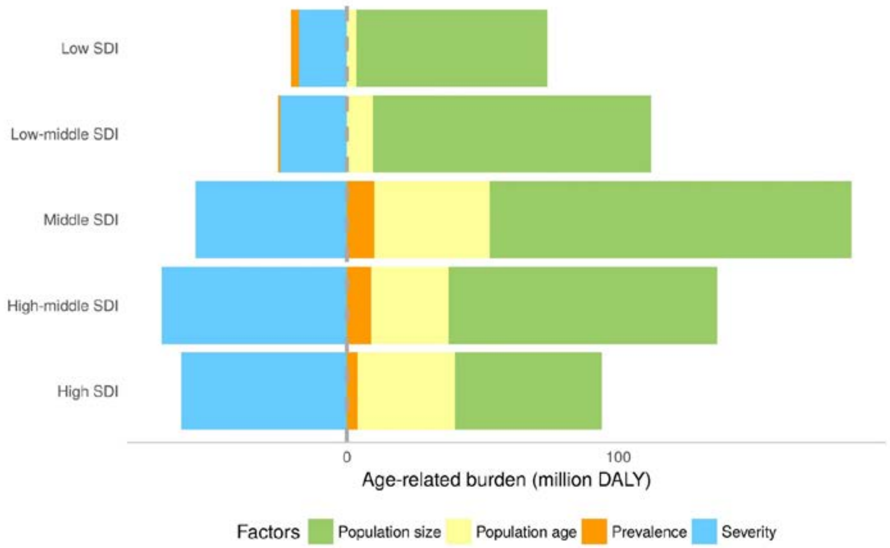

Appendix Table 9. Sensitivity analysis: results from the decomposition analysis, comparing results from the sensitivity analysis to the original results, by SDI

	Sensitivity analysis				Original result				Difference between original and sensitivity analysis results (percentage point)			
SDI	Population size (%)	Population age (%)	Prevalence (%)	Severity (%)	Population size (%)	Population age (%)	Prevalence (%)	Severity (%)	Population size (%)	Population age (%)	Prevalence (%)	Severity (%)
High	31.8	21.1	2.3	-35.9	31.2	24.0	1.9	-43.3	-0.6	2.8	-0.5	-7.4
High-middle	60.3	17.4	5.4	-41.6	59.6	18.8	7.0	-48.7	-0.7	1.4	1.6	-7.1
Middle	84.2	26.9	6.4	-35.3	83.1	28.9	6.4	-42.3	-1.1	2.0	0.0	-7.0
Low-middle	90.1	8.4	-0.6	-21.7	88.5	9.0	-2.3	-25.9	-1.6	0.6	-1.7	-4.3
Low	97.4	4.8	-4.0	-24.5	95.7	5.2	-5.7	-28.2	-1.7	0.4	-1.7	-3.6

Panel B: GBD super-regions

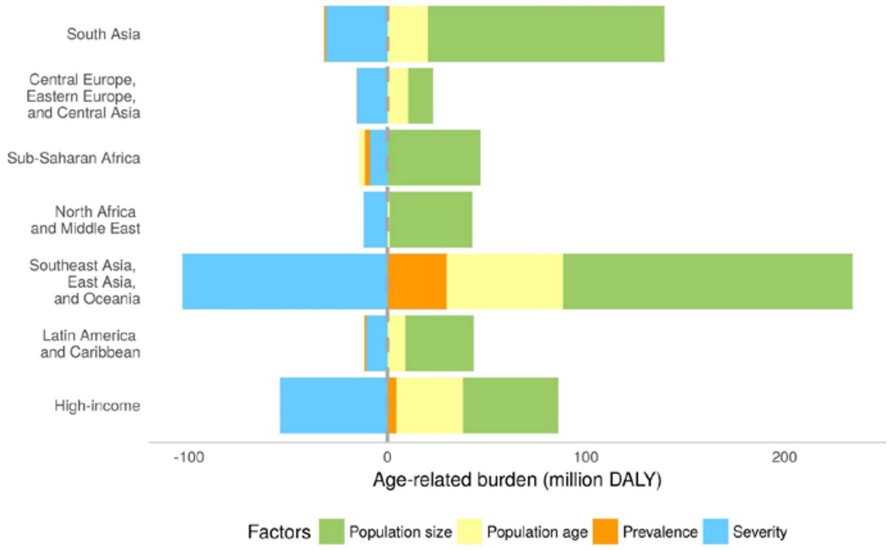

Appendix Table 10. Sensitivity analysis: results from the decomposition analysis, comparing results from the sensitivity analysis to the original results, by region

	Sensitivity analysis				Original result				Difference between original and sensitivity analysis results (percentage point)			
Region	Population size (%)	Population age (%)	Prevalence (%)	Severity (%)	Population size (%)	Population age (%)	Prevalence (%)	Severity (%)	Population size (%)	Population age (%)	Prevalence (%)	Severity (%)
High-income	30.4	21.2	2.9	-34.4	29.9	24.3	2.1	-41.7	-0.6	3.1	-0.8	-7.3
Latin America and Caribbean	97.6	25.6	-2.6	-30.1	95.7	28.1	-3.8	-38.1	-1.9	2.5	-1.2	-8.1
Southeast Asia, East Asia, and Oceania	74.5	30.1	15.1	-52.8	73.9	32.3	17.6	-61.8	-0.6	2.2	2.6	-8.9
North Africa and Middle East	124.9	4.0	0.0	-36.1	120.6	4.2	-3.2	-43.8	-4.2	0.2	-3.1	-7.8
Sub-Saharan Africa	108.4	-6.7	-5.6	-20.4	105.6	-7.1	-8.9	-24.0	-2.8	-0.4	-3.3	-3.6
Central Europe, Eastern Europe, and Central Asia	13.7	11.5	-0.3	-17.0	13.6	12.3	-0.2	-19.8	-0.1	0.9	0.1	-2.8
South Asia	95.4	16.3	-0.9	-24.8	94.2	17.5	-1.9	-29.2	-1.2	1.2	-1.0	-4.4

Appendix Table 11. Sensitivity analysis: country-level results, 2017 age-related burden rate (per 1,000 adults), 2017 age-related burden as share of all burden, and 2017 equivalent age to global 65-year-olds

		2017 age-related burden rate (per 1,000 adults)			2017 age-related burden as share of all burden (%)			2017 equivalent age to global 65-year-olds (years)		
Country	SDI	Sensitivity analysis	Original result	Dif (%)	Sensitivity analysis	Original result	Dif (%)	Sensitivity analysis	Original result	Dif (%)
Afghanistan	Low	446.8 (397.1, 496.7)	380.2 (340.4, 423.3)	17.5%	64.8 (62.8, 66.7)	55.1 (52.2, 58)	17.6%	51.2 (49, 53)	51.6 (49.2, 53.5)	-0.6%
Albania	High-middle	228.9 (195.7, 265)	185.4 (161.1, 215.3)	23.5%	73.4 (71.2, 75.4)	59.6 (55.4, 63.7)	23.1%	66.8 (65, 68.8)	66.7 (64.9, 68.7)	0.2%
Algeria	Middle	213.6 (189.4, 240.3)	161 (149.3, 173.6)	32.6%	66.8 (64.6, 68.7)	50.2 (46.3, 54.1)	32.9%	69.2 (68.3, 69.9)	69.7 (69.2, 70.2)	-0.7%
American Samoa	High-middle	348.8 (313.8, 384.7)	263.9 (242.1, 286)	32.2%	72.8 (71.4, 74)	55.1 (51.8, 58)	32.0%	57.1 (55.5, 59)	60.4 (58.6, 62.4)	-5.5%
Andorra	High	177.2 (153.7, 205)	126.1 (113.8, 140.6)	40.5%	67 (65.1, 68.9)	47.7 (43.4, 52.1)	40.6%	73.4 (71.9, 74.8)	73.6 (72.1, 74.9)	-0.3%
Angola	Low-middle	311.3 (274.6, 351.5)	253.3 (225.3, 283.9)	22.9%	52.1 (50.2, 54.2)	42.4 (39.8, 45.1)	23.0%	59.1 (57.3, 61.6)	60.4 (57.8, 62.2)	-2.1%
Antigua and Barbuda	High	212.3 (193, 235.3)	160.4 (150.8, 172.8)	32.3%	64.5 (62.6, 66.1)	48.8 (45.6, 51.8)	32.1%	67.1 (66.3, 68.1)	68.4 (67.6, 69.5)	-2.0%
Argentina	High-middle	230.9 (205.1, 261)	177.3 (161.5, 195.7)	30.2%	67.1 (65.6, 68.6)	51.5 (48, 55.1)	30.3%	65.8 (64.3, 67.4)	66.4 (64.8, 68.1)	-0.9%
Armenia	High-middle	263.9 (241.3, 289.7)	212.2 (200.2, 225.4)	24.4%	71.4 (69.9, 72.9)	57.5 (54.3, 60.9)	24.3%	63.1 (62.5, 63.6)	64.1 (63.6, 64.7)	-1.6%
Australia	High	172.4 (150, 196.6)	125.7 (112.5, 139.9)	37.1%	63.2 (61.3, 64.9)	46 (42.3, 50.1)	37.2%	73.6 (72.3, 74.9)	73.6 (72.3, 74.9)	0.0%
Austria	High	176.8 (154.9, 200.9)	130.3 (120, 142)	35.7%	64.6 (62.8, 66.4)	47.6 (43.9, 51.4)	35.8%	72.6 (71.6, 73.8)	73 (71.9, 74.1)	-0.5%
Azerbaijan	High-middle	329.9 (304, 360.1)	283 (265.7, 303.6)	16.6%	75 (73.2, 76.8)	64.4 (61, 67.7)	16.5%	58.6 (57.6, 59.6)	58.7 (57.7, 59.8)	-0.1%
Bahrain	High-middle	207.7 (182.3, 236.8)	131.1 (118.5, 143.5)	58.4%	67.8 (66.1, 69.8)	42.8 (39.8, 45.9)	58.6%	66.5 (65.5, 67.5)	71.2 (69.7, 72.4)	-6.6%
Bangladesh	Low-middle	265.4 (238.6, 293.9)	216.4 (197.8, 236.1)	22.6%	67.8 (66, 69.5)	55.2 (51.9, 58.5)	22.8%	62.4 (61.1, 63.7)	62.5 (61.1, 63.8)	0.0%
Barbados	High-middle	212.5 (191, 236.4)	159.6 (147.2, 172.9)	33.2%	65.2 (63.5, 66.7)	48.9 (45.8, 52.1)	33.4%	69 (67.8, 70.1)	70.5 (69.4, 71.7)	-2.1%
Belarus	High	294.8 (268.8, 323)	249.9 (234.8, 266.7)	18.0%	67.7 (66.2, 69)	57.5 (54.3, 60.3)	17.8%	60.8 (60.1, 61.4)	60.5 (59.6, 61.1)	0.5%
Belgium	High	192.1 (168, 220.3)	137.1 (126.2, 150.3)	40.1%	66.1 (64.5, 67.6)	47.3 (43.7, 51)	39.9%	71.4 (70.5, 72.4)	71.9 (71, 72.8)	-0.6%
Belize	Middle	231 (211.9, 253)	175 (165.7, 185.5)	32.0%	58.2 (57.2, 59.1)	44 (41.9, 46.2)	32.1%	66.1 (65.3, 66.8)	68.4 (67.5, 69.3)	-3.3%
Benin	Low	288 (250.3, 330.9)	238.2 (209.1, 275.7)	20.9%	54.9 (52.6, 56.9)	45.4 (42.7, 48.3)	20.8%	61.5 (58.2, 63.8)	61.8 (58.3, 64.3)	-0.4%
Bermuda	High	170.4 (152, 191.6)	130.5 (120.4, 141.8)	30.6%	65.5 (63.4, 67.4)	50.2 (46.5, 53.8)	30.3%	72.6 (71.7, 73.5)	72.7 (71.8, 73.7)	-0.2%
Bhutan	Low-middle	235.1 (200.8, 268.5)	190.5 (162.7, 216.5)	23.4%	63.6 (60.5, 65.7)	51.6 (47.4, 55.3)	23.2%	65.1 (62.8, 67.5)	65.2 (62.8, 67.6)	-0.1%
Bolivia	Middle	247 (209.9, 284.8)	194.8 (165.4, 224.9)	26.8%	63.3 (59.9, 65)	50 (45.8, 53.2)	26.6%	64.5 (61.9, 66.7)	65.2 (62.5, 67.5)	-1.0%
Bosnia and Herzegovina	High-middle	264.8 (238.5, 294.3)	212.3 (197, 229.7)	24.7%	72.7 (70.7, 74.5)	58.4 (55, 61.8)	24.4%	63.6 (62.7, 64.6)	64.4 (63.5, 65)	-1.2%
Botswana	Middle	265 (239.7, 295.5)	204.1 (188.1, 225.4)	29.8%	47.3 (43.3, 50.3)	36.3 (33.1, 39.4)	30.3%	63.1 (61.6, 64.1)	64.8 (63.1, 65.8)	-2.6%
Brazil	Middle	224.3 (205.1, 247.5)	174.4 (165.9, 184.6)	28.6%	61.2 (59.9, 62.3)	47.6 (44.9, 50.3)	28.8%	67.1 (66.8, 67.5)	67.6 (67.3, 68)	-0.8%
Brunei	High	255.7 (232, 284.6)	194.6 (180.8, 210)	31.4%	69.7 (68.4, 70.9)	53.1 (49.9, 55.9)	31.3%	63.6 (62.4, 64.7)	65.2 (63.9, 66.4)	-2.5%
Bulgaria	High-middle	302.4 (275.8, 331.7)	258.6 (241.4, 276.7)	17.0%	75.7 (74, 77.1)	64.7 (61.5, 67.9)	16.9%	59.3 (58.5, 60.4)	59 (58.2, 59.8)	0.6%
Burkina Faso	Low	309.8 (278.2, 346.7)	261.2 (235.8, 290.2)	18.6%	54.8 (52.4, 57.1)	46.1 (43.4, 49)	18.9%	60.2 (57.7, 61.8)	60.2 (57.7, 62)	-0.1%
Burundi	Low	313.4 (270.9, 354.4)	263.7 (226.9, 299.5)	18.9%	49.3 (46.5, 51.9)	41.5 (38.7, 44.5)	18.9%	59 (56.9, 61.7)	59.3 (56.9, 61.9)	-0.4%
Cambodia	Low-middle	283 (254.7, 313.5)	236 (216.7, 260.1)	19.9%	59.6 (58.1, 60.9)	49.7 (47.3, 52.1)	19.8%	61.6 (60.1, 62.9)	61.7 (60.2, 63)	-0.2%
Cameroon	Low-middle	299.3 (256.6, 344)	249.3 (212.5, 287.4)	20.0%	48.7 (46.1, 51.1)	40.6 (37.7, 43.4)	20.0%	60.6 (57.6, 63.1)	60.8 (57.6, 63.5)	-0.2%
Canada	High	172.4 (152.9, 196.3)	125.5 (116.2, 136.3)	37.3%	63.4 (61.7, 65.1)	46 (42.4, 50)	37.6%	72.8 (71.9, 73.7)	73.2 (72.3, 74)	-0.5%
Cape Verde	Low-middle	215.5 (192, 240.7)	168.8 (154.5, 182.6)	27.7%	59 (57.3, 60.4)	46.1 (43.6, 48.7)	27.8%	69 (68.2, 71.3)	70.9 (68.7, 71.9)	-2.7%
Central African Republic	Low	426.3 (367.4, 483.4)	364.6 (313.6, 418.4)	16.9%	44.1 (41.5, 46.4)	37.8 (35.1, 40.4)	16.6%	53.4 (50.9, 55.7)	53.6 (50.8, 56)	-0.3%
Chad	Low	326.6 (289.4, 368.9)	275.4 (245.8, 310.9)	18.6%	53.3 (51.2, 55.6)	45 (42.5, 47.7)	18.6%	58.6 (56.3, 61.1)	58.6 (56.2, 61.2)	-0.1%
Chile	High-middle	196.9 (173.1, 224)	138.1 (125.9, 153.2)	42.5%	64.1 (62.6, 65.3)	45.1 (41.5, 48.6)	42.0%	70.3 (69, 71.7)	71.1 (69.8, 72.6)	-1.2%
China	Middle	215.9 (198.7, 235.8)	183.9 (173.9, 194.9)	17.4%	68 (66.2, 69.8)	57.9 (54.5, 61.3)	17.6%	66.7 (66.3, 67.1)	66 (65.7, 66.4)	1.0%
Colombia	High-middle	157.9 (138.2, 178.6)	124.4 (111.3, 137.3)	26.9%	57.6 (56.2, 59)	45.4 (42.8, 48.2)	26.9%	74.3 (72.8, 75.7)	73.7 (72.2, 75.3)	0.7%
Comoros	Low	289.4 (255.4, 328.1)	240.3 (212.5, 272.2)	20.4%	62 (59.8, 64.3)	51.5 (48.4, 54.8)	20.4%	61.1 (58.6, 63.2)	61.3 (58.8, 63.4)	-0.3%

Congo	Low-middle	348.2 (302.9, 392.3)	287.9 (249.5, 323.8)	20.9%	51.5 (49, 53.6)	42.5 (40, 45)	21.0%	57.3 (55.5, 59.3)	57.9 (56, 60.4)	-1.2%
Costa Rica	High-middle	179.3 (162.5, 199.6)	145.2 (135.2, 156.9)	23.5%	61.8 (60.2, 63.2)	50.1 (47.2, 53)	23.2%	71.4 (70.6, 72.1)	70.8 (70.1, 71.6)	0.8%
Cote d'Ivoire	Low	308.4 (272.9, 347.6)	260.7 (231.2, 294.2)	18.3%	51.3 (48.6, 53.5)	43.4 (40.4, 45.9)	18.2%	59.6 (57.2, 62.1)	59.5 (57, 62.2)	0.2%
Croatia	High-middle	234.4 (210.7, 263.9)	183.7 (170.8, 199.6)	27.6%	71.6 (70.1, 73)	56.1 (52.6, 59.7)	27.7%	65.6 (64.6, 66.5)	66.1 (65.2, 67)	-0.8%
Cuba	High-middle	206.4 (185.7, 229.8)	169.4 (155.2, 185)	21.9%	67 (64.8, 68.8)	54.9 (51.2, 58.5)	22.0%	67.2 (65.6, 68.9)	66.7 (65.1, 68.3)	0.8%
Cyprus	High	184.4 (161, 210.6)	129.9 (118.4, 142.4)	41.9%	68.3 (66.6, 70.2)	48.2 (44.4, 52.5)	41.8%	71.2 (70.1, 72.2)	72.1 (71, 73.1)	-1.2%
Czech Republic	High	234.9 (208.7, 267.3)	185.5 (169.4, 202.8)	26.7%	71.8 (70.5, 73.2)	56.7 (53.5, 59.9)	26.7%	65.4 (64.3, 66.4)	65.7 (64.6, 66.7)	-0.5%
Democratic Republic of the Congo	Low	316.5 (275.3, 355.7)	260.8 (227.9, 294.5)	21.4%	50.8 (48.1, 53.1)	41.8 (38.7, 44.8)	21.3%	58.8 (56.8, 61.4)	59.4 (57.1, 61.9)	-1.0%
Denmark	High	194.8 (172.2, 223.8)	137.6 (127.4, 149)	41.6%	67.2 (65.8, 68.6)	47.3 (43.6, 51.2)	42.1%	70.4 (69.1, 71.3)	71.1 (70.3, 72)	-1.0%
Djibouti	Low-middle	283.8 (228.7, 349.3)	233.5 (183.6, 297.2)	21.6%	55.6 (53, 57.9)	45.6 (42.2, 48.9)	22.1%	61 (56.4, 64.7)	61.3 (56.6, 65.2)	-0.6%
Dominica	High-middle	251.4 (230.6, 274.4)	198.7 (186.3, 211.7)	26.5%	65.7 (64.1, 67)	51.9 (49, 54.8)	26.5%	64.8 (64.2, 65.4)	65.1 (64.4, 66)	-0.4%
Dominican Republic	High-middle	234.3 (210.1, 262.6)	190.6 (171, 211.8)	22.9%	59.6 (57.7, 61.4)	48.4 (45.4, 51.4)	23.2%	65.2 (64.2, 67.1)	65.2 (64.2, 67.2)	-0.1%
Ecuador	High-middle	195 (174.4, 219.1)	147.1 (135, 162.7)	32.5%	59.3 (58.3, 60.4)	44.8 (42.3, 47.3)	32.4%	71.6 (70.3, 72.9)	72.6 (71.2, 73.9)	-1.4%
Egypt	Middle	345.3 (313, 383.4)	286 (262.7, 313.8)	20.7%	69.4 (67.4, 72.1)	57.5 (53.9, 61.4)	20.6%	57.4 (56.1, 58.8)	58 (56.5, 59.5)	-0.9%
El Salvador	Middle	226.7 (199.4, 261.4)	177.7 (155.3, 203.1)	27.6%	56.2 (54.3, 57.9)	44 (41.6, 46.5)	27.5%	67.4 (64.9, 70.3)	68.4 (65.9, 71.1)	-1.5%
Equatorial Guinea	Middle	258.8 (205.3, 328.8)	195.6 (147.4, 255.9)	32.3%	44.9 (40.7, 48.4)	33.9 (29.8, 37.9)	32.4%	63.2 (58.3, 67.5)	64.7 (60.1, 69.6)	-2.3%
Eritrea	Low	374.7 (322.1, 427.2)	325.7 (277.4, 374.9)	15.0%	52.8 (49.7, 55.2)	45.8 (42.7, 48.7)	15.3%	56.7 (54.3, 59)	56.6 (54.1, 59.1)	0.1%
Estonia	High	228.5 (200, 258.3)	181.9 (163.2, 202.2)	25.6%	64.7 (63.3, 65.9)	51.5 (48.2, 54.6)	25.5%	66 (64.4, 68.1)	66.1 (64.5, 68.4)	-0.2%
Ethiopia	Low	254.4 (229.8, 281.7)	208.9 (192.5, 226.9)	21.8%	55.9 (54.3, 57.6)	45.9 (43.8, 48.2)	21.7%	63.6 (62.6, 64.5)	63.8 (62.7, 64.8)	-0.3%
Federated States of Micronesia	Middle	414.2 (358.3, 472.8)	325.1 (275.5, 371.1)	27.4%	68.8 (60.2, 71.1)	53.7 (45.7, 57.4)	28.1%	52.6 (50.3, 54.8)	55 (52.4, 57.7)	-4.4%
Fiji	High-middle	406.7 (366.9, 455)	267.3 (241.3, 299.2)	52.1%	73.3 (72, 74.5)	48.2 (45.8, 50.4)	52.0%	53 (51.4, 54.6)	58.1 (56.1, 61.6)	-8.9%
Finland	High	183.2 (160.6, 210.4)	131.5 (121.1, 144.3)	39.4%	63.4 (62.1, 64.7)	45.5 (42.1, 49.2)	39.3%	72 (71, 72.9)	72.3 (71.4, 73.2)	-0.4%
France	High	167.5 (146.9, 192.1)	119.3 (109.3, 130.6)	40.5%	64.4 (62.9, 65.7)	45.8 (42.1, 49.5)	40.6%	75.9 (75, 76.8)	76 (75.1, 76.9)	-0.1%
Gabon	Middle	289.1 (257.8, 324.3)	227 (203.8, 253.6)	27.4%	55.2 (52.9, 57.3)	43.5 (40.7, 45.9)	27.1%	60.8 (58.4, 62.2)	61.8 (59.7, 63.4)	-1.7%
Georgia	High-middle	314.7 (291.5, 341.5)	268.5 (255.7, 282.2)	17.2%	71.5 (70.1, 72.8)	61 (57.9, 64)	17.2%	60 (59.5, 60.6)	60.1 (59.7, 60.6)	-0.1%
Germany	High	198.5 (174.5, 227.8)	144.7 (131.5, 160.1)	37.2%	68.2 (66.6, 69.8)	49.7 (45.8, 53.8)	37.2%	70.4 (68.8, 71.7)	70.7 (69.1, 72.1)	-0.5%
Ghana	Low-middle	303.7 (273.8, 337.6)	256.4 (231.4, 282.7)	18.4%	54.8 (52.3, 56.9)	46.1 (43.1, 48.8)	18.7%	60.2 (57.9, 61.8)	60.3 (57.9, 62.1)	-0.1%
Greece	High-middle	198.5 (176.6, 224)	147.6 (137.4, 159.1)	34.5%	69.6 (67.5, 71.6)	51.8 (47.5, 56.1)	34.4%	71.2 (70.4, 72.1)	71.4 (70.6, 72.3)	-0.3%
Greenland	High-middle	273.7 (251.8, 299.3)	224.5 (212, 236.8)	21.9%	65.3 (63.8, 66.6)	53.5 (50.3, 56.6)	22.0%	60.9 (60.4, 61.7)	60.7 (60.2, 61.4)	0.4%
Grenada	High-middle	265.2 (244.8, 291.1)	208 (195.9, 221)	27.5%	67.8 (66.3, 69.2)	53.1 (50.2, 55.9)	27.7%	62 (61.3, 62.8)	63.4 (62.5, 64.4)	-2.2%
Guam	High	301.7 (275, 331.8)	247.6 (231, 266.1)	21.8%	69.8 (68.5, 71.1)	57.4 (54.3, 60.2)	21.6%	59.5 (58.2, 61.9)	61.4 (58.9, 62.9)	-3.2%
Guatemala	Low-middle	234 (208.7, 259.1)	175.2 (160.1, 190.6)	33.6%	57.4 (56.6, 58.4)	43.1 (41.1, 45)	33.4%	67.6 (65.9, 69.3)	69.6 (68, 71.2)	-3.0%
Guinea	Low	339.2 (301.8, 377.2)	292.7 (262.5, 323.3)	15.9%	53.7 (51.7, 55.4)	46.2 (43.9, 48.5)	16.2%	57.2 (55.5, 59.3)	57 (55.3, 59)	0.4%
Guinea-Bissau	Low	395.7 (351, 444.3)	343.4 (306.4, 386.7)	15.2%	54 (51.3, 56.5)	46.9 (43.9, 49.8)	15.2%	54.7 (52.8, 56.3)	54.5 (52.4, 56.2)	0.3%
Guyana	Middle	313.3 (282.8, 345.4)	247.7 (225.7, 271.1)	26.5%	61 (59.8, 62.1)	48.3 (45.7, 50.7)	26.4%	58.1 (56.5, 59.6)	59.6 (57.8, 61.5)	-2.5%
Haiti	Low-middle	340.1 (297.1, 387.2)	285.5 (249, 325)	19.1%	59.5 (57.3, 61.1)	49.9 (46.8, 52.6)	19.3%	57.1 (55.1, 60.3)	57.5 (55.3, 60.7)	-0.7%
Honduras	Middle	229.4 (198.6, 264.2)	189.5 (164.2, 218.4)	21.0%	58.4 (56.8, 59.8)	48.3 (45.5, 51.2)	20.8%	66.1 (64.2, 68.4)	65.9 (64, 68.2)	0.3%
Hungary	High	270.8 (244.6, 302.1)	219.6 (204.7, 237.5)	23.3%	72.9 (71.6, 74.1)	59.1 (56, 62.3)	23.3%	60.7 (59.9, 61.6)	61 (60.1, 61.8)	-0.4%
Iceland	High	174.8 (153.9, 201.1)	124.8 (115.2, 136.2)	40.1%	67.2 (65.5, 68.9)	47.9 (44.2, 52)	40.2%	72.7 (72, 73.4)	73 (72.4, 73.6)	-0.4%
India	Low-middle	307.5 (286.3, 332.2)	265.6 (252.9, 279.7)	15.8%	62.8 (61.2, 64.3)	54.3 (51.7, 56.8)	15.8%	59.8 (59.4, 60.2)	59.6 (59.1, 60)	0.4%
Indonesia	Middle	287.4 (263.3, 312)	235.8 (220.9, 250.1)	21.9%	66.1 (64.8, 67.3)	54.3 (51.8, 56.8)	21.9%	61.2 (60.5, 62)	61.7 (61, 62.5)	-0.9%
Iran	High-middle	227.2 (204.7, 254.1)	164.8 (156.3, 174.7)	37.9%	65.6 (63.9, 67.2)	47.5 (44.1, 51.2)	38.0%	67.6 (67.2, 68.1)	69 (68.7, 69.3)	-2.0%
Iraq	Middle	204.2 (180.3, 231.9)	147.5 (135.7, 160.6)	38.5%	57.3 (54.8, 59.2)	41.4 (38.5, 44.3)	38.5%	68.9 (67.6, 70.6)	70.8 (69.2, 72)	-2.6%
Ireland	High	182.4 (160.9, 208.5)	131.1 (120.4, 142.9)	39.1%	66.7 (64.9, 68.6)	47.9 (43.8, 52.2)	39.2%	71.7 (70.8, 72.5)	71.9 (71.1, 72.8)	-0.4%
Israel	High	171.8 (149, 197.8)	120.2 (109.6, 133)	42.9%	67.3 (65.6, 69)	47.2 (43.6, 51.2)	42.7%	73.2 (72.3, 74.1)	74 (73.2, 75.6)	-1.1%
Italy	High	166.1 (145.1, 191.1)	115.2 (105.5, 126.8)	44.1%	66.6 (64.8, 68.6)	46.2 (42.4, 50.8)	44.1%	74.5 (73.5, 75)	74.8 (74.3, 75.4)	-0.4%

Jamaica	High-middle	238.8 (213.1, 270.4)	175.3 (157.2, 197.2)	36.2%	63.1 (61.6, 64.6)	46.3 (43.4, 49.1)	36.4%	64.1 (62.3, 65.8)	66.2 (64.6, 68.1)	-3.1%
Japan	High	154.9 (134.1, 179.8)	110.6 (101.3, 121.7)	40.1%	65.8 (64.4, 67)	47 (43.5, 50.4)	40.1%	76.4 (75.7, 77)	76.1 (75.6, 76.7)	0.3%
Jordan	High-middle	207.9 (184.8, 236.8)	149.3 (136.2, 164.8)	39.3%	68 (65.9, 70.2)	48.7 (44.9, 53)	39.7%	68.9 (67, 70.2)	70 (69, 71.1)	-1.6%
Kazakhstan	High-middle	310.9 (288.4, 337.3)	266.9 (253.9, 282.9)	16.5%	66.9 (65.6, 68.2)	57.4 (54.7, 60.1)	16.4%	59.8 (59.2, 60.3)	59.7 (58.9, 60.1)	0.2%
Kenya	Low-middle	277.3 (255.5, 301.8)	228.4 (214.6, 242.8)	21.4%	48.6 (46.9, 50.2)	40 (38, 42)	21.5%	61.8 (61, 62.6)	62.1 (61.4, 62.9)	-0.5%
Kiribati	Low-middle	474.9 (425.6, 526.1)	347.5 (311.8, 386.3)	36.7%	65.3 (64.3, 66.4)	47.8 (45.8, 49.7)	36.6%	49.3 (47.1, 51.6)	54.2 (51.3, 56.1)	-9.1%
Kuwait	High	170.2 (147.7, 197.5)	118.2 (107.7, 129.7)	44.0%	66 (64.2, 68)	45.9 (42.4, 49.7)	44.0%	74.9 (73.8, 75.6)	75.3 (74.6, 76.4)	-0.6%
Kyrgyzstan	Low-middle	273.7 (253.8, 297.4)	232.7 (222.1, 245.2)	17.6%	65.1 (63.6, 66.4)	55.3 (52.4, 58.1)	17.7%	61.6 (61.1, 62)	61.3 (60.9, 61.7)	0.4%
Laos	Low-middle	309.1 (270.2, 347.2)	260.3 (227.4, 292.4)	18.8%	65.4 (61.7, 67.2)	55.1 (50.6, 58)	18.7%	59.2 (57.1, 61.6)	59.4 (57.3, 61.8)	-0.4%
Latvia	High	277.7 (246.5, 307.5)	228.2 (206.2, 248.7)	21.7%	66.4 (65.1, 67.6)	54.6 (51.5, 57.7)	21.6%	61.6 (60.1, 63.2)	61.7 (59.9, 63.4)	-0.2%
Lebanon	High-middle	246.6 (222.5, 275.5)	187.2 (174.6, 201.2)	31.7%	69.3 (67.2, 71.6)	52.6 (48.9, 56.9)	31.7%	65.4 (64.6, 66.4)	66.5 (65.4, 67.5)	-1.6%
Lesotho	Low-middle	441.3 (379.8, 499.2)	360.5 (308.7, 410.9)	22.4%	43.6 (40.3, 46.2)	35.6 (32.7, 38.3)	22.4%	52.8 (50.4, 55.1)	53.6 (51, 56.1)	-1.6%
Liberia	Low	294.2 (259.3, 334.7)	244.6 (216, 274.3)	20.3%	50.8 (48.3, 53.3)	42.3 (39.3, 45.4)	20.2%	61.1 (58.1, 63.3)	61.3 (58.1, 63.7)	-0.3%
Libya	Middle	299.7 (262.7, 337)	236.2 (210.1, 259.9)	26.9%	66.9 (65, 69.1)	52.8 (49.2, 56.4)	26.6%	61.6 (58.7, 63.2)	62.6 (61.2, 64.3)	-1.6%
Lithuania	High	281.6 (254.5, 310.7)	231.9 (217.2, 248.9)	21.4%	66.1 (65, 67)	54.7 (51.8, 57.4)	20.9%	61.4 (60.6, 62.3)	61.5 (60.6, 62.4)	0.0%
Luxembourg	High	189.3 (165.3, 217.6)	136.1 (124.5, 150.6)	39.1%	66.4 (64.6, 68)	47.8 (43.9, 51.9)	39.0%	71.4 (70.2, 72.5)	71.8 (70.6, 72.9)	-0.6%
Macedonia	High-middle	263.1 (237.9, 291.6)	212.4 (197.5, 229.2)	23.9%	75.4 (73.6, 77.2)	60.9 (57.3, 64.5)	23.8%	62.8 (61.9, 63.7)	63.4 (62.5, 64.3)	-0.9%
Madagascar	Low	361.6 (315.7, 411.9)	314 (273.7, 359.8)	15.2%	62.7 (60.3, 64.7)	54.5 (51.4, 57.5)	15.1%	56.1 (54.4, 58.8)	56 (53.9, 58.7)	0.3%
Malawi	Low	289.8 (258.2, 325.1)	240.3 (214.9, 268.2)	20.6%	48.9 (46.6, 51.4)	40.5 (37.9, 43.4)	20.7%	61 (58.9, 62.7)	61.3 (59.2, 63)	-0.4%
Malaysia	High-middle	265.2 (241.7, 291.9)	223.9 (208.2, 242.2)	18.4%	70.4 (68.8, 72)	59.4 (56.1, 63)	18.5%	62.8 (61.7, 63.9)	62.5 (61.4, 63.5)	0.5%
Maldives	Middle	174.9 (155.9, 196.8)	133.8 (123.6, 145.2)	30.7%	68 (65.8, 70)	52.1 (48.2, 55.9)	30.6%	70.8 (69.5, 71.8)	71 (69.8, 72.1)	-0.4%
Mali	Low	293.7 (260.1, 328.9)	250.8 (222.2, 279.4)	17.1%	54.8 (52.4, 57.5)	46.8 (43.9, 50)	17.1%	61.3 (59, 63.1)	61.1 (58.7, 63)	0.3%
Malta	High-middle	197.7 (174.8, 225.9)	140.7 (130.9, 152.6)	40.5%	70.8 (68.9, 72.6)	50.4 (46.3, 54.8)	40.4%	70.2 (69.5, 70.8)	70.9 (70.4, 71.4)	-1.0%
Marshall Islands	Middle	481.8 (433.3, 534.5)	396.9 (358.3, 442.7)	21.4%	70.4 (68.7, 72.1)	58.1 (55.2, 60.6)	21.3%	49.9 (48.1, 51.7)	51 (49.3, 53.3)	-2.3%
Mauritania	Low	269.7 (237, 306)	220.5 (193.9, 248.4)	22.3%	62.7 (60.9, 64.5)	51.3 (48.6, 54.2)	22.3%	62.3 (60.3, 64.4)	62.6 (60.4, 64.9)	-0.5%
Mauritius	High-middle	263.4 (239.2, 289.3)	182.6 (170.8, 195.9)	44.3%	67 (65.5, 68.4)	46.4 (43.9, 49)	44.4%	63.2 (62.2, 64.1)	67.1 (66.2, 68.1)	-5.9%
Mexico	Middle	215.5 (196.5, 237.7)	156.2 (147.7, 166.1)	37.9%	59.8 (58.9, 60.6)	43.4 (41.2, 45.5)	37.9%	67.9 (67.4, 68.4)	70.3 (69.9, 70.7)	-3.3%
Moldova	High-middle	296.8 (274.4, 323.4)	250.8 (239.2, 264.5)	18.4%	66.5 (65.3, 67.6)	56.2 (53.4, 59.1)	18.3%	59.1 (58.6, 59.9)	58.9 (58.4, 59.4)	0.3%
Mongolia	High-middle	347.5 (317.7, 378.7)	307.4 (284.5, 330.9)	13.1%	66.4 (65.1, 67.6)	58.7 (56.1, 61.2)	13.1%	56 (54.8, 57.3)	55.3 (54.2, 56.6)	1.2%
Montenegro	High-middle	270.4 (244.6, 302)	223 (206, 243.1)	21.3%	73.5 (71.9, 74.9)	60.6 (57, 64.2)	21.2%	62.3 (61.1, 63.4)	62.3 (61.1, 63.4)	0.0%
Morocco	Low-middle	285.9 (249, 326.7)	222.9 (195.4, 252.9)	28.3%	69.5 (67.3, 71.3)	54.2 (49.8, 58.2)	28.3%	62.2 (60.2, 64.4)	63.1 (61, 65.2)	-1.4%
Mozambique	Low	343 (302.3, 386.6)	291.4 (255.8, 328.6)	17.7%	43.9 (40.5, 46.4)	37.3 (34.1, 39.8)	17.7%	56.9 (55.1, 59)	57 (55.2, 59.3)	-0.3%
Myanmar	Low-middle	289.4 (258.3, 318.3)	239.6 (216, 262.5)	20.8%	60.1 (58.8, 61.4)	49.7 (47.6, 51.9)	20.9%	61.1 (59.5, 62.5)	61.6 (60.3, 63)	-0.7%
Namibia	Middle	278.6 (247.6, 311.1)	224.1 (201.9, 251.1)	24.3%	47.5 (43.4, 50.3)	38.2 (34.6, 41.3)	24.2%	61.8 (59.9, 63.3)	62.6 (60.7, 64.4)	-1.4%
Nepal	Low-middle	290.6 (258.6, 321.2)	241.9 (214.7, 264.5)	20.1%	63.9 (60.2, 65.8)	53.1 (48.6, 56.3)	20.3%	60.7 (59.1, 62.4)	60.7 (59.1, 62.6)	0.0%
Netherlands	High	190.5 (168, 216.7)	133.8 (123.6, 144.4)	42.4%	68.7 (66.8, 70.4)	48.1 (44.1, 52.7)	42.6%	71.4 (70.5, 72.2)	71.9 (71.1, 72.7)	-0.7%
New Zealand	High	192.6 (170.2, 221.1)	138 (126.8, 152)	39.6%	66.4 (64.6, 68)	47.5 (43.9, 51.6)	39.7%	72.2 (71.3, 73.1)	72.5 (71.6, 73.3)	-0.5%
Nicaragua	Middle	178.4 (158, 201.7)	134.1 (120.8, 148.2)	33.1%	59.2 (57.6, 60.7)	44.5 (41.9, 47.1)	33.1%	72.8 (71.1, 74.4)	73.6 (72, 75.1)	-1.1%
Niger	Low	288.1 (254.6, 325.9)	241.8 (214.4, 273)	19.1%	54.3 (51.1, 56.9)	45.6 (42, 48.7)	19.2%	61.4 (58.8, 63.5)	61.4 (58.7, 63.7)	0.0%
Nigeria	Low-middle	231 (189, 290.2)	186.1 (150.5, 242)	24.1%	45.8 (42.9, 48.7)	36.8 (33.4, 40.6)	24.3%	66 (61.8, 69.3)	66.1 (61.7, 69.5)	-0.1%
North Korea	Middle	290.6 (258.6, 321.2)	256.2 (229.2, 282.1)	13.4%	68.9 (66.2, 70.9)	60.8 (57.1, 64.1)	13.3%	60.4 (58.9, 62)	59.5 (58.1, 61.2)	1.5%
Northern Mariana Islands	High	248.2 (223.1, 278.7)	190 (173.6, 208.7)	30.6%	68.1 (66.7, 69.7)	52.2 (49, 55.6)	30.3%	64.1 (62.3, 66.4)	66.6 (63.7, 68)	-3.8%
Norway	High	179.9 (157.8, 207)	125.2 (116.2, 135.9)	43.7%	65.7 (64.1, 67.4)	45.7 (42, 49.7)	43.9%	72.5 (71.9, 73.1)	72.9 (72.4, 73.4)	-0.6%
Oman	High-middle	234.2 (200, 267.3)	174.5 (151.8, 197.9)	34.2%	66.7 (65.3, 68.2)	49.8 (46.6, 53.2)	34.0%	64.2 (62.3, 66.8)	66 (63.5, 68.3)	-2.7%
Pakistan	Low-middle	348.1 (303.9, 394.4)	298.3 (260.9, 341.1)	16.7%	67 (65, 68.7)	57.4 (54.1, 60.3)	16.6%	56.1 (54.5, 58.1)	56.1 (54.3, 58.1)	0.1%
Palestine	Middle	248.2 (223, 276.1)	183.5 (170.8, 197.4)	35.3%	68.8 (66.5, 71.1)	51 (47.1, 54.8)	35.0%	64.2 (63.3, 65)	65.1 (64.7, 65.9)	-1.5%
Panama	High-middle	168.5 (150.1, 188.9)	128.5 (118.1, 139.9)	31.1%	59.2 (57.9, 60.3)	45.2 (42.7, 47.6)	31.0%	73.6 (72.6, 74.6)	74.1 (73.2, 75)	-0.7%

Papua New Guinea	Low-middle	576.2 (513.1, 652.7)	506.6 (452.3, 576.1)	13.7%	66.5 (64.4, 68.7)	58.5 (55.9, 61.3)	13.7%	45.7 (42.8, 48)	45.6 (42.6, 48.2)	0.3%
Paraguay	Middle	224.8 (196.5, 258)	167.4 (147, 191.1)	34.3%	63 (61.5, 64.6)	46.9 (43.8, 50.2)	34.3%	66.3 (64, 68.5)	67.8 (65.3, 70.2)	-2.2%
Peru	High-middle	162.8 (139.3, 186.2)	123.8 (108.2, 139.5)	31.5%	60.3 (58.7, 61.8)	45.9 (42.7, 49)	31.5%	74.3 (72.4, 76.4)	74.3 (72.4, 76.5)	-0.1%
Philippines	Middle	331.1 (296.4, 369.6)	276.2 (247.7, 306.9)	19.9%	67.6 (66.2, 68.8)	56.3 (53.5, 59)	19.9%	58.9 (57, 61)	59.5 (57.5, 61.5)	-1.0%
Poland	High	243.2 (218.3, 271.6)	198 (183.7, 214.7)	22.8%	69.7 (68.3, 71)	56.7 (53.7, 59.7)	23.0%	63.8 (63, 64.7)	63.9 (63.1, 64.8)	-0.1%
Portugal	High-middle	189.1 (165.6, 215.8)	130.7 (121, 141.5)	44.7%	66.8 (65.2, 68.5)	46.3 (42.3, 50.6)	44.4%	72.4 (71.5, 73.3)	73.7 (72.8, 74.6)	-1.7%
Puerto Rico	High-middle	179.7 (160.7, 202.1)	128.9 (119.8, 138.8)	39.4%	56.3 (52.3, 58.3)	40.3 (36.9, 43.1)	39.8%	72.7 (71.7, 73.8)	74.6 (73.6, 75.1)	-2.5%
Qatar	High-middle	206 (175.6, 239.3)	137.5 (119.5, 156.9)	49.8%	67.2 (65.6, 69)	44.8 (41.6, 48.5)	49.9%	67.4 (65.4, 69.4)	70.3 (68.3, 73)	-4.2%
Romania	High-middle	284 (258.2, 313.8)	238.4 (222.9, 255.5)	19.1%	71.6 (70.1, 72.9)	60.1 (57.2, 63.3)	19.0%	61.2 (60.3, 62.1)	61 (60.1, 62)	0.3%
Russian Federation	High-middle	307.9 (285.9, 334.8)	266.4 (253.7, 281.2)	15.6%	62.6 (61.6, 63.5)	54.1 (51.9, 56.3)	15.6%	60.5 (59.4, 60.8)	59.2 (58.9, 59.5)	2.1%
Rwanda	Low	249.3 (221.1, 279.7)	203.5 (181.3, 227.3)	22.5%	51.8 (49.9, 53.7)	42.2 (39.8, 44.9)	22.7%	63.9 (62.2, 65.4)	64.1 (62.3, 65.8)	-0.4%
Saint Lucia	High-middle	233.3 (212.5, 255.1)	177.7 (166.4, 190.3)	31.3%	64 (62.7, 65.2)	48.7 (45.9, 51.4)	31.3%	65.8 (64.9, 66.8)	67.5 (66.5, 68.5)	-2.4%
Saint Vincent and the Grenadines	High-middle	271.3 (249.2, 294.7)	206.8 (194.5, 219.2)	31.2%	64 (62.8, 65.1)	48.8 (46.1, 51.3)	31.2%	62.9 (62.2, 63.7)	64.8 (63.9, 65.3)	-2.9%
Samoa	Middle	304 (269.6, 334.7)	238.2 (213, 260.3)	27.6%	70.1 (67.2, 71.9)	55.1 (51.2, 58.2)	27.2%	60.3 (58.8, 61.7)	61.8 (60.5, 63.4)	-2.5%
Sao Tome and Principe	Low-middle	299.4 (265.7, 334.1)	257.7 (231.1, 284.9)	16.2%	63 (61.1, 64.8)	54.2 (51.4, 56.9)	16.1%	61.1 (58.7, 62.7)	60.7 (58.2, 62.5)	0.6%
Saudi Arabia	High-middle	237 (209.5, 266.4)	183.2 (168.1, 203.3)	29.3%	65.7 (64.2, 67.6)	51 (47.6, 54.7)	28.7%	65.7 (64.1, 66.8)	66.3 (64.7, 67.3)	-0.8%
Senegal	Low	290.4 (261.9, 324.5)	242.1 (219.1, 269)	20.0%	58.5 (56.9, 60.4)	48.7 (46.3, 51.6)	20.0%	60.8 (58.8, 62.4)	61 (58.8, 62.7)	-0.3%
Serbia	High-middle	296.8 (270.2, 328.3)	245 (229, 261.9)	21.2%	75.2 (73.8, 76.7)	62.1 (58.8, 65.4)	21.1%	61.3 (60.6, 62)	61.6 (60.9, 62.4)	-0.5%
Seychelles	High-middle	276.1 (253.7, 300.7)	231.3 (217.9, 244.2)	19.4%	68.4 (67.1, 69.5)	57.3 (54.3, 60.1)	19.3%	62.4 (61.7, 63.1)	62.4 (61.7, 63.2)	-0.1%
Sierra Leone	Low	331.6 (293.8, 376)	284.9 (251.4, 322.7)	16.4%	54.2 (51.5, 56.7)	46.4 (43.2, 49.5)	16.9%	58.2 (55.9, 61.2)	58.1 (55.7, 61.2)	0.3%
Singapore	High	146.3 (127.5, 169)	108.3 (98.6, 119.9)	35.1%	67.1 (65.4, 69)	49.7 (45.9, 53.7)	35.0%	76.3 (75.4, 77.2)	76 (75.1, 76.9)	0.4%
Slovakia	High	256.4 (229.7, 287.3)	208.8 (193.7, 227.9)	22.8%	72.6 (71.2, 74.2)	59.1 (55.9, 62.4)	22.9%	63.5 (62.6, 64.3)	63.6 (62.8, 64.5)	-0.2%
Slovenia	High	202.8 (177.8, 233.3)	154.4 (139.7, 172.4)	31.4%	68.2 (66.9, 69.4)	52 (48.9, 55.2)	31.0%	69.7 (68.4, 70.6)	70 (68.9, 71)	-0.4%
Solomon Islands	Low-middle	443.9 (397.7, 494.4)	368 (330.3, 405.5)	20.6%	70.3 (68.2, 71.7)	58.4 (55.6, 60.9)	20.5%	52.1 (49.9, 54)	53.4 (51.1, 55.1)	-2.5%
Somalia	Low	362.7 (301.2, 434.7)	311.4 (255.2, 382.6)	16.5%	52.2 (50, 54.6)	44.8 (42.1, 47.5)	16.5%	56 (53, 59.5)	56 (52.7, 59.6)	0.0%
South Africa	Middle	263 (242.5, 287.1)	204.2 (194.1, 215.8)	28.8%	42.2 (40.3, 43.8)	32.7 (30.9, 34.3)	29.0%	62.3 (61.8, 62.7)	64.2 (63.7, 64.8)	-3.1%
South Korea	High	158.3 (137.9, 182.6)	110.1 (100.7, 120.4)	43.8%	62.7 (61.5, 63.9)	43.6 (40.3, 46.7)	43.8%	74.4 (73.6, 75.2)	75.1 (74.4, 75.8)	-0.9%
South Sudan	Low	329.1 (274.7, 403.6)	278.3 (227.9, 349.4)	18.3%	49.5 (44.3, 52.5)	41.8 (37.1, 45.6)	18.4%	57.6 (54.4, 62.2)	57.7 (54.3, 62.6)	-0.3%
Spain	High-middle	163.5 (144.3, 187)	119.2 (110.1, 130)	37.1%	65.8 (63.7, 67.9)	47.9 (44, 52.1)	37.2%	75 (74.2, 75.7)	75.1 (74.4, 75.8)	-0.1%
Sri Lanka	High-middle	202.3 (175.9, 234.3)	151 (132.3, 173)	34.0%	62.3 (60.9, 63.5)	46.5 (43.6, 49.3)	34.0%	68.4 (66.4, 71.1)	70.4 (67.8, 72.6)	-2.9%
Sudan	Low-middle	294 (253.6, 338.4)	240.9 (205.2, 278.9)	22.0%	65.7 (63.2, 67.9)	53.9 (49.7, 57.9)	22.0%	61.1 (58, 63.9)	61.2 (58.1, 64.2)	-0.3%
Suriname	High-middle	261 (234.6, 289.7)	206.1 (187.2, 224.3)	26.6%	61.9 (60.8, 62.9)	48.8 (46.2, 51.2)	26.9%	62.9 (61.3, 64.7)	64.1 (62.3, 66)	-1.8%
Swaziland	Middle	392.7 (339.8, 455)	306.7 (263.8, 355.7)	28.0%	47.3 (44.7, 49.9)	36.9 (34.2, 39.4)	28.1%	54.5 (51.9, 56.5)	56 (53.3, 59)	-2.8%
Sweden	High	172.5 (150.1, 199.3)	122.1 (112.4, 133.3)	41.3%	65.3 (63.7, 67.1)	46.3 (42.8, 50.2)	41.2%	73 (72.2, 73.9)	73.3 (72.6, 74.2)	-0.4%
Switzerland	High	161.6 (138.7, 188.4)	104.9 (95.7, 115.5)	54.0%	66.1 (64.6, 67.6)	43 (39.3, 47.2)	53.8%	75.6 (74.8, 76.4)	76.1 (75.4, 76.7)	-0.6%
Syria	Middle	261.4 (227.1, 294.2)	213.1 (186, 240.1)	22.7%	54.1 (49.7, 57.8)	44 (39.9, 48.4)	23.0%	63.6 (61.4, 65.6)	63.5 (61.2, 65.6)	0.1%
Taiwan	High	184.8 (167.1, 204.4)	146.3 (136, 156.8)	26.3%	62.9 (61.4, 64.2)	49.7 (46.7, 52.6)	26.5%	72.1 (71.4, 72.8)	72.4 (71.7, 73.1)	-0.4%
Tajikistan	Middle	300.9 (273.2, 332.1)	251.9 (231.7, 272.5)	19.4%	71.1 (69.3, 72.7)	59.5 (56.1, 62.6)	19.5%	59.7 (58.4, 61.3)	60.5 (58.9, 61.6)	-1.4%
Tanzania	Low-middle	256.7 (230.1, 285.5)	212.1 (193.1, 233.5)	21.0%	52.2 (50.1, 54.4)	43 (40.6, 45.5)	21.3%	63 (61.5, 64.5)	63.1 (61.5, 64.6)	-0.2%
Thailand	High-middle	186.3 (165.5, 208.6)	146.4 (132.6, 160.5)	27.3%	56.3 (55, 57.6)	44.3 (41.6, 46.8)	27.1%	72.3 (71, 74)	72.6 (71.4, 74.2)	-0.4%
The Bahamas	High	253.3 (230.1, 279.1)	205.8 (190.4, 223.6)	23.0%	61.9 (60.7, 62.9)	50.3 (47.7, 52.8)	23.0%	65.4 (63.1, 66.5)	65.9 (63.5, 67)	-0.7%
The Gambia	Low	323.1 (288.3, 362.5)	272.6 (245.2, 304.8)	18.5%	55.6 (53.4, 57.8)	46.9 (44.3, 49.7)	18.7%	58.2 (56.4, 60.4)	58.2 (56.4, 60.5)	0.0%
Timor-Leste	Low-middle	281.6 (241.5, 318.1)	238.3 (203, 264.2)	18.2%	63.9 (56.8, 66.1)	53.9 (46.9, 57.1)	18.6%	61.4 (59, 63.2)	61.3 (59, 63.2)	0.1%
Togo	Low	298.4 (260.9, 338.5)	250.7 (219.8, 286.8)	19.0%	52.5 (50, 54.8)	44.2 (41.3, 47)	18.8%	60.4 (57.4, 62.5)	60.3 (57.2, 62.5)	0.1%
Tonga	Middle	315.8 (282.2, 353.3)	227.9 (205.7, 252.8)	38.6%	68.3 (66.8, 69.6)	49.3 (46.7, 51.9)	38.6%	59.1 (57.1, 60.8)	63.2 (61.4, 64.8)	-6.5%
Trinidad and Tobago	High	250.5 (215.4, 292.1)	175.2 (150, 205.1)	43.0%	64.1 (62.7, 65.4)	44.9 (41.9, 47.7)	42.7%	63.5 (60.7, 65.8)	67.3 (64.6, 70.6)	-5.7%

Tunisia	Middle	221.9 (188.6, 259.9)	170.4 (144.5, 197.5)	30.2%	66.9 (65, 69.1)	51.4 (47, 55.9)	30.3%	67.4 (65.1, 69.9)	68 (65.6, 70.5)	-0.9%
Turkey	High-middle	210.1 (186.8, 236.5)	157.7 (144.6, 169.7)	33.2%	68.2 (66.1, 70.3)	51.1 (47.3, 55.3)	33.5%	67.1 (66, 68.3)	67.9 (66.7, 69.2)	-1.2%
Turkmenistan	Middle	335 (307.2, 364.8)	287.6 (269.2, 307.7)	16.5%	71.1 (69.7, 72.3)	61.1 (58.2, 63.9)	16.4%	57.7 (56.8, 58.6)	57.7 (56.8, 58.6)	-0.1%
Uganda	Low	272.9 (240.5, 303.9)	222.3 (197.1, 247)	22.8%	51.3 (48.9, 53.5)	41.8 (39.1, 44.6)	22.7%	62.2 (60.6, 64)	62.5 (60.9, 64.5)	-0.5%
Ukraine	High-middle	350.4 (323.2, 377.5)	301.4 (284, 318.7)	16.3%	66.2 (64.9, 67.2)	57 (54.3, 59.6)	16.1%	57.6 (56.8, 58.4)	57.4 (56.6, 58.1)	0.4%
United Arab Emirates	High	301.4 (259.8, 347.5)	240.4 (206.9, 277.5)	25.4%	67.6 (64.3, 69.8)	53.9 (49.5, 57.5)	25.5%	57.5 (55.9, 60.9)	58 (56.1, 61.6)	-0.9%
United Kingdom	High	194.7 (172.7, 221.6)	143.3 (134.1, 154)	35.9%	66.1 (64.6, 67.7)	48.7 (44.9, 52.7)	35.8%	70.7 (70.3, 71.2)	70.8 (70.5, 71.1)	-0.1%
United States	High	215.9 (193.5, 240.4)	161.5 (152, 171.4)	33.7%	61.5 (60.3, 62.6)	46 (43, 49.2)	33.6%	67.6 (67, 68.2)	68.5 (68, 68.9)	-1.3%
Uruguay	High-middle	224.3 (199.9, 251.4)	173.8 (157.8, 192.2)	29.1%	65.7 (64.2, 66.9)	51 (47.4, 54.4)	28.7%	66.2 (64.5, 67.9)	66.6 (65, 68.3)	-0.7%
Uzbekistan	High-middle	365.8 (331, 401.8)	312.6 (285.5, 340.8)	17.0%	73.2 (71.6, 74.7)	62.8 (59.4, 65.7)	16.6%	57.3 (56.2, 58.5)	57.8 (56.6, 59)	-0.8%
Vanuatu	Low-middle	457.2 (389.8, 538.9)	392.1 (332, 469.3)	16.6%	68.8 (65.3, 70.9)	59 (54.9, 62.1)	16.7%	51.8 (47.2, 54.9)	52.2 (47.2, 55.5)	-0.8%
Venezuela	High-middle	213.4 (189, 242.9)	169.6 (152.1, 192)	25.8%	60.5 (59.1, 61.8)	48.1 (45.5, 50.5)	25.8%	67.3 (65.1, 69.5)	67.9 (65.7, 70.2)	-0.9%
Vietnam	Middle	241.2 (217.3, 270.1)	197.7 (180.7, 219.9)	22.0%	64.3 (63.1, 65.6)	52.8 (50, 55.7)	21.8%	63.9 (62.1, 65.3)	64.1 (62.1, 65.5)	-0.3%
Virgin Islands, U.S.	High-middle	262.7 (229.8, 292.3)	213.2 (185.7, 233.9)	23.2%	65.4 (63.7, 66.8)	53 (49.8, 55.9)	23.5%	61.9 (60.6, 64.9)	62.3 (61, 65.4)	-0.7%
Yemen	Low-middle	330.4 (274.7, 398.9)	280.6 (229.5, 342.2)	17.7%	63.4 (60.3, 65.8)	53.8 (49.3, 57.6)	17.7%	58.3 (54.7, 61.5)	58.1 (54.5, 61.5)	0.3%
Zambia	Low-middle	292.7 (261.6, 324.1)	243.7 (218.5, 268.7)	20.1%	47.7 (45.5, 49.7)	39.6 (37.2, 42.1)	20.2%	60.9 (58.8, 62.6)	61.2 (59.1, 63)	-0.5%
Zimbabwe	Low-middle	355.8 (317, 401.8)	300.6 (268.4, 338.6)	18.4%	49.9 (47.4, 51.8)	42 (39.5, 44.3)	18.7%	55.7 (54.4, 57.9)	56 (54.5, 58.4)	-0.5%

6. References

1 Searle SD, Mitnitski A, Gahbauer EA, Gill TM, Rockwood K. A standard procedure for creating a frailty index. *BMC Geriatr* 2008; **8**. DOI:10.1186/1471-2318-8-24.

2 Campisi J. Aging, Cellular Senescence, and Cancer. *Annu Rev Physiol* 2013; **75**: 685–705.

3 Campisi J, Robert L. Cell Senescence: Role in Aging and Age-Related Diseases. In: Robert L, Fulop T, eds. Interdisciplinary Topics in Gerontology. Basel: S. KARGER AG, 2014: 45–61.

4 Patton GC, Sawyer SM, Santelli JS, *et al.* Our future: a Lancet commission on adolescent health and wellbeing. *The Lancet* 2016; **387**: 2423–78.

5 Patton GC, Olsson CA, Skirbekk V, *et al.* Adolescence and the next generation. *Nature* 2018; **554**: 458.

6 Murray CJL, Callender CSKH, Kulikoff XR, *et al.* Population and fertility by age and sex for 195 countries and territories, 1950–2017: a systematic analysis for the Global Burden of Disease Study 2017. *The Lancet* 2018; **392**: 1995–2051.

7 Dorsey ER, Elbaz A, Nichols E, *et al.* Global, regional, and national burden of Parkinson's disease, 1990–2016: a systematic analysis for the Global Burden of Disease Study 2016. Supplemental Appendix. *Lancet Neurol* 2018; **17**: 939–53.

8 Salomon JA, Haagsma JA, Davis A, *et al.* Disability weights for the Global Burden of Disease 2013 study. *Lancet Glob Health* 2015; **3**: e712–23.

9 James SL, Abate D, Abate KH, *et al.* Global, regional, and national incidence, prevalence, and years lived with disability for 354 diseases and injuries for 195 countries and territories, 1990–2017: a systematic analysis for the Global Burden of Disease Study 2017. *The Lancet* 2018; **392**: 1789–858.

10 Hodes RJ, Sierra F, Austad SN, *et al.* Disease drivers of aging: Disease drivers of aging. *Ann N Y Acad Sci* 2016; **1386**: 45–68.

11 Prasad S, Sung B, Aggarwal BB. Age-associated chronic diseases require age-old medicine: Role of chronic inflammation. *Prev Med* 2012; **54**: S29–37.

12 The Alliance for Aging Research. The-Silver-Book_Chronic-Disease-and-Medical-Innovation-in-an-Aging-Natio....pdf. www.silverbook.org (accessed July 27, 2018).
